# A One-Pot Tandem Strategy in Catalytic Asymmetric Vinylogous Aldol Reaction of Homoallylic Alcohols

**DOI:** 10.3390/molecules21070842

**Published:** 2016-06-27

**Authors:** Xufeng Hou, Zhenzhong Jing, Xiangbin Bai, Zhiyong Jiang

**Affiliations:** 1 School of Chemistry and Chemical Engineering, Xuchang University, Xuchang 461000, China; hxfst@eyou.com; 2 Key Laboratory of Natural Medicine and Immuno-Engineering of Henan Province, Henan University, Jinming Campus, Kaifeng 475004, Henan, China; jzzhenu@163.com (Z.J.); bxb1990@163.com (X.B.)

**Keywords:** asymmetric organocatalysis, vinylogous aldol reactions, oxidation, cascade, homoallylic alcohols

## Abstract

Reported is a rationally-designed one-pot sequential strategy that allows homoallylic alcohols to be employed in a catalytic, asymmetric, direct vinylogous aldol reaction with a series of activated acyclic ketones, including trifluoromethyl ketones, γ-ketoesters, and α-keto phosphonates, in high yields (up to 95%) with excellent regio- and enantio-selectivity (up to 99% *ee*). This modular combination, including Jones oxidation and asymmetric organocatalysis, has satisfactory compatibility and reliability even at a 20 mmol scale, albeit without intermediary purification.

## 1. Introduction

Asymmetric vinylogous aldol (AVA) reaction allows expedient generation of chiral multifunctional alcohols with an extended carbon skeleton and α,β-unsaturated group, which is easily further transformed into structurally complex molecules with divergent synthetic targets [1,2,3,4,5]. As such, the development of the AVA reaction has spurred research in this field in the past few decades [1,2,3,4,5,6,7,8,9,10,11,12,13,14,15,16,17,18,19]. Inspired by the atom economy philosophy, the AVA variant in which direct employment of unmasked nucleophiles to facilitate the generation of dienolates is our current focus [6,7,8,9,10,11,12,13,14,15,16,17,18,19].

We disclosed a direct AVA reaction of allyl ketones to isatins back in 2013, leading to the divergent synthesis of biologically-important 3-hydroxy-2-oxindoles derivatives [14]. Since then, a series of direct asymmetric vinylogous reactions using allyl ketones as nucleophiles have been carried out successfully with excellent γ-selectivity and enantioselectivity [17,18,19]. For example, Chen and co-workers developed a catalytic asymmetric vinylogous Michael addition of allyl ketones to maleimides through dienamine catalysis [17]. Xu et al. reported another efficient asymmetric vinylogous Michael addition between allyl ketones and α,β-unsaturated aldehydes by employing a multifunctional supramolecular iminium catalyst [18]. Very recently, our group introduced a highly-enantioselective vinylogous aldol reaction of allyl ketones to activated acyclic ketones, such as trifluoromethyl ketones, α-ketoesters, and α-keto phosphonates, developing expedient and divergent methods to access valuable chiral electron-withdrawing group-substituted tertiary hydroxyl-based carboxylic acids [19]. These progressive contributions highlight the versatility of allyl ketone nucleophiles in asymmetric synthesis.

The oxidation of homoallylic alcohols is known as the most direct and efficient method to synthesize allyl ketones. However, previous works revealed that yields of allyl ketones were non-quantitative (less than 70%; most gave poor conversions) through Jones oxidation or Dess-Martin oxidation [20,21,22]. In the course of our studies, allyl ketones were observed to easily isomerize to the more stable activated α,β-alkenes during of purification and storage [14,19]. The relative instability of allyl ketones means a major fraction of it forms the more stable precursor before vinylogous reactions, and complicating the process for large-scale synthesis.

The “one-pot” tandem strategy is known to sequentially perform multiple chemical transformations in a single reaction vessel without intermediary purification steps [23,24,25,26,27,28]. Owing to the significant potential in scale-up production, as well as saving costs, time, and waste generation, it has been recognized as one of the most promising paradigms in both industry and pharmaceuticals [23,24,25,26,27,28]. As an extension of our work towards direct asymmetric vinylogous reactions, we envisaged developing a tandem one-pot protocol involving both oxidation and asymmetric organocatalysis. The in situ generation of allyl ketone from homoallylic alcohol is fed directly into AVA, thus skipping intermediate purification.

## 2. Results and Discussion

To probe the feasibility of this one-pot asymmetric method, we initiated our study with the model reaction of homoallylic alcohol **1a** with trifluoromethyl acetophenone **2a** (Table 1). The Dess-Martin oxidation of homoallylic alcohol **1a** in CH_2_Cl_2_ at 25 °C completed within 20 min, affording the corresponding allyl ketone **3a** with full conversion. However there was no reaction after adding **2a** under the AVA reaction conditions: 10 mol % of catalyst **4**, 2.0 equiv. of Na_3_PO_4_ in *t*BuPh at −10 °C [19]. Subsequent Jones oxidation gave a cloudy reaction mixture requiring extraction and flash chromatography purification. After careful selection of diverse benzenes (such as benzene, toluene, chlorobenzene, and so on) as the solvent, only *tert*-butylbenzene was found to present a clear separation between the organic and aqueous phases, and the tandem Jones oxidation and direct AVA reaction of **1a** can provide **5a** in 80% yield and 95% *ee* after 38 h.

With optimized reaction conditions, we began assessing the potential of this one-pot sequential protocol in the reaction between homoallylic alcohols **1** and trifluoromethyl ketones **2**. As shown in Table 2, the corresponding adduct **5** was obtained in 44%–95% yields with *ee* of 73%–99%. It should be noted that allyl ketones **1** with *ortho*-substituted phenyl groups gave deteriorated enantiomeric excesses (Table 2, entries 18 and 23), whereas allyl 2-thienyl ketone presented **5y** with outstanding 98% *ee* (Table 2, entry 25). This is in contrast with the results of the AVA reaction between allyl ketones and trifluoromethyl ketones as reported [19], and overall yields were increased dramatically. A plausible mechanism for this asymmetric vinylogous aldol reaction is proposed via the transition-state model in the Supplementary Materials.

**Scheme 1:**
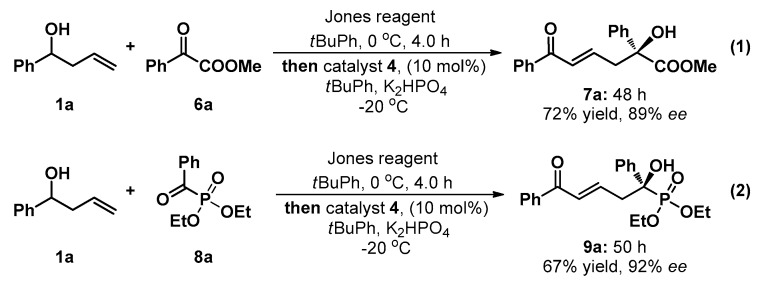
Expansion of substrate scope: (**1**) the reaction of **1a** to methyl benzoylformate **6a**; (**2**) the reaction of **1a** to diethyl benzoylphosphonate **8a**.

**Scheme 2:**
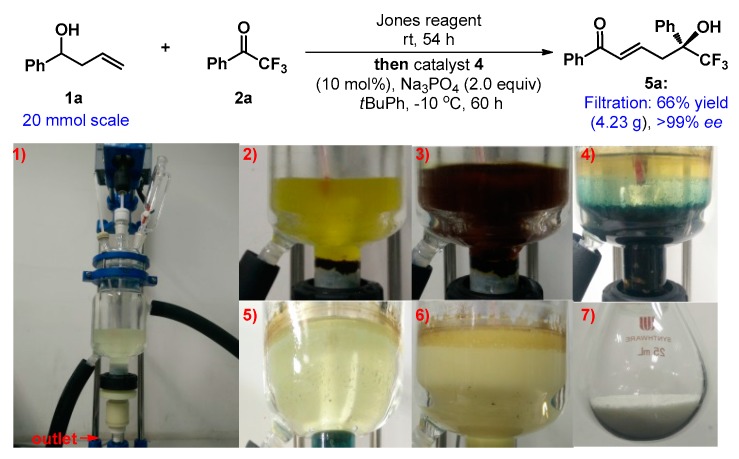
The one-pot tandem synthesis of **5a** from **1a** and **2a** in a 20 mmol scale: *flash chromatography*: 79% yield (5.07 g), 95% *ee*; *filtration and wash*: 66% yield (4.23 g), >99% *ee*. Images (IMG): (**1**) **5a** in *t*BuPh (500 mL container); (**2**): initiation of Jones oxidation; (**3**): duration of oxidation; (**4**): after oxidation (a clear stratification between organic and aqueous phase); (**5**): after aqueous phase was released; (**6**): after AVA reaction of **2a** (white precipitant is **5a**); (**7**): product **5a** with >99% *ee* (after filtrated and washed with cool hexane, white solid).

The established protocol was also used in vinylogous aldol reactions of vinyl ketones with α-ketoesters and α-keto phosphonates. Representative methyl benzoylformate **6a** was selected to react with homoallylic alcohol **1a** under the established reaction conditions. After the complete oxidation of **1a**, asymmetric reaction was performed in the presence of 10 mol % of catalyst **4** and 2.0 equiv. of K_2_HPO_4_ as the acid-capturer in *t*BuPh at −20 °C (Scheme 1, (1)) It was found that the reaction was done in 48 h, affording the desired vinylogous aldol adduct **7a** in 72% yield with 89% *ee*. Another activated acyclic ketones, diethyl benzoylphosphonate **8a** was also attempted (Scheme 1, (2)) and the γ-selective adduct **9a** was isolated in 67% yield with 92% *ee* after 50 h.

To demonstrate the synthetic value of this work, the sequential Jones oxidation/AVA reaction of homoallylic alcohol **1a** with trifluoromethyl acetophenone **2a** was conducted in 20 mmol or gram-scale (Scheme 2). Due to the scale-up, a longer reaction time of 54 h was necessary to fully oxidize **1a** with Jones reagent. After removal of the aqueous phase, the AVA reaction with **2a** was completed within 60 h, affording the product **5a** in 79% yield with 95% *ee* after flash chromatography. During the course of reaction, the product **5a** precipitated out; thus, a convenient filtration approach [20] could be carried out. Enantiopure **5a** (>99% *ee*) as a white powder was obtained in 66% yield after filtration and rinsing with cold hexane (IMG-7, Scheme 2). This protocol, thus, has good potential in industry.

## 3. Materials and Methods

Experiments involving moisture and/or air sensitive components were performed under a positive pressure of nitrogen in oven-dried glassware equipped with a rubber septum inlet. Dried solvents and liquid reagents were transferred by oven-dried syringes or hypodermic syringe cooled to ambient temperature in a desiccator. Reaction mixtures were stirred in 10 mL sample vials with Teflon-coated magnetic stirring bars unless otherwise stated. Moisture in non-volatile reagents/compounds was removed in high vacuum by means of an oil pump and subsequent purging with nitrogen. Solvents were removed in vacuo under ~30 mmHg and heated with a water bath at 30–35 °C using a rotary evaporator with an aspirator. The condenser was cooled with running water at 0 °C.

All experiments were monitored by analytical thin layer chromatography (TLC). TLC was performed on pre-coated plates, 60 F254. After elution, each plate was visualized under UV illumination at 254 nm for UV-active material. Further visualization was achieved by staining with KMnO_4_, ceric molybdate, or anisaldehyde solution. For those using the aqueous stains, the TLC plates were heated on a hot plate.

Columns for flash chromatography (FC) contained 200–300 mesh silica gel. Columns were packed as slurry of silica gel in petroleum ether and equilibrated solution using the appropriate solvent system. The elution was assisted by applying pressure of about 2 atm with an air pump.

Proton nuclear magnetic resonance (^1^H-NMR) and carbon NMR (^13^C-NMR) spectra were recorded in CDCl_3_, unless otherwise stated. Chemical shifts are reported in parts per million (ppm), using the residual solvent signal as an internal standard: CDCl_3_ (^1^H-NMR: δ 7.26, singlet; ^13^C-NMR: δ 77.0, triplet). Multiplicities were given as: s (singlet), d (doublet), t (triplet), q (quartet), quintet, m (multiplets), dd (doublet of doublets), dt (doublet of triplets), and br (broad). Coupling constants (*J*) were recorded in Hertz (Hz). The number of proton atoms (n) for a given resonance was indicated by nH. The number of carbon atoms (n) for a given resonance was indicated by *n*C. HRMS was reported in units of mass of charge ratio (*m*/*z*). Mass samples were dissolved in CH_3_CN (HPLC grade) unless otherwise stated. Melting points were determined on a melting point apparatus.

Enantiomeric excesses were determined by chiral high-performance liquid chromatography (HPLC) analysis. UV detection was monitored at 254 nm, 230 nm, and 210 nm at the same time. HPLC samples were dissolved in HPLC grade isopropanol (IPA), unless otherwise stated.

All commercial reagents were purchased with the highest purity grade. They were used without further purification unless specified. All solvents used, mainly petroleum ether (PE) and ethyl acetate (EtOAc), were distilled. Anhydrous DCM was freshly distilled from CaH_2_ and stored under N_2_ atmosphere. *tert*-butylbenzene was freshly distilled from sodium/benzophenone before use. All compounds synthesized were stored in a −20 °C freezer and light-sensitive compounds were protected with aluminum foil.

### 3.1. General Experimental Procedure for the One-Pot Tandem Direct Asymmetric Vinylogous Aldol Reaction of Allyl Ketones ***1*** to Trifluoromethyl Ketones ***2***

Jones reagent (0.25 mL) was added dropwise to a solution of homoallic alcohols **1** (0.4 mmol, 4.0 equiv.) in *tert*-butylbenzene (1.0 mL) at 0 °C over a period of 3.5–7 h. When the reaction was completed (monitored by TLC), the sample was stewed for a moment until a clear separation between the organic and aqueous phase was formed, and the aqueous phase was released. Then the trifluoromethyl ketone **2** (0.1 mmol, 1.0 equiv.) was added, the reaction mixture was stirred at −10 °C for 10 min. Sodium phosphate (0.2 mmol, 2.0 equiv.) and catalyst **4** (0.01 mmol, 0.1 equiv.) were added sequentially (10 min interval). The reaction mixture was stirred at −10 °C and monitored by TLC. Upon complete consumption of trifluoromethyl ketone **2**, the reaction mixture was directly loaded onto a short silica column, followed by gradient elution with PE/EA mixture (20/1–5/1 ratio). Removing the solvent in vacuum afforded products **5a**–**y**.

### 3.2. General Experimental Procedure for the One-Pot Tandem Direct Asymmetric Vinylogous Aldol Reaction of Homoallylic Alcohol ***1a*** to Acyclic Activated Ketones (***6a*** and ***8a***)

Jones reagent (0.45 mL) was added dropwise to a solution of homoallic alcohol **1a** (0.4 mmol, 4 equiv.) in *tert*-butylbenzene (800 μL) at 0 °C over a period of 4 h. When the reaction was completed (monitored by TLC), the sample was stilled for a moment, and the aqueous phase was released. The reaction mixture was stirred at −20 °C for 10 min. Potassium phosphate anhydrous (0.2 mmol, 2.0 equiv.), catalyst **4** (0.01 mmol, 0.1 equiv.), and **6a**/**8a** (0.1 mmol, 1.0 equiv.) were added sequentially (10 min interval). The reaction mixture was stirred at −20 °C and monitored by TLC. Upon complete consumption of acyclic activated ketones **6a**/**8a**, the reaction mixture was directly loaded onto a short silica column, followed by gradient elution with PE/EA mixture (20/1–1/1 ratio). Removing the solvent in vacuum afforded products **7a**/**9a**.

*(−)-(S,E)-6,6,6-Trifluoro-5-hydroxy-1,5-diphenylhex-2-en-1-one* (**5a**): White solid, Mp 133.7–135.2 °C; 25.6 mg (0.1 mmol), 80% yield; 95% *ee*; ${\left[\alpha \right]}_{D}^{26}$ −36.4 (*c* 2.47, CHCl_3_); ^1^H-NMR (300 MHz, CDCl_3_) δ 7.75 (d, *J* = 7.4 Hz, 2H), 7.59–7.51 (m, 3H), 7.46–7.33 (m, 5H), 6.91 (d, *J* = 15.5 Hz, 1H), 6.76–6.66 (m, 1H), 3.25–3.05 (m, 2H), 3.16 (s, 1H); ^13^C-NMR (75 MHz, CDCl_3_) δ 190.4, 140.6, 137.1, 135.8, 133.0, 130.7, 128.9, 128.6 (two peaks), 128.5, 126.3, 125.2 (q, *J* = 284.0 Hz), 77.7 (q, *J* = 28.2 Hz), 39.0; ^19^F-NMR (376 MHz, CDCl_3_) δ −79.55; HRMS (ESI) *m*/*z* 343.0928 [M + Na^+^], calc. for C_18_H_15_F_3_O_2_Na 343.0922. The *ee* was determined by HPLC analysis. CHIRALPAK IB (4.6 mm i.d. × 250 mm); Hexane/2-propanol = 90/10; flow rate 1.0 mL/min; 25 °C; 254 nm; retention time: 10.2 min (minor) and 11.2 min (major).

*(−)-(S,E)-6,6,6-Trifluoro-5-hydroxy-1-phenyl-5-(4-(trifluoromethyl)phenyl)hex-2-en-1-one* (**5b**): White solid, Mp 88.2–90.9 °C; 33.8 mg (0.1 mml), 87% yield; 92% *ee*; ${\left[\alpha \right]}_{D}^{26}$ –26.3 (*c* 2.47, CHCl_3_); ^1^H-NMR (300 MHz, CDCl_3_) δ 7.75–7.66 (m, 6H), 7.51 (t, *J* = 7.4 Hz, 1H), 7.41 (t, *J* = 7.8Hz, 2H), 6.92 (d, *J* = 15.5 Hz, 1H), 6.75–6.65 (m, 1H), 3.59 (s, 1H), 3.17 (m, 2H); ^13^C-NMR (75 MHz, CDCl_3_) δ 190.3, 140.0, 139.7, 136.9, 133.3, 131.8, 131.3, 130.9, 130.6, 130.4, 129.2 (two peaks), 128.6, 128.2, 127.0, 126.8, 125.6 (two peaks), 125.5 (two peaks), 125.4, 123.0, 122.0, 118.4, 77.2, 76.8, 76.4, 76.0, 39.0; ^19^F-NMR (376 MHz, CDCl_3_) δ −62.77, −79.37; HRMS (ESI) *m*/*z* 411.0797 [M + Na^+^], calc. for C_19_H_14_F_6_O_2_Na 411.0796. The *ee* was determined by HPLC analysis. CHIRALPAK IB (4.6 mm i.d. × 250 mm); Hexane/2-propanol = 90/10; flow rate 1.0 mL/min; 25 °C; 254 nm; retention time: 10.9 min (minor) and 15.4 min (major).

*(−)-(S,E)-6,6,6-Trifluoro-5-(4-fluorophenyl)-5-hydroxy-1-phenylhex-2-en-1-one* (**5c**): White solid, Mp 107.0–108.3 °C; 24.0 mg (0.1 mmol), 71% yield; 94% *ee*; ${\left[\alpha \right]}_{D}^{26}$ −31.0 (*c* 2.42, CHCl_3_); ^1^H-NMR (300 MHz, CDCl_3_) δ 7.77 (d, 2H), 7.78–7.52 (m, 3H), 7.41 (t, *J* = 7.6 Hz, 2H), 7.09 (t, *J* = 8.64 Hz, 2H), 6.92 (d, *J* = 15.5 Hz, 1H), 6.76–6.66 (m, 1H), 3.47 (s, 1H), 3.22–3.04 (m, 2H); ^13^C-NMR (75 MHz, CDCl_3_) δ 190.3, 164.5 , 161.2, 140.3, 137.0, 133.2, 131.8, 131.7, 130.7, 128.6 (two peaks), 128.5 (two peaks), 128.4 (two peaks), 125.1 (q, *J* = 283.4 Hz), 123.2, 115.6, 115.4, 76.4 (q, *J* = 28.4 Hz), 38.9; ^19^F-NMR (376 MHz, CDCl_3_) δ −79.80, −112.84; HRMS (ESI) *m*/*z* 361.0829 [M + Na^+^], calc. for C_18_H_14_F_4_O_2_Na 361.0828. The *ee* was determined by HPLC analysis. CHIRALPAK IB-3 (4.6 mm i.d. × 250 mm); Hexane/2-propanol = 90/10; flow rate 1.0 mL/min; 25 °C; 254 nm; retention time: 11.2 min (major) and 12.6 min (minor).

*(−)-(S,E)-5-(4-Chlorophenyl)-6,6,6-trifluoro-5-hydroxy-1-phenylhex-2-en-1-one* (**5d**): White solid, Mp 110.4–111.6 °C; 34.8 mg (0.1 mmol), 98% yield; 94% *ee*; ${\left[\alpha \right]}_{D}^{26}$ −46.6 (*c* 3.41, CHCl_3_); ^1^H-NMR (300 MHz, CDCl_3_) δ 7.76 (d, *J* = 7.32 Hz, 2H), 7.53–7.50 (m, 3H), 7.44–7.37 (m, 4H), 6.91 (d, *J* = 15.51 Hz, 1H), 6.75–6.65 (m, 2H), 3.51 (s, 1H), 3.21–3.04 (m, 2H); ^13^C-NMR (75 MHz, CDCl_3_) δ 190.3, 140.1, 137.0, 135.0, 134.5, 133.2, 130.8, 128.8, 128.6 (two peaks), 127.9, 125.0 (q, *J* = 284.3 Hz), 76.5 (q, *J* = 28.5 Hz ), 38.9; ^19^F-NMR (376 MHz, CDCl_3_) δ −79.66; HRMS (ESI) *m*/*z* 377.0531 [M + Na^+^], calc. for C_18_H_14_ClF_3_O_2_Na 377.0532. The *ee* was determined by HPLC analysis. CHIRALPAK IB (4.6 mm i.d. × 250 mm); Hexane/2-propanol = 80/20; flow rate 1.0 mL/min; 25 °C; 254 nm; retention time: 6.8 min (minor) and 9.1 min (major).

*(−)-(S,E)-5-(4-Bromophenyl)-6,6,6-trifluoro-5-hydroxy-1-phenylhex-2-en-1-one* (**5e**): White solid, Mp 114.2–115.3 °C; 31.9 mg (0.1 mmol); 80% yield; 95% *ee*; ${\left[\alpha \right]}_{D}^{26}$ −54.5 (*c* 1.42, CHCl_3_); ^1^H-NMR (300 MHz, CDCl_3_) δ 7.75 (d, *J* = 7.4 Hz, 2H), 7.56–7.53 (m, 3H), 7.47–7.39 (m, 4H), 6.90 (d, *J* = 15.5 Hz, 1H), 6.74–6.65 (m, 1H), 3.50 (s, 1H), 3.20–3.03 (m, 2H); ^13^C-NMR (75 MHz, CDCl_3_) δ 190.3, 140.1, 137.0, 135.1, 133.2, 131.7, 130.8, 128.6 (two peaks), 128.2 (two peaks), 126.8, 124.9 (q, *J* = 284.2 Hz), 123.3, 76.5 (q, *J* = 28.4 Hz), 38.9; ^19^F-NMR (376 MHz, CDCl_3_) δ −79.64; HRMS (ESI) *m*/*z* 423.0009 [M + Na^+^], calc. for C_18_H_14_BrF_3_O_2_Na 423.0006. The *ee* was determined by HPLC analysis. CHIRALPAK IB (4.6 mm i.d. × 250 mm); Hexane/2-propanol = 80/20; flow rate 1.0 mL/min; 25 °C; 254 nm; retention time: 7.0 min (minor) and 12.3 min (major).

*(−)-(S,E)-6,6,6-Trifluoro-5-(3-fluorophenyl)-5-hydroxy-1-phenylhex-2-en-1-one* (**5f**): Colorless oil; 32.1 mg (0.1 mmol), 95% yield; 91% *ee*; ${\left[\alpha \right]}_{D}^{26}$ −41.5 (*c* 2.56, CHCl_3_); ^1^H-NMR (300 MHz, CDCl_3_) δ 7.78 (d, *J* = 7.3 Hz, 2H), 7.55 (t, *J* = 7.4 Hz, 2H), 7.45–7.32 (m, 5H), 7.08 (t, *J* = 7.4 Hz, 1H), 6.93 (d, *J* = 15.5 Hz, 1H), 6.75–6.65 (m, 1H), 3.25 (s, 1H), 3.21–3.04 (m, 2H); ^13^C-NMR (75 MHz, CDCl_3_) δ 190.3 (two peaks), 164.5, 161.2, 140.1, 140.0, 138.6, 138.5, 137.0, 133.2, 130.8, 130.2, 130.1, 128.6 (two peaks), 125.0 (q, *J* = 284.0 Hz), 122.0 (two peaks), 116.0, 115.7, 114.2, 113.8 (two peaks), 76.2 (two peaks), 75.8 (two peaks), 39.0; ^19^F-NMR (376 MHz, CDCl_3_) δ −79.51, −111.68; HRMS (ESI) *m*/*z* 339.1004 [M + H^+^], calc. for C_18_H_15_F_4_O_2_ 339.1008. The *ee* was determined by HPLC analysis. CHIRALPAK IB-3 (4.6 mm i.d. × 250 mm); Hexane/2-propanol = 90/10; flow rate 1.0 mL/min; 25 °C; 254 nm; retention time: 11.3 min (major) and 13.2 min (minor).

*(−)-(S,E)-5-(3-Chlorophenyl)-6,6,6-trifluoro-5-hydroxy-1-phenylhex-2-en-1-one* (**5g**): Colorless oil; 33.3 mg (0.1 mmol), 94% yield; 90% *ee*; ${\left[\alpha \right]}_{D}^{26}$ −35.6 (*c* 3.04, CHCl_3_); ^1^H-NMR (300 MHz, CDCl_3_) δ 7.77 (d, *J* = 7.3 Hz, 2H), 7.62 (s, 1H), 7.55 (t, *J* = 7.4 Hz, 1H), 7.47–7.32 (m, 5H), 6.92 (d, *J* = 15.5 Hz, 1H), 6.74–6.64 (m, 1H), 3.54 (s, 1H), 3.21–3.05 (m, 2H); ^13^C-NMR (75 MHz, CDCl_3_) δ 190.4, 140.0, 138.1, 137.0, 134.7, 133.2, 130.9, 129.8, 129.1, 128.7, 128.6, 126.9, 126.8, 124.9 (q, *J* = 284.1 Hz), 124.6 (two peaks), 76.4 (q, *J* = 28.4 Hz), 39.0; ^19^F-NMR (376 MHz, CDCl_3_) δ −79.48; HRMS (ESI) *m*/*z* 377.0533 [M + Na^+^], calc. for C_18_H_14_ClF_3_O_2_ 377.0532. The *ee* was determined by HPLC analysis. CHIRALPAK IA (4.6 mm i.d. × 250 mm); Hexane/2-propanol = 95/05; flow rate 1.0 mL/min; 25 °C; 254 nm; retention time: 9.8 min (major) and 11.8 min (minor).

*(−)-(S,E)-6,6,6-Trifluoro-5-(2-fluorophenyl)-5-hydroxy-1-phenylhex-2-en-1-one* (**5h**): White solid, Mp 106.9–107.2 °C; 24.0 mg (0.1 mmol), 71% yield; 99% *ee*; ${\left[\alpha \right]}_{D}^{26}$ −26.5 (*c* 2.25, CHCl_3_); ^1^H-NMR (300 MHz, CDCl_3_) δ 7.77–7.74 (m, 2H), 7.70–7.64 (m, 1H), 7.53 (t, *J* = 7.4 Hz, 1H), 7.42–7.34 (m, 3H), 7.21 (td, *J* = 7.9, 1.1 Hz, 1H), 7.13–7.06 (m, 1H), 7.00 (d, *J* = 15.5 Hz, 1H), 6.85–6.75 (m, 1H), 3.76 (d, *J* = 5.0 Hz, 1H), 3.60–3.52 (m, 1H), 3.08–3.00 (m, 1H); ^13^C-NMR (75 MHz, CDCl_3_) δ 190.5, 161.7, 158.4, 141.0, 137.2, 133.0, 131.4, 131.3, 130.4, 130 (two peaks), 128.6, 128.5, 124.9 (qd, *J* = 284.4, 1.7 Hz), 124.5 (two peaks), 122.4, 122.3, 116.8, 116.4, 76.2 (qd, *J* = 30, 3.6 Hz), 37.5, 37.4; ^19^F-NMR (376 MHz, CDCl_3_) δ −80.65, −80.69, −111.46, −111.50; HRMS (ESI) *m*/*z* 339.1004 [M + H^+^], calc. for C_18_H_15_F_4_O_2_ 339.1008. The *ee* was determined by HPLC analysis. CHIRALPAK IA (4.6 mm i.d. × 250 mm); Hexane/2-propanol = 95/05; flow rate 1.0 mL/min; 25 °C; 254 nm; retention time: 9.4 min (major) and 11.9 min (minor).

*(−)-(S,E)-6,6,6-Trifluoro-5-hydroxy-1-phenyl-5-(p-tolyl)hex-2-en-1-one* (**5i**): White solid, Mp 109.2–110.6 °C; 20.1 mg (0.1 mmol), 60% yield; 95% *ee*; ${\left[\alpha \right]}_{D}^{26}$ −51.2 (*c* 1.32, CHCl_3_); ^1^H-NMR (300 MHz, CDCl_3_) δ 7.78–7.75 (m, 2H), 7.54 (t, *J* = 7.4 Hz, 1H), 7.46–7.38 (m, 4H), 7.22 (d, *J* = 8.1 Hz, 2H), 6.91 (d, *J* = 15.5 Hz, 1H), 6.76–6.66 (m, 1H), 3.23–3.02 (m, 2H), 2.97 (d, *J* = 5.9 Hz, 1H), 2.37 (s, 3H); ^13^C-NMR (75 MHz, CDCl_3_) δ 190.3, 140.6, 138.8, 137.2, 133.0, 132.9, 130.7, 129.3, 128.6, 128.5, 127.1, 125.2 (q, *J* = 284.2 Hz), 76.7 (q, *J* = 28.2 Hz), 39.0, 21.0; ^19^F-NMR (376 MHz, CDCl_3_) δ −79.74; HRMS (ESI) *m*/*z* 357.1077 [(M + Na^+^], calc. for C_19_H_17_F_3_O_2_Na 357.1078. The *ee* was determined by HPLC analysis. CHIRALPAK IB (4.6 mm i.d. × 250 mm); Hexane/2-propanol = 80/20; flow rate 1.0 mL/min; 25 °C; 254 nm; retention time: 6.7 min (minor) and 10.8 min (major).

*(−)-(S,E)-6,6,6-Trifluoro-5-hydroxy-1-phenyl-5-(m-tolyl)hex-2-en-1-one* (**5j**): White solid, Mp 78.5–80.4 °C; 22.4 mg (0.1 mmol), 67% yield; 94% *ee*; ${\left[\alpha \right]}_{D}^{26}$ −45.8 (*c* 1.84, CHCl_3_); ^1^H-NMR (300 MHz, CDCl_3_) δ 7.78–7.74 (m, 2H), 7.54 (t, *J* = 7.4 Hz, 1H), 7.43–7.28 (m, 5H), 7.19 (d, *J* = 7.2 Hz, 1H), 6.92 (d, *J* = 15.5 Hz, 1H), 6.76–6.66 (m, 1H), 3.24–3.06 (m, 2H), 3.02, (s, 1H) 2.38 (s, 3H); ^13^C-NMR (75 MHz, CDCl_3_) δ 190.4, 140.7, 138.3, 137.2, 135.8, 133.0, 130.8, 129.7, 128.7, 128.6, 128.5, 127.0 (two peaks), 125.2 (q, *J* = 284.0 Hz), 123.4 (two peaks), 77.7 (q, *J* = 28.2 Hz), 39.1, 21.6; ^19^F-NMR (376 MHz, CDCl_3_) δ −79.99; HRMS (ESI) *m*/*z* 357.1087 [M + Na^+^], calc. for C_19_H_17_F_3_O_2_Na 357.1078. The *ee* was determined by HPLC analysis. CHIRALPAK IB (4.6 mm i.d. × 250 mm); Hexane/2-propanol = 90/10; flow rate 1.0 mL/min; 25 °C; 254 nm; retention time: 9.5 min (minor) and 11.0 min (major).

*(−)-(S,E)-6,6,6-Trifluoro-5-hydroxy-5-(naphthalen-2-yl)-1-phenylhex-2-en-1-one* (**5k**): White solid, Mp 163.1–164.9 °C; 25.9 mg (0.1 mmol), 70% yield; 91% *ee*; ${\left[\alpha \right]}_{D}^{26}$ −40.1 (*c* 0.87, CHCl_3_); ^1^H-NMR (300 MHz, acetone-*d*_6_) δ 8.30 (s, 1H), 8.00–7.92 (m, 3H), 7.83 (d, *J* = 8.8 Hz, 1H), 7.72 (d, *J* = 7.9 Hz, 2H), 7.58–7.51 (m, 3H), 7.36 (t, *J* = 7.7 Hz, 2H), 7.15 (d, *J* = 15.5 Hz, 1H), 6.76–6.67 (m, 1H), 6.13 (s, 1H), 3.63–3.56 (m, 1H), 3.31–3.24 (m, 1H); ^13^C-NMR (75 MHz, acetone-*d*_6_) δ 190.1, 141.7, 138.4, 135.2, 134.0, 133.9, 133.6, 131.1, 129.4, 129.3, 129.2, 128.8, 128.4, 127.8, 127.6, 127.3, 126.9 (q, *J* = 284.2 Hz), 77.5 (q, *J* = 27.5 Hz), 38.8; ^19^F-NMR (376 MHz, CDCl_3_) δ −79.23; HRMS (ESI) *m*/*z* 371.1257 [M + H^+^], calc. for C_22_H_18_F_3_O_2_ 371.1259. The *ee* was determined by HPLC analysis. CHIRALPAK IE (4.6 mm i.d. × 250 mm); Hexane/2-propanol = 95/05; flow rate 1.0 mL/min; 25 °C; 254 nm; retention time: 11.8 min (minor) and 12.7 min (major).

*(−)-(R,E)-6,6,6-Trifluoro-5-hydroxy-1-phenyl-5-(thiophen-2-yl)hex-2-en-1-one* (**5l**): White solid, Mp 77.2–82.2 °C; 27.1 mg (0.1 mmol), 83% yield; 92% *ee*; ${\left[\alpha \right]}_{D}^{26}$ −26.6 (*c* 2.26, CHCl_3_); ^1^H-NMR (300 MHz, CDCl_3_) δ 7.81 (d, *J* = 7.3 Hz, 2H), 7.55 (t, *J* = 7.4 Hz, 1H), 7.42 (t, *J* = 7.5 Hz, 2H), 7.36 (d, *J* = 5.1 Hz, 1H), 7.16 (d, *J* = 3.4 Hz, 1H), 7.04 (dd, *J* = 4.9, 3.9 Hz, 1H), 6.94 (d, *J* = 15.5 Hz, 1H), 6.87–6.78 (m, 1H), 3.58 (s, 1H), 3.13 (s, 1H), 3.10 (s, 1H); ^13^C-NMR (75 MHz, CDCl_3_) δ 190.4, 140.2, 140.0, 137.1, 133.1, 130.8, 128.7, 128.6, 127.3, 126.6, 126.1, 124.6 (q, *J* = 283.9 Hz), 76.4 (q, *J* = 29.7 Hz), 40.0; ^19^F-NMR (376 MHz, CDCl_3_) δ −80.58; HRMS (ESI) *m*/*z* 349.0493 [M + Na^+^], calc. for C_16_H_13_F_3_O_2_SNa 349.0486. The *ee* was determined by HPLC analysis. CHIRALPAK IB-3 (4.6 mm i.d. × 250 mm); Hexane/2-propanol = 90/10; flow rate 1.0 mL/min; 25 °C; 254 nm; retention time: 10.4 min (major) and 11.8 min (minor).

*(+)-(R,E)-5-Hydroxy-1-phenyl-5-(trifluoromethyl)hept-2-en-1-one* (**5m**): Colorless oil; 16.3 mg (0.1 mmol), 60% yield; 91% *ee*; ${\left[\alpha \right]}_{D}^{26}$ +12.4 (*c* 0.74, CHCl_3_); ^1^H-NMR (300 MHz, CDCl_3_) δ 7.94–7.92 (m, 2H), 7.58 (t, *J* = 7.3 Hz, 1H), 7.48 (t, *J* = 7.5 Hz, 2H), 7.08–6.93 (m, 2H), 2.72 (d, *J* = 5.07 Hz, 2H), 2.36 (s, 1H), 1.81 (q, *J* = 7.6 Hz, 2H), 1.04 (t, *J* = 7.6 Hz, 3H); ^13^C-NMR (75 MHz, CDCl_3_) δ 190.2, 141.2, 137.4, 133.0, 129.8, 128.6 (two peaks), 126.2 (q, *J* = 284.9 Hz), 75.4 (q, *J* = 26.9 Hz), 36.5, 27.0, 7.2; ^19^F-NMR (376 MHz, CDCl_3_) δ −79.51; HRMS (ESI) *m*/*z* 273.1103 [M + H^+^], calc. for C_14_H_16_F_3_O_2_ 273.1102. The *ee* was determined by HPLC analysis. CHIRALPAK IB (4.6 mm i.d. × 250 mm); Hexane/2-propanol = 90/10; flow rate 1.0 mL/min; 25 °C; 254 nm; retention time: 7.0 min (major) and 7.7 min (minor).

*(−)-(S,E)-6,6,6-Trifluoro-1-(4-fluorophenyl)-5-hydroxy-5-phenylhex-2-en-1-one* (**5n**): White solid, Mp 106.7–108.0 °C; 27.7 mg (0.1 mmol), 82% yield; 95% *ee*; ${\left[\alpha \right]}_{D}^{26}$ −30.2 (*c* 2.30, CHCl_3_); ^1^H-NMR (300 MHz, CDCl_3_) δ 7.79–7.72 (m, 2H), 7.57 (d, *J* = 7.0 Hz, 2H), 7.45–7.36 (m, 3H), 7.10–7.03 (m, 2H), 6.86 (d, *J* = 15.5 Hz, 1H), 6.75–6.65 (m, 1H), 3.27 (s, 1H), 3.25–3.17 (m, 1H), 3.12–3.04 (m, 1H); ^13^C-NMR (75 MHz, CDCl_3_) δ 188.89, 167.4, 164.0, 140.9, 135.9, 133.5, 133.4, 131.3, 131.2, 130.3, 128.9, 128.6, 126.3, 125.2 (q, *J* = 284.1 Hz), 115.8, 115.5, 76.7 (q, *J* = 28.1 Hz), 39.0; ^19^F-NMR (376 MHz, CDCl_3_) δ −79.56, −105.09; HRMS (ESI) *m*/*z* 339.1015 [M + H^+^], calc. for C_18_H_15_F_4_O_2_ 339.1008. The *ee* was determined by HPLC analysis. CHIRALPAK IB (4.6 mm i.d. × 250 mm); Hexane/2-propanol = 90/10; flow rate 1.0 mL/min; 25 °C; 254 nm; retention time: 8.0 min (minor) and 9.9 min (major).

*(−)-(S,E)-1-(4-Chlorophenyl)-6,6,6-trifluoro-5-hydroxy-5-phenylhex-2-en-1-one* (**5o**): White solid, Mp 90.1–91.5 °C; 30.5 mg (0.1 mmol), 86% yield; 96% *ee*; ${\left[\alpha \right]}_{D}^{26}$ −37.2 (*c* 2.60, CHCl_3_); ^1^H-NMR (300 MHz, CDCl_3_) δ 7.67–7.63 (m, 2H), 7.57 (d, *J* = 6.8 Hz, 2H), 7.47–7.34 (m, 5H), 6.83 (d, *J* = 15.5 Hz, 1H), 6.75–6.65 (m, 1H), 3.34 (s, 1H), 3.24–3.17 (m, 1H), 3.12–3.04 (m, 1H); ^13^C-NMR (75 MHz, CDCl_3_) δ 189.3, 141.3, 139.5, 135.8, 135.4, 130.2, 130.0, 128.9, 128.8, 128.6, 126.30 (two peaks), 125.2 (q, *J* = 284.2 Hz), 76.7 (q, *J* = 28.2 Hz), 39.0; HRMS (ESI) *m*/*z* 355.0720[M + H^+^], calc. for C_18_H_15_ClF_3_O_2_ 355.0713. The *ee* was determined by HPLC analysis. CHIRALPAK IB (4.6 mm i.d. × 250 mm); Hexane/2-propanol = 90/10; flow rate 1.0 mL/min; 25 °C; 254 nm; retention time: 11.0 min (minor) and 16.7 min (major).

*(−)-(S,E)-6,6,6-Trifluoro-1-(3-fluorophenyl)-5-hydroxy-5-phenylhex-2-en-1-one* (**5p**): White solid, Mp 87.1–88.9 °C; 31.1 mg (0.1 mmol), 92% yield; 96% *ee*; ${\left[\alpha \right]}_{D}^{26}$ −27.5 (*c* 1.32, CHCl_3_); ^1^H-NMR (300 MHz, CDCl_3_) δ 7.58–7.50 (m, 3H), 7.46–7.35 (m, 5H), 7.23–7.20 (m, 1H), 6.84 (d, *J* = 15.6 Hz, 1H), 6.77–6.70 (m, 1H), 3.22 (m, 1H), 3.08 (m, 1H), 2.87 (s, 1H); ^13^C-NMR (75 MHz, CDCl_3_) δ 189.0, 164.4, 161.1, 141.3, 139.4, 139.3, 135.8, 130.4, 130.3, 130.2, 129.0, 128.7, 126.3, 125.2 (q, *J* = 283.9 Hz), 124.3 (two peaks), 120.2, 119.9, 115.6, 115.3, 39.1; HRMS (ESI) *m*/*z* 339.1010 [M + H^+^], calc. for C_18_H_15_F_4_O_2_ 339.1008. The *ee* was determined by HPLC analysis. CHIRALPAK IB (4.6 mm i.d. × 250 mm); Hexane/2-propanol = 90/10; flow rate 1.0 mL/min; 25 °C; 254 nm; retention time: 7.6 min (minor) and 8.6 min (major).

*(−)-(S,E)-1-(3-Chlorophenyl)-6,6,6-trifluoro-5-hydroxy-5-phenylhex-2-en-1-one* (**5q**): Colorless oil; 28.4 mg (0.1 mmol), 80% yield; 95% *ee*; ${\left[\alpha \right]}_{D}^{26}$ −38.2 (*c* 1.82, CHCl_3_); ^1^H-NMR (300 MHz, CDCl_3_) δ 7.70 (t, *J* = 1.8 Hz, 1H), 7.59 (t, *J* = 8.2 Hz, 3H), 7.51–7.31 (m, 5H), 6.83 (d, *J* = 15.6 Hz, 1H), 6.76–6.66 (m, 1H), 3.25–3.18 (m, 1H), 3.12–3.05 (m, 2H); ^13^C-NMR (75 MHz, CDCl_3_) δ 189.0, 141.5, 138.7, 135.7, 134.8, 132.9, 130.3, 129.8, 129.0, 128.6, 126.6, 126.2 (two peaks), 125.2 (q, *J* = 284.0 Hz), 76.2, 39.0; HRMS (ESI) *m*/*z* 355.0703 [M + H^+^], calc. for C_18_H_15_ClF_3_O_2_ 355.0713. The *ee* was determined by HPLC analysis. CHIRALPAK IB (4.6 mm i.d. × 250 mm); Hexane/2-propanol = 90/10; flow rate 1.0 mL/min; 25 °C; 254 nm; retention time: 7.7 min (minor) and 8.8 min (major).

*(−)-(S,E)-6,6,6-Trifluoro-1-(2-fluorophenyl)-5-hydroxy-5-phenylhex-2-en-1-one* (**5r**): White solid, Mp 74.0–75.9 °C; 22.0 mg (0.1 mmol), 65% yield; 88% *ee*; ${\left[\alpha \right]}_{D}^{26}$ −40.5 (*c* 1.86, CHCl_3_); ^1^H-NMR (300 MHz, CDCl_3_) δ 7.64–7.55 (m, 3H), 7.52–7.35 (m, 4H), 7.20–7.05 (m, 2H), 6.83 (dd, *J* = 15.5, 3.0 Hz, 1H), 6.73–6.64 (m, 1H), 3.23–3.16 (m, 1H), 3.07–3.03 (m, 1H), 2.96 (s, 1H); ^13^C-NMR (75 MHz, CDCl_3_) δ 188.4, 162.9, 159.5, 140.6, 135.8, 134.3, 134.2, 134.0, 133.9, 131.0, 130.9, 128.9, 128.5, 126.3 (two peaks), 125.1 (q, *J* = 284.2 Hz), 124.5, 124.4, 116.6, 116.3, 76.6 (q, *J* = 28.3 Hz), 38.9; HRMS (ESI) *m*/*z* 339.1009 [M + H^+^], calc. for C_18_H_15_F_4_O_2_ 339.1008. The *ee* was determined by HPLC analysis. CHIRALPAK IA (4.6 mm i.d. × 250 mm); Hexane/2-propanol = 95/05; flow rate 1.0 mL/min; 25 °C; 254 nm; retention time: 9.3 min (major) and 11.1 min (minor).

*(−)-(S,E)-6,6,6-Trifluoro-5-hydroxy-5-phenyl-1-(p-tolyl)hex-2-en-1-one* (**5s**): White solid, Mp 106.2–107.7 °C; 30.8 mg (0.1 mmol), 92% yield; 95% *ee*; ${\left[\alpha \right]}_{D}^{26}$ −33.2 (*c* 1.69, CHCl_3_); ^1^H-NMR (300 MHz, CDCl_3_) δ 7.67 (d, *J* = 8.2 Hz, 2H), 7.58 (d, *J* = 7.2 Hz, 2H), 7.45–7.38 (m, 3H), 7.20 (d, *J* = 8.0 Hz, 2H), 6.74–6.64 (m, 1H), 6.78–6.61 (m, 1H), 3.24–3.04 (m, 3H), 2.39 (s, 3H); ^13^C-NMR (75 MHz, CDCl_3_) δ 189.8, 143.9, 140.0, 136.0, 134.6, 130.8, 129.2, 128.8 (two peaks), 126.3 (two peaks), 125.2 (q, *J* = 284.1 Hz), 76.7, 39.0, 21.6; HRMS (ESI) *m*/*z* 335.1261 [(M + H^+^], calc. for C_19_H_18_F_3_O_2_ 335.1259. The *ee* was determined by HPLC analysis. CHIRALPAK IB (4.6 mm i.d. × 250 mm); Hexane/2-propanol = 90/10; flow rate 1.0 mL/min; 25 °C; 254 nm; retention time: 13.6 min (minor) and 19.3 min (major).

*(−)-(S,E)-6,6,6-Trifluoro-5-hydroxy-5-phenyl-1-(m-tolyl)hex-2-en-1-one* (**5t**): Colorless oil; 30.4 mg (0.1 mmol), 91% yield; 94% *ee*; ${\left[\alpha \right]}_{D}^{26}$ −40.7 (*c* 2.46, CHCl_3_); ^1^H-NMR (300 MHz, CDCl_3_) δ 7.59–7.53 (m, 4H), 7.45–7.28 (m, 5H), 6.89 (d, *J* = 15.5 Hz, 1H), 6.74–6.64 (m, 1H), 3.25–3.18 (m, 1H), 3.15 (s, 1H), 3.12–3.04 (m, 1H), 2.36 (s, 3H); ^13^C-NMR (75 MHz, CDCl_3_) δ 190.6, 140.2, 138.4, 137.2, 135.9, 133.8, 131.0, 130.9, 129.2, 128.8, 128.6, 128.4, 126.3 (two peaks), 125.8, 125.2 (q, *J* = 284.0 Hz), 76.7 (q, *J* = 28.2 Hz), 39.0, 21.3; HRMS (ESI) *m*/*z* 335.1257 [M + H^+^], calc. for C_19_H_18_F_3_O_2_ 335.1259. The *ee* was determined by HPLC analysis. CHIRALPAK IB (4.6 mm i.d. × 250 mm); Hexane/2-propanol = 90/10; flow rate 1.0 mL/min; 25 °C; 254 nm; retention time: 10.5 min (minor) and 11.9 min (major).

*(−)-(S,E)-6,6,6-Trifluoro-5-hydroxy-1-(4-methoxyphenyl)-5-phenylhex-2-en-1-one* (**5u**): White solid, Mp 107.7–109.2 °C; 25.9 mg (0.1 mmol), 74% yield; 95% *ee*; ${\left[\alpha \right]}_{D}^{26}$ −39.6 (*c* 2.20, CHCl_3_); ^1^H-NMR (300 MHz, CDCl_3_) δ 7.77 (d, *J* = 8.8 Hz, 2H), 7.58 (d, *J* = 7.0 Hz, 2H), 7.43–7.35 (m, 3H), 6.93–6.86 (m, 3H), 6.73–6.63 (m, 1H), 3.85 (s, 3H), 3.33 (s, 1H), 3.23–3.16 (m, 1H), 3.11–3.04 (m, 1H); ^13^C-NMR (75 MHz, CDCl_3_) δ 188.6, 163.6, 139.4, 136.0, 131.0, 130.6, 130.0, 128.8, 128.5, 127.1, 126.4 (two, peaks), 125.2 (q, *J* = 284.0 Hz), 113.8, 76.7 (q, *J* = 28.1 Hz), 55.4, 38.9; HRMS (ESI) *m*/*z* 351.1212 [M + H^+^], calc. for C_19_H_18_F_3_O_2_ 351.1208. The *ee* was determined by HPLC analysis. CHIRALPAK IB (4.6 mm i.d. × 250 mm); Hexane/2-propanol = 80/20; flow rate 1.0 mL/min; 25 °C; 254 nm; retention time: 12.1 min (minor) and 15.0 min (major).

*(−)-(S,E)-6,6,6-Trifluoro-5-hydroxy-1-(3-methoxyphenyl)-5-phenylhex-2-en-1-one* (**5v**): Colorless oil; 27.7 mg (0.1 mmol), 79% yield; 94% *ee*; ${\left[\alpha \right]}_{D}^{26}$ −36.3 (*c* 2.76, CHCl_3_); ^1^H-NMR (300 MHz, CDCl_3_) δ 7.57 (d, *J* = 7.1 Hz, 2H), 7.45–7.38 (m, 3H), 7.33–7.31 (m, 3H), 7.11–7.06 (m, 1H), 6.89 (d, *J* = 15.5 Hz, 1H), 6.77–6.67 (m, 1H), 3.81 (s, 3H), 3.25–3.18 (m, 1H), 3.12–3.04 (m, 2H); ^13^C-NMR (75 MHz, CDCl_3_) δ 190.0, 159.8, 140.5, 138.6, 135.9, 130.8, 129.5, 128.9, 128.6, 126.3, 125.2 (q, *J* = 284.2 Hz), 121.3, 119.7, 112.8, 76.7 (q, *J* = 28.2 Hz), 55.4, 39.0; HRMS (ESI) *m*/*z* 351.1211 [M + H^+^], calc. for C_19_H_18_F_3_O_3_ 351.1208. The *ee* was determined by HPLC analysis. CHIRALPAK IB (4.6 mm i.d. × 250 mm); Hexane/2-propanol = 80/20; flow rate 1.0 mL/min; 25 °C; 254 nm; retention time: 17.7 min (minor) and 19.8 min (major).

*(−)-(S,E)-6,6,6-Trifluoro-5-hydroxy-1-(2-methoxyphenyl)-5-phenylhex-2-en-1-one* (**5w**): Colorless oil; 15.4 mg (0.1 mmol), 44% yield; 73% *ee*; ${\left[\alpha \right]}_{D}^{26}$ −38.3 (*c* 1.17, CHCl_3_); ^1^H-NMR (300 MHz, CDCl_3_) δ 7.57 (d, *J* = 7.0 Hz, 2H), 7.48–7.34 (m, 5H), 7.00–6.91 (m, 2H), 6.83 (d, *J* = 15.6 Hz, 1H), 6.59–6.48 (m, 1H), 3.83 (s, 3H), 3.18–3.11 (m, 1H), 3.08–3.01 (m, 1H), 2.93 (s, 1H); ^13^C-NMR (75 MHz, CDCl_3_) δ 192.0, 158.1, 137.8, 136.2, 136.1, 133.3, 130.5, 128.8, 128.5, 128.2, 126.3, 125.1 (q, *J* = 283.9 Hz), 120.8, 111.5, 76.4 (q, *J* = 28.1 Hz), 55.7, 38.8; HRMS (ESI) *m*/*z* 351.1204 [M + H^+^], calc. for C_19_H_18_F_3_O_3_ 351.1208. The *ee* was determined by HPLC analysis. CHIRALPAK IB (4.6 mm i.d. × 250 mm); Hexane/2-propanol = 95/05; flow rate 1.0 mL/min; 25 °C; 254 nm; retention time: 19.3 min (minor) and 22.1 min (major).

*(−)-(S,E)-6,6,6-Trifluoro-5-hydroxy-1-(naphthalen-2-yl)-5-phenylhex-2-en-1-one* (**5x**): White solid, Mp 91.4–93.2 °C; 29.6 mg (0.1 mmol), 80% yield; 93% *ee*; ${\left[\alpha \right]}_{D}^{26}$ −28.0 (*c* 3.13, CHCl_3_); ^1^H-NMR (300 MHz, CDCl_3_) δ 8.20 (s, 1H), 7.87–7.80 (m, 4H), 7.63–7.37 (m, 7H), 7.04 (d, *J* = 15.5 Hz, 1H), 6.83–6.73 (m, 1H), 3.43 (d, *J* = 3.3 Hz, 1H), 3.30–3.22 (m, 1H), 3.17–3.10 (m, 1H); ^13^C-NMR (75 MHz, CDCl_3_) δ 190.3, 140.5, 136.0, 135.5, 134.4, 132.3, 130.8, 130.4, 129.5, 128.9, 128.6, 128.5 (two peaks), 126.7, 126.4 (two peaks), 125.2 (q, *J* = 283.9 Hz), 76.7 (q, *J* = 28.1 Hz), 38.9; HRMS (ESI) *m*/*z* 371.1258 [M + H^+^], calc. for C_22_H_18_F_3_O_2_ 371.1259. The *ee* was determined by HPLC analysis. CHIRALPAK IB (4.6 mm i.d. × 250 mm); Hexane/2-propanol = 80/20; flow rate 1.0 mL/min; 25 °C; 254 nm; retention time: 12.5 min (minor) and 20.9 min (major).

*(−)-(S,E)-6,6,6-Trifluoro-5-hydroxy-5-phenyl-1-(thiophen-2-yl)hex-2-en-1-one* (**5y**): White solid, Mp 126.3–128.0 °C; 27.1 mg (0.1 mmol), 83% yield; 98% *ee*; ${\left[\alpha \right]}_{D}^{26}$ −44.6 (*c* 2.46, CHCl_3_); ^1^H-NMR (300 MHz, CDCl_3_) δ 7.64–7.56 (m, 4H), 7.44–7.34 (m, 3H), 7.11–7.08 (m, 1H), 6.84 (d, *J* = 15.3 Hz, 1H), 6.80–6.73 (m, 1H), 3.26–3.18 (m, 2H), 3.11–3.05 (m, 1H); ^13^C-NMR (75 MHz, CDCl_3_) δ 181.7, 144.4, 139.9, 135.9, 134.4, 132.5, 130.1, 128.9, 128.5, 128.2, 126.3, 125.2 (q, *J* = 284.1 Hz), 76.6 (q, *J* = 28.3 Hz), 38.8; HRMS (ESI) *m*/*z* 327.0666 [M + H^+^], calc. for C_16_H_14_F_3_O_2_S 327.0667. The *ee* was determined by HPLC analysis. CHIRALPAK IB (4.6 mm i.d. × 250 mm); Hexane/2-propanol = 90/10; flow rate 1.0 mL/min; 25 °C; 254 nm; retention time: 30.6 min (minor) and 31.5 min (major).

*(+)-(S,E)-Methyl 2-hydroxy-6-oxo-2,6-diphenylhex-4-enoate* (**7a**): Colorless oil; 24.2 mg (0.1 mmol), 78% yield; 89% *ee*; ${\left[\alpha \right]}_{D}^{26}$ +5.8 (*c* 1.27, CHCl_3_); ^1^H-NMR (300 MHz, CDCl_3_) δ 7.85–7.82 (m, 2H), 7.63–7.52 (m, 3H), 7.46–7.30 (m, 5H), 6.92–6.90 (m, 2H), 3.82 (s, 4H), 3.22–3.15 (m, 1H), 3.06–3.00 (m, 1H); ^13^C-NMR (75 MHz, CDCl_3_) δ 190.7, 174.5, 142.6, 140.8, 137.6, 132.7, 129.9, 128.6, 128.5 (two peaks), 128.2, 125.3, 78.0, 53.5, 43.0; HRMS (ESI) *m*/*z* 333.1092 [M + H^+^], calc. for C_19_H_18_O_4_Na 333.1103. The *ee* was determined by HPLC analysis. CHIRALPAK IC (4.6 mm i.d. × 250 mm); Hexane/2-propanol = 80/20; flow rate 1.0 mL/min; 25 °C; 254 nm; retention time: 20.7 min (minor) and 23.6 min (major).

*(−)-(R,E)-Diethyl (1-hydroxy-5-oxo-1,5-diphenylpent-3-en-1-yl)phosphonate* (**9a**): White solid; Mp 130.1‒131.9 °C; 26.0 mg (0.1 mmol), 67% yield; 92% *ee*; ${\left[\alpha \right]}_{D}^{26}$ −16.1 (*c* 1.13, CHCl_3_); ^1^H-NMR (300 MHz, CDCl_3_) δ 7.73–7.70 (m, 2H), 7.76–7.59 (m, 2H), 7.50 (t, *J* = 7.4 Hz, 1H), 7.38 (t, *J* = 7.5 Hz, 4H), 7.31 (dd, *J* = 7.2, 1.6 Hz, 1H), 6.86–6.70 (m, 2H), 4.17–4.07 (m, 2H), 4.04–3.89 (m, 1H), 3.87–3.76 (m, 1H), 3.63 (d, *J* = 6.7 Hz, 1H), 3.21 (t, *J* = 7.1 Hz, 2H), 3.21 (t, *J* = 7.1 Hz, 2H), 1.27 (t, *J* = 7.0 Hz, 3H), 1.17 (t, *J* = 7.1 Hz, 3H); ^13^C-NMR (75 MHz, CDCl_3_) δ 190.8, 142.4, 142.2, 138.1, 137.5, 132.6, 130.3, 128.6, 128.4, 128.3, 128.2, 127.7, 127.6, 126.1 (two peaks), 76.6, 74.5, 63.7, 63.6, 63.5, 63.4, 41.4, 41.3, 16.4, 16.3 (two peaks), 16.2; HRMS (ESI) *m*/*z* 411.1336 [M + Na^+^], calc. for C_21_H_25_O_5_P 411.1337. The *ee* was determined by HPLC analysis. CHIRALPAK IC (4.6 mm i.d. × 250 mm); Hexane/2-propanol = 80/20; flow rate 1.0 mL/min; 25 °C; 254 nm; retention time: 19.2 min (minor) and 22.4 min (major).

## 4. Conclusions

In summary, we have developed an elaborate one-pot tandem strategy, which allowed homoallyic alcohols to be successfully employed in the direct AVA reaction with a series of activated acyclic ketones, such as trifluoromethyl ketones, α-ketoesters, and α-keto phosphonates, in high yields, γ-selectivity, and stereoselectivity. This modular combination, including Jones oxidation and asymmetric hydrogen bonding catalysis, features satisfactory compatibility and reliability, albeit without an intermediary purification step. This methodology also presents effective in large-scale synthesis. We anticipate that this efficient one-pot tandem paradigm will find application in more types of direct vinylogous reactions using homoallylic alcohols as pro-nucleophiles.

## Figures and Tables

**Table 1:**
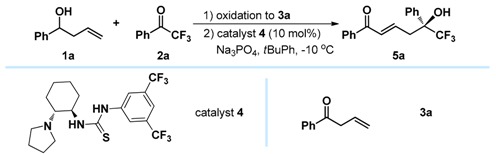
Investigation of reaction conditions ^a^.

Entry	Oxidation Conditions	Conversion of 3a ^b^	Yield of 5a ^c^	*ee* of 5a ^d^
1	DMP (2.0 equiv.), CH_2_Cl_2_, 25 °C, 20 min	>99%	0 (N.R.)	N.D.
2	Jones reagent (1.5 equiv.), CH_2_Cl_2_, 25 °C, 10 min	>99%	0 (N.R.)	N.D.
3	Jones reagent (1.5 equiv.), *t*BuPh, 0 °C, 4.0 h, then aqueous phase released	>99%	80	95

^a^ Reaction conditions: **1a** (0.4 mmol), **2a** (0.1 mmol), 2.0 equiv. of Na_3_PO_4_, 0.8 mL solvent; ^b^ the conversion was determined by the crude ^1^H-NMR; ^c^ yield of isolated product; ^d^ *Ees* were determined by HPLC analysis on a chiral stationary phase. DMP = Dess-Martin Periodinane.

**Table 2:**
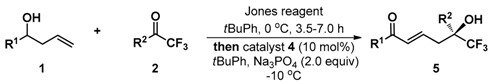
One-pot tandem reactions between homoallylic alcohols **1** and trifluoromethyl ketones **2** ^a^.

Entry	R^1^	R^2^	t (h)	5	Yield (%) ^b^	*ee* (%) ^c^
1	Ph	C_6_H_4_	38	**5a**	80	95
2	Ph	4-CF_3-_C_6_H_4_	39	**5b**	87	92
3	Ph	4-F-C_6_H_4_	39	**5c**	71	94
4	Ph	4-Cl-C_6_H_4_	60	**5d**	98	94
5	Ph	4-Br-C_6_H_4_	41	**5e**	80	95
6	Ph	3-F-C_6_H_4_	38	**5f**	95	91
7	Ph	3-Cl-C_6_H_4_	61	**5g**	94	90
8	Ph	2-F-C_6_H_4_	78	**5h**	71	99 ^d^
9	Ph	4-Me-C_6_H_4_	62	**5i**	60	95
10	Ph	3-Me-C_6_H_4_	60	**5j**	67	94
11	Ph	2-naphthyl	66	**5k**	70	91
12	Ph	2-thienyl	63	**5l**	83	92
13	Ph	Et	80	**5m**	60	91
14	4-FC_6_H_4_	Ph	61	**5n**	82	95
15	4-ClC_6_H_4_	Ph	70	**5o**	86	96
16	3-FC_6_H_4_	Ph	59	**5p**	92	96
17	3-ClC_6_H_4_	Ph	70	**5q**	80	95
18	2-FC_6_H_4_	Ph	64	**5r**	65	88
19	4-MeC_6_H_4_	Ph	64	**5s**	92	95
20	3-MeC_6_H_4_	Ph	61	**5t**	91	94
21	4-MeOC_6_H_4_	Ph	34	**5u**	74	95
22	3-MeOC_6_H_4_	Ph	61	**5v**	79	94
23	2-MeOC_6_H_4_	Ph	64	**5w**	44	73
24	2-napthyl	Ph	70	**5x**	80	93
25	2-thienyl	Ph	65	**5y**	83	98

^a^ Reaction conditions: **1** (0.4 mmol), Jones reagent (1.5 equiv.), 1.0 mL *t*BuPh, 0 °C, then **2** (0.1 mmol), catalyst **IV** (0.01 mmol), Na_3_PO_4_ (0.2 mmol), −10 °C. In all reactions, the ratio of E/Z was >20:1 determined by crude ^1^H-NMR; ^b^ Yield of isolated product; ^c^ determined by HPLC analysis on a chiral stationary phase; ^d^ the *ee* value and yield were obtained after a single recrystallization. Initial data: 80% yield, 85% *ee*.

## Supplementary Materials

Supplementary materials can be accessed at: http://www.mdpi.com/1420-3049/21/7/842/s1.

## References

[1] Chinchilla R., Nájera C. (2000). Acylvinyl and vinylogous synthons. Chem. Rev..

[2] Casiraghi G., Zanardi F. (2000). The vinylogous aldol reaction: A valuable, yet understated carbon-carbon bond-forming maneuver. Chem. Rev..

[3] Casiraghi G., Battistini L., Curti C., Rassu G., Zanardi F. (2011). The vinylogous aldol and related addition reactions: Ten years of progress. Chem. Rev..

[4] Yan L., Wu X., Liu H., Xie L., Jiang Z. (2013). Catalytic asymmetric synthesis of γ-butenolides by direct vinylogous reactions. Mini-Rev. Med. Chem..

[5] Zhang Q., Liu X., Feng X. (2013). Recent advances in enantioselective synthesis of γ-substituted butenolides via the catalytic asymmetric vinylogous reactions. Curr. Org. Synth..

[6] Das Sarma K., Zhang J., Curran T.T. (2007). Novel synthons from mucochloric acid: The first use of α,β-dichloro-γ-butenolides and γ-butyrolactams for direct vinylogous aldol addition. J. Org. Chem..

[7] Ube H., Shimada N., Terada M. (2010). Asymmetric direct vinylogous aldol reaction of furanone derivatives catalyzed by an axially chiral guanidine base. Angew. Chem. Int. Ed..

[8] Yang Y., Zheng K., Zhao J., Shi J., Lin L., Liu X., Feng X. (2010). Asymmetric direct vinylogous aldol reaction of unactivated γ-butenolide to aldehydes. J. Org. Chem..

[9] Pansare S.V., Paul E.K. (2011). Organocatalytic asymmetric direct vinylogous aldol reactions of γ-crotonolactone with aromatic aldehydes. Chem. Commun..

[10] Bastida D., Liu Y., Tian X., Escudero-Adán E., Melchiorre P. (2013). Asymmetric vinylogous aldol reaction via H-bond-directing dienamine catalysis. Org. Lett..

[11] Luo J., Wang H., Han X., Xu L.W., Kwiatkowski J., Huang K.W., Lu Y. (2011). The Direct asymmetric vinylogous aldol reaction of furanones with α-ketoesters: Access to chiral γ-butenolides and glycerol derivatives. Angew. Chem. Int. Ed..

[12] Yazaki R., Kumagai N., Shibasaki M. (2009). Direct catalytic asymmetric addition of allyl cyanide to ketones. J. Am. Chem. Soc..

[13] Yazaki R., Kumagai N., Shibasaki M. (2010). Direct catalytic asymmetric addition of allyl cyanide to ketones via soft Lewis acid/hard Brønsted base/hard Lewis base catalysis. J. Am. Chem. Soc..

[14] Zhu B., Zhang W., Lee R., Han Z., Yang W., Tan D., Jiang Z. (2013). Direct asymmetric vinylogous aldol reaction of allyl ketones with isatins: Divergent synthesis of 3-hydroxy-2-oxindole derivatives. Angew. Chem. Int. Ed..

[15] Li T.Z., Jiang Y., Guan Y.Q., Sha F., Wu X.Y. (2014). Direct enantioselective vinylogous aldol-yclization cascade reaction of allyl pyrazoleamides with isatins: Asymmetric construction of spirocyclic oxindole-dihydropyranones. Chem. Commun..

[16] Jiang L., Lei Q., Huang X., Cui H.L., Zhou X., Chen Y.C. (2011). Lewis base assisted Brønsted base catalysis: Direct regioselective asymmetric vinylogous alkylation of allylic sulfones. Chem. Eur. J..

[17] Zhan G., He Q., Yuan X., Chen Y.C. (2014). Asymmetric direct vinylogous Michael additions of allyl alkyl ketones to maleimides through dienamine catalysis. Org. Lett..

[18] Gu Y., Wang Y., Yu T.Y., Liang Y.M., Xu P.F. (2014). Rationally designed multifunctional supramolecular iminium catalysis: Direct vinylogous Michael addition of unmodified linear dienol substrates. Angew. Chem. Int. Ed..

[19] Jing Z., Bai X., Chen W., Zhang G., Zhu B., Jiang Z. (2016). Organocatalytic enantioselective vinylogous aldol reaction of allyl aryl ketones to activated acyclic ketones. Org. Lett..

[20] Jiang D., Peng J., Chen Y. (2008). Pd-catalyzed carboetherification of β,γ-unsaturated oximes: A novel approach to Δ2-isoxazolines. Org. Lett..

[21] Zhuo L.G., Yao Z.K., Yu Z.X. (2013). Synthesis of Z-alkenes from Rh (I)-catalyzed olefin isomerization of β,γ-unsaturated ketones. Org. Lett..

[22] Qiao B., Huang Y.J., Nie J., Ma J.A. (2015). Highly regio-, diastereo-, and enantioselective Mannich reaction of allylic ketones and cyclic ketimines: Access to chiral benzosultam. Org. Lett..

[23] Enders D., Grondal C., Huettl M.R. (2007). Asymmetric organocatalytic domino reactions. Angew. Chem. Int. Ed..

[24] Walji A.M., MacMillan D.W. (2007). Strategies to bypass the taxol problem. Enantioselective cascade catalysis, a new approach for the efficient construction of molecular complexity. Synlett.

[25] Gaunt M.J., Johansson C.C., McNally A., Vo N.T. (2007). Enantioselective organocatalysis. Drug Discov. Today.

[26] Dondoni A., Massi A. (2008). Asymmetric organocatalysis: From infancy to adolescence. Angew. Chem. Int. Ed..

[27] Albrecht Ł., Jiang H., Jørgensen K.A. (2011). A Simple recipe for sophisticated cocktails: Organocatalytic one-pot reactions-concept, nomenclature, and future perspectives. Angew. Chem. Int. Ed..

[28] Bernardi L., Fochi M., Franchini M.C., Ricci A. (2012). Bioinspired organocatalytic asymmetric reactions. Org. Biomol. Chem..

