# Supplementary material for: A One-Pot Tandem Strategy in Catalytic Asymmetric Vinylogous Aldol Reaction of Homoallylic Alcohols
Source: Molecules. 2016 Jun 27;21(7):842. doi: 10.3390/molecules21070842 (PMC6272830; doi:10.3390/molecules21070842)
Supplement: Supplementary file 1 [file molecules-21-00842-s001.pdf]

Supplementary Materials: A One-Pot Tandem Strategy in Catalytic Asymmetric Vinylogous Aldol Reaction of Homoallylic Alcohols

Xufeng Hou, Zhenzhong Jing, Xiangbin Bai and Zhiyong Jiang

1. General Information

1.1. General Procedures and Methods

Experiments involving moisture and/or air sensitive components were performed under a positive pressure of nitrogen in oven-dried glassware equipped with a rubber septum inlet. Dried solvents and liquid reagents were transferred by oven-dried syringes or hypodermic syringe cooled to ambient temperature in a desiccator. Reactions mixtures were stirred in 10 mL sample vial with Teflon-coated magnetic stirring bars unless otherwise stated. Moisture in non-volatile reagents/compounds was removed in high vacuo by means of an oil pump and subsequent purging with nitrogen. Solvents were removed in vacuo under ~30 mmHg and heated with a water bath at 30–35 °C using Changcheng rotary evaporator with Changcheng aspirator. The condenser was cooled with running water at 0 °C.

All experiments were monitored by analytical thin layer chromatography (TLC). TLC was performed on pre-coated plates, 60 F_{254}. After elution, plate was visualized under UV illumination at 254 nm for UV active material. Further visualization was achieved by staining $KMnO_4$, ceric molybdate, or anisaldehyde solution. For those using the aqueous stains, the TLC plates were heated on a hot plate.

Columns for flash chromatography (FC) contained silica gel 200–300 mesh. Columns were packed as slurry of silica gel in petroleum ether and equilibrated solution using the appropriate solvent system. The elution was assisted by applying pressure of about 2 atm with an air pump.

1.2. Instrumentations

Proton nuclear magnetic resonance (^{1}H-NMR), carbon NMR (^{13}C-NMR), and fluorous (^{19}F-NMR) spectra were recorded in $CDCl_3$ otherwise stated. Chemical shifts are reported in parts per million (ppm), using the residual solvent signal as an internal standard: $CDCl_3$ (^{1}H-NMR: δ 7.26, singlet; ^{13}C-NMR: δ 77.0, triplet). Multiplicities were given as: s (singlet), d (doublet), t (triplet), q (quartet), quintet, m (multiplets), dd (doublet of doublets), dt (doublet of triplets), and br (broad). Coupling constants (*J*) were recorded in Hertz (Hz). The number of proton atoms (*n*) for a given resonance was indicated by *n*H. The number of carbon atoms (*n*) for a given resonance was indicated by *n*C. HRMS was reported in units of mass of charge ratio (*m*/*z*). Mass samples were dissolved in CH_3CN (HPLC Grade) unless otherwise stated. Optical rotations were recorded on a polarimeter with a sodium lamp of wavelength 589 nm and reported as follows; $[\alpha]_{\lambda}^{T\,°C}$ (*c* = g/100 mL, solvent). Melting points were determined on a melting point apparatus.

Enantiomeric excesses were determined by chiral High Performance Liquid Chromatography (HPLC) analysis. UV detection was monitored at 254 nm, 230 nm and 210 nm at the same time. HPLC samples were dissolved in HPLC grade isopropanol (IPA) unless otherwise stated.

1.3. Materials

All commercial reagents were purchased with the highest purity grade. They were used without further purification unless specified. All solvents used, mainly petroleum ether (PE) and ethyl acetate (EtOAc) were distilled. Anhydrous DCM and CH_3CN were freshly distilled from CaH_2 and stored under N_2 atmosphere. THF, Et_2O, *tert*-butylbenzene and toluene were freshly distilled from sodium/benzophenone before use. Anhydrous methanol and etanol were distilled from Mg. All compounds synthesized were stored in a −20 °C freezer and light-sensitive compounds were protected with aluminium foil.

2. Characterization of Adducts

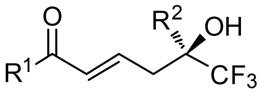

5a: R^1 = Ph
R^2 = Ph

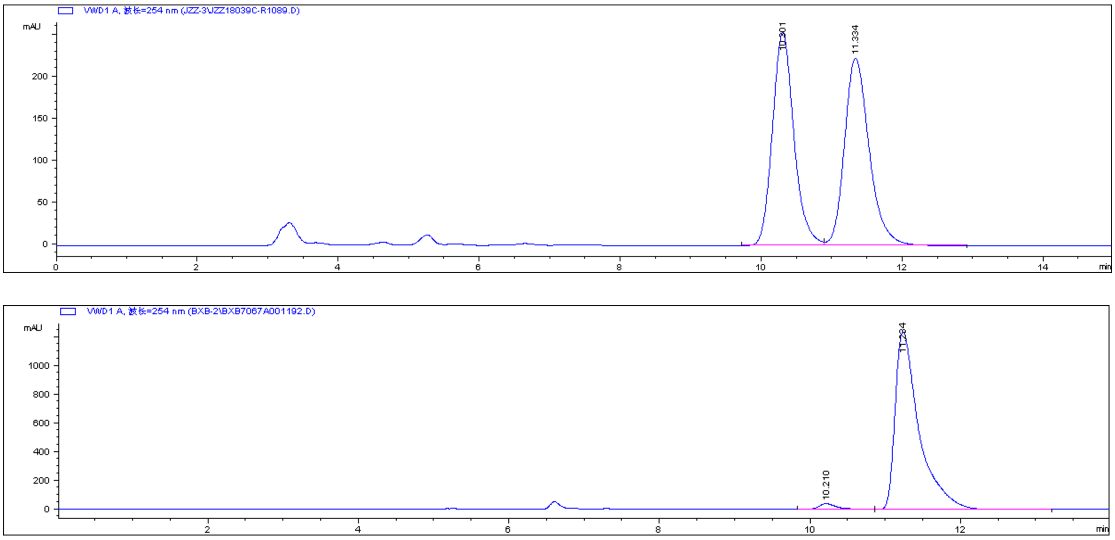

Figure S1. HPLC spectra of **5a**.

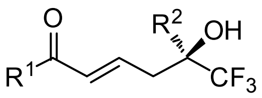

5b: R^1 = Ph
R^2 = 4-CF_3Ph

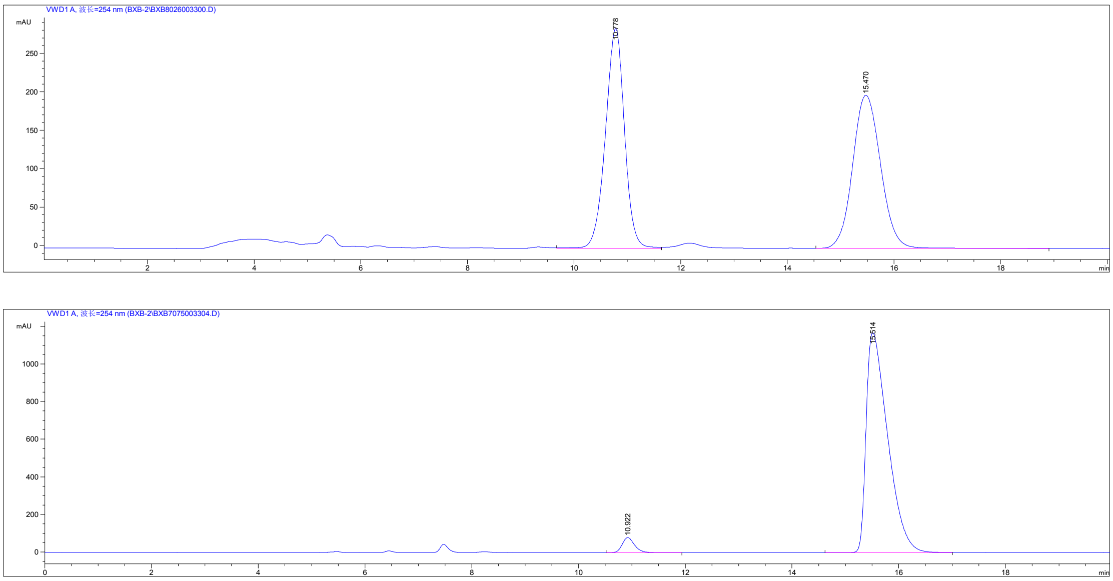

Figure S2. HPLC spectra of **5b**.

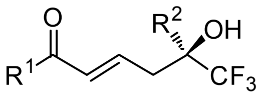

5c: R^1 = Ph
R^2 = 4-FPh

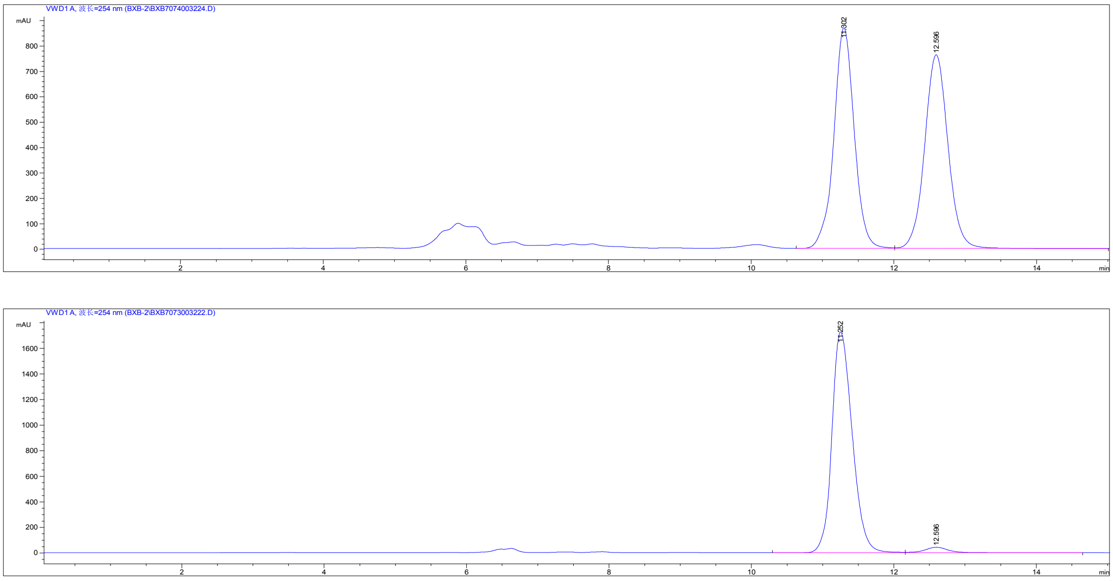

Figure S3. HPLC spectra of **5c.**

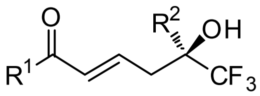

5d: R^1 = Ph
R^2 = 4-ClPh

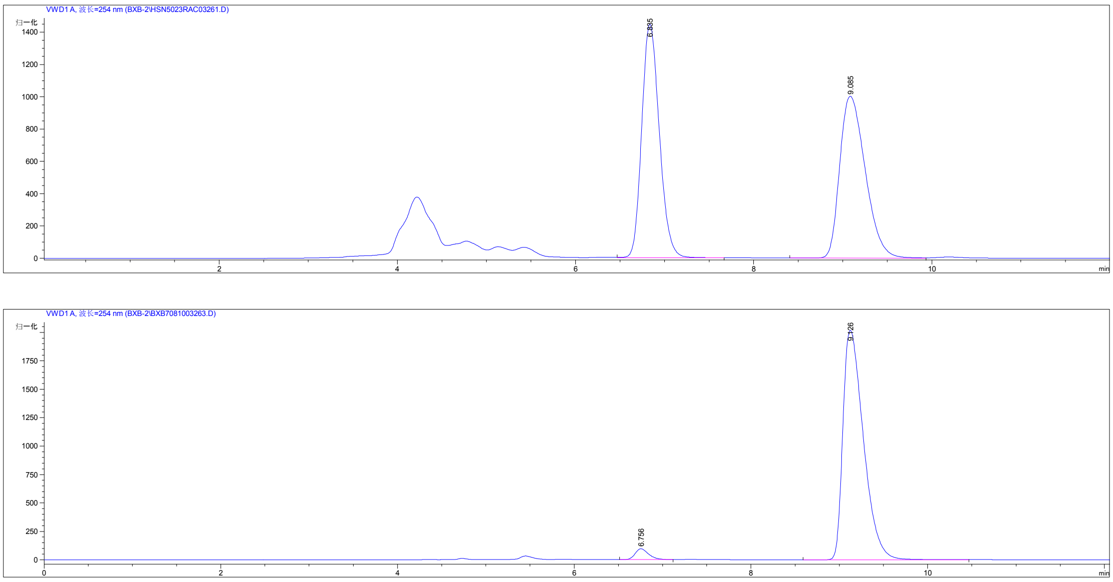

Figure S4. HPLC spectra of **5d.**

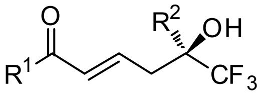

5e: R^1 = Ph
R^2 = 4-BrPh

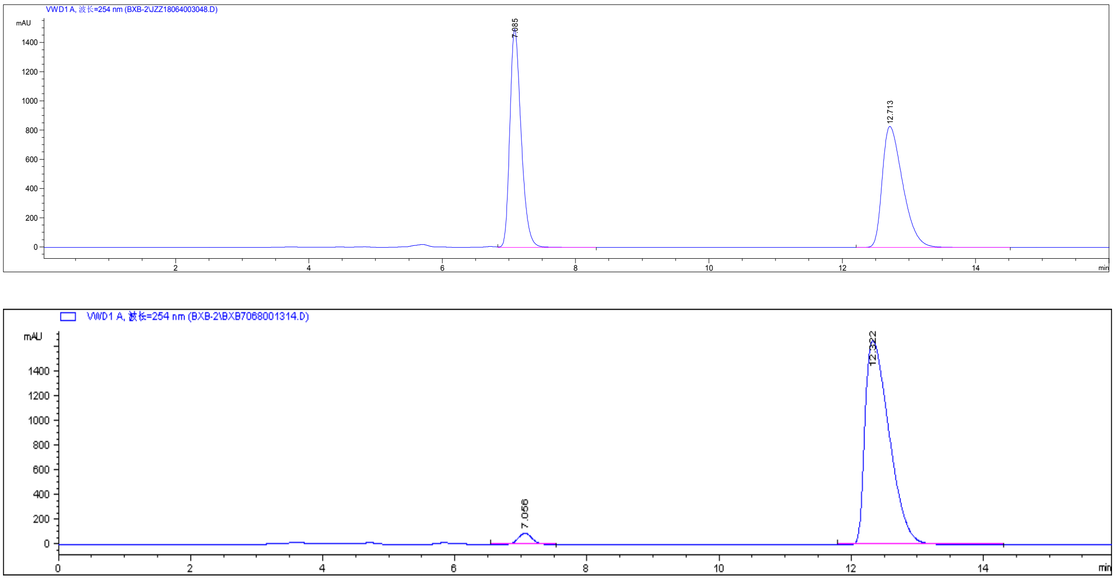

Figure S5. HPLC spectra of **5e.**

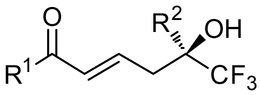

5f: R^1 = Ph
R^2 = 3-FPh

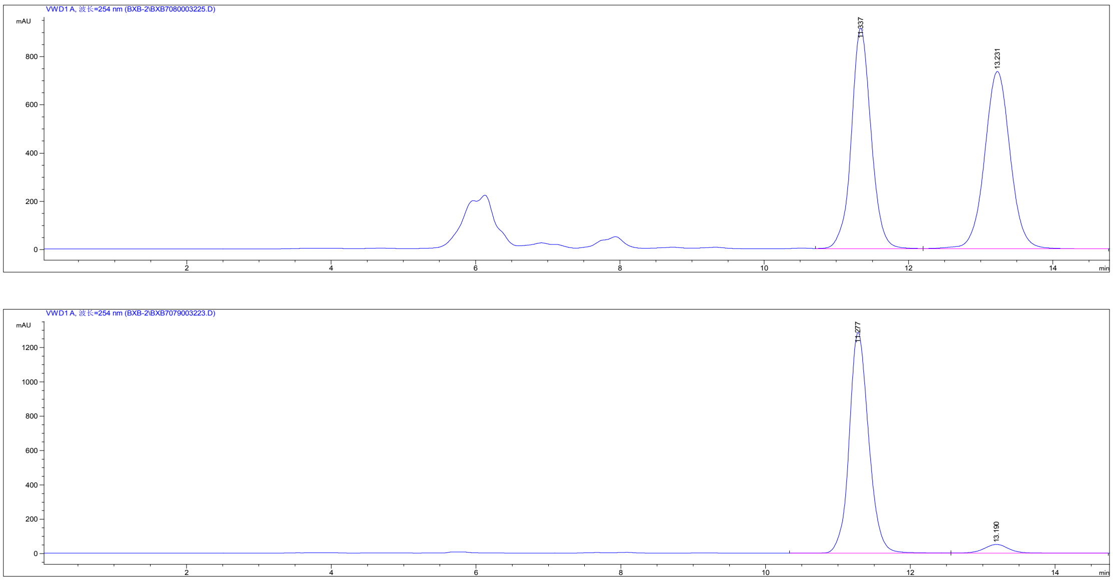

Figure S6. HPLC spectra of **5f.**

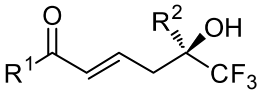

5g: R^1 = Ph
R^2 = 3-ClPh

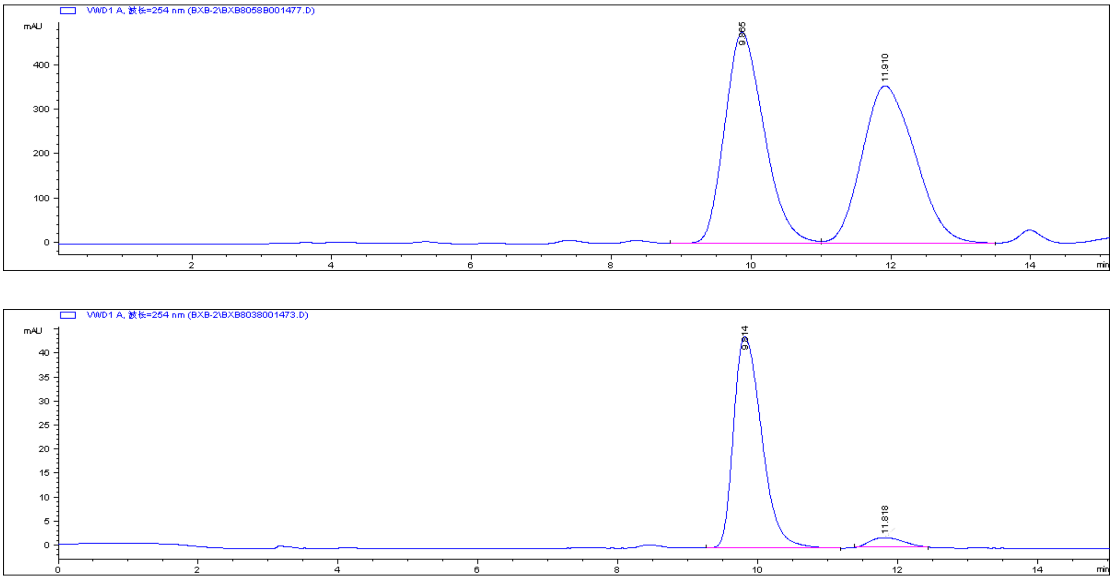

Figure S7. HPLC spectra of **5g.**

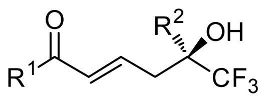

5h: R^1 = Ph
R^2 = 2-FPh

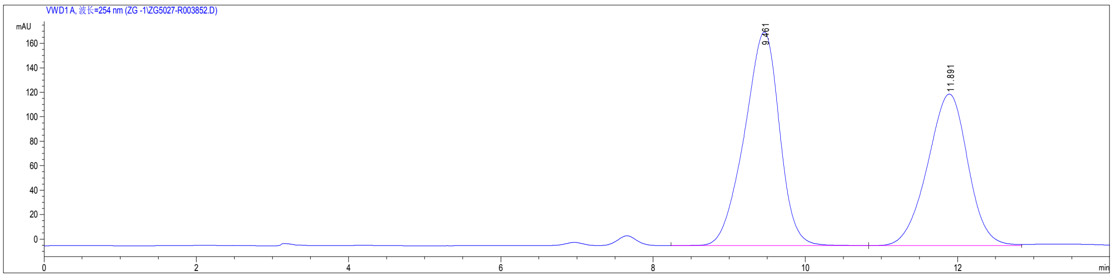

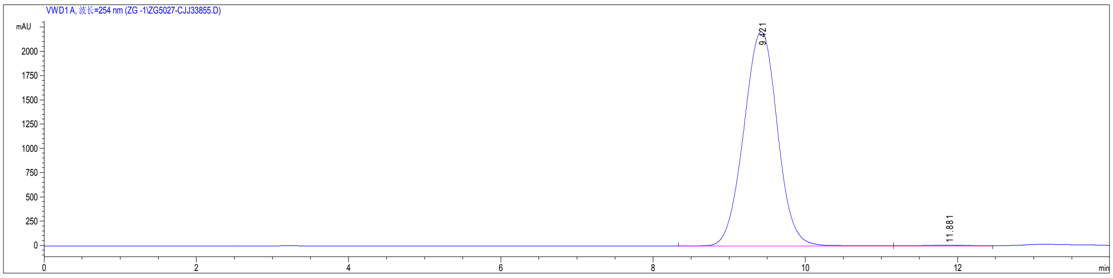

Figure S8. HPLC spectra of **5h.**

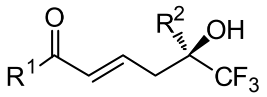

5i: R^1 = Ph
R^2 = 4-MePh

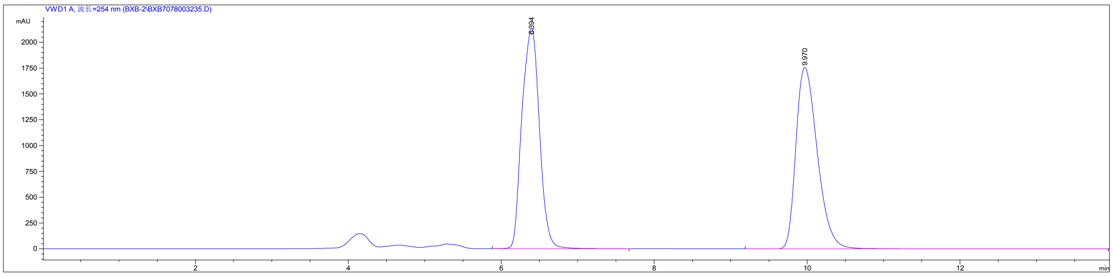

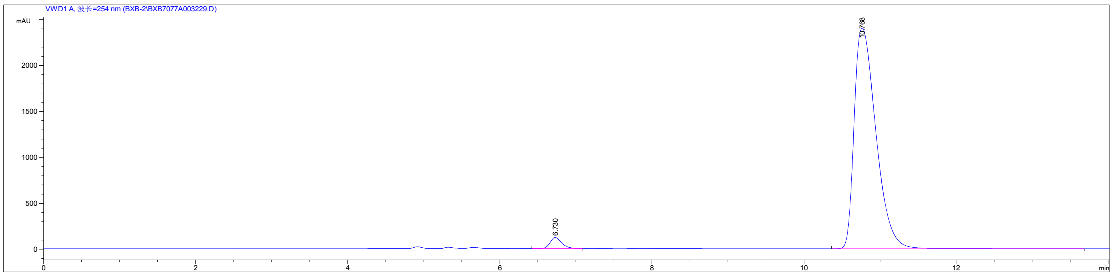

Figure S9. HPLC spectra of **5i.**

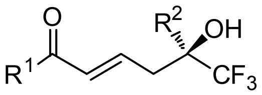

5j: R^1 = Ph
R^2 = 3-MePh

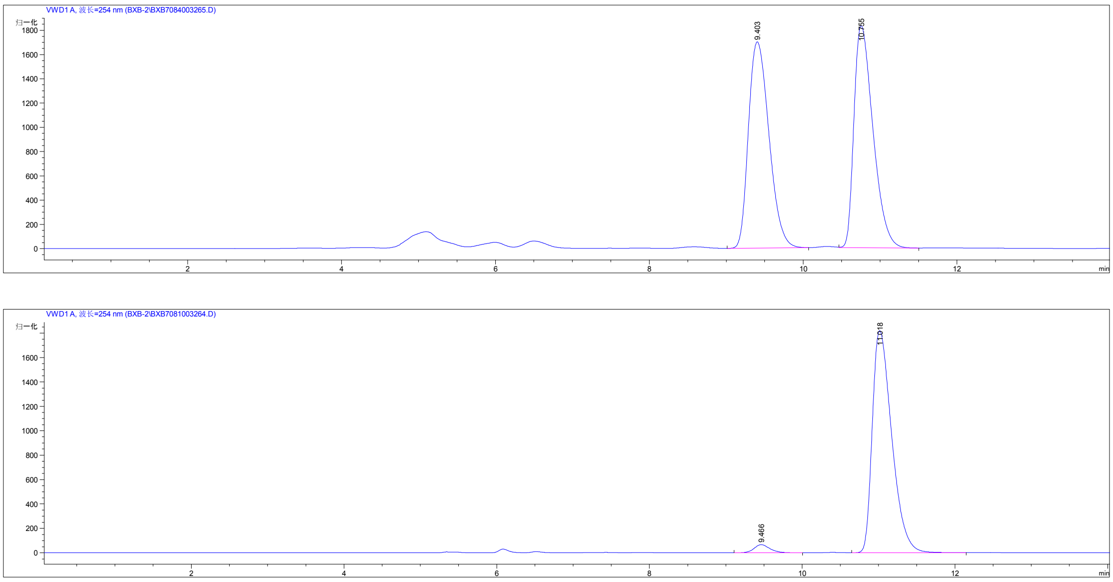

Figure S10. HPLC spectra of **5j.**

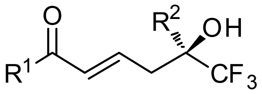

5k: R^1 = Ph
R^2 = 2-naphtyl

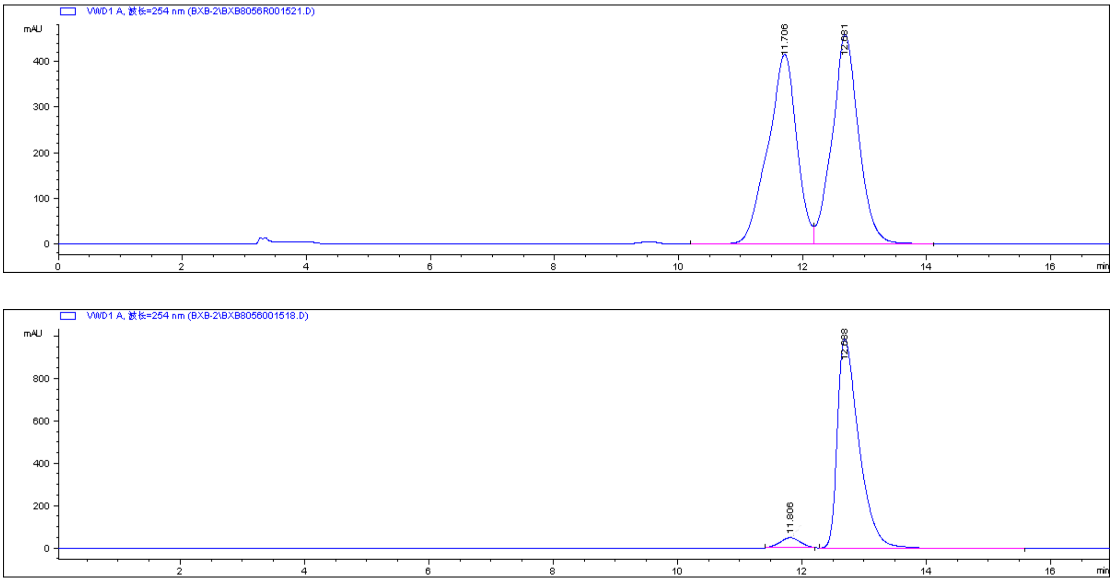

Figure S11. HPLC spectra of **5k.**

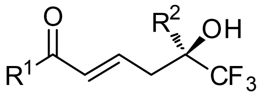

5l: R^1 = Ph
R^2 = 2-thienyl

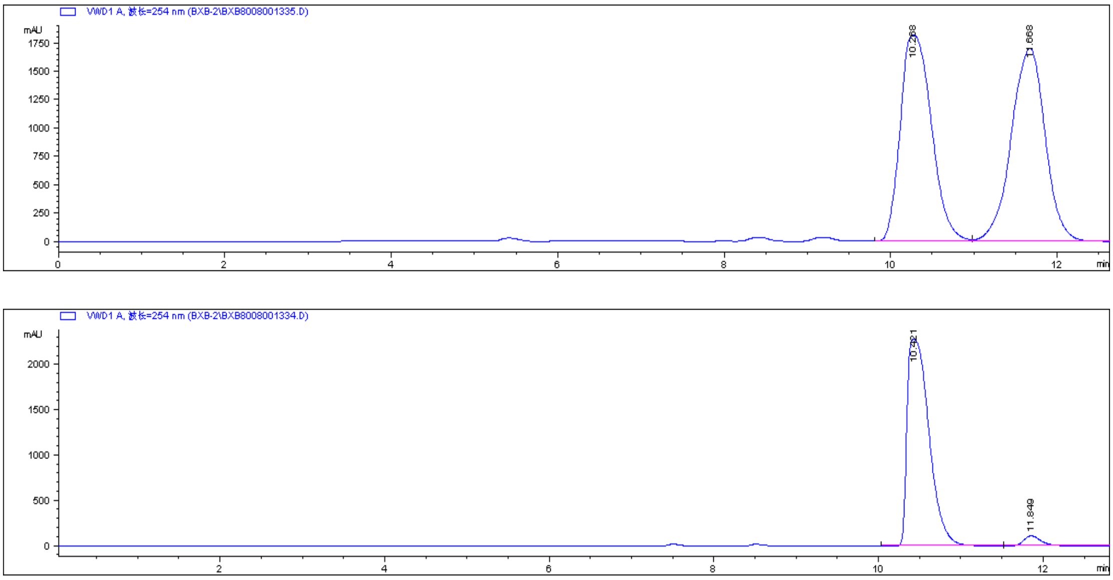

Figure S11. HPLC spectra of **5l.**

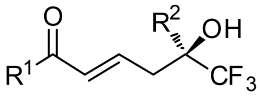

5m: R^1 = Ph
R^2 = Et

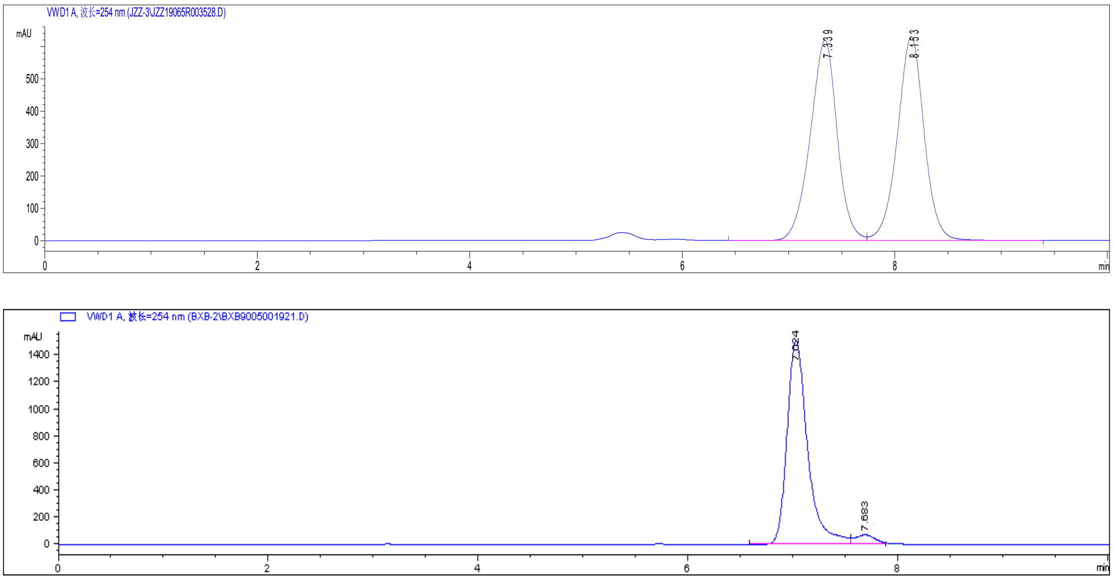

Figure S12. HPLC spectra of **5m.**

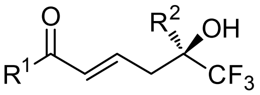

5n: R^1 = 4-FPh
R^2 = Ph

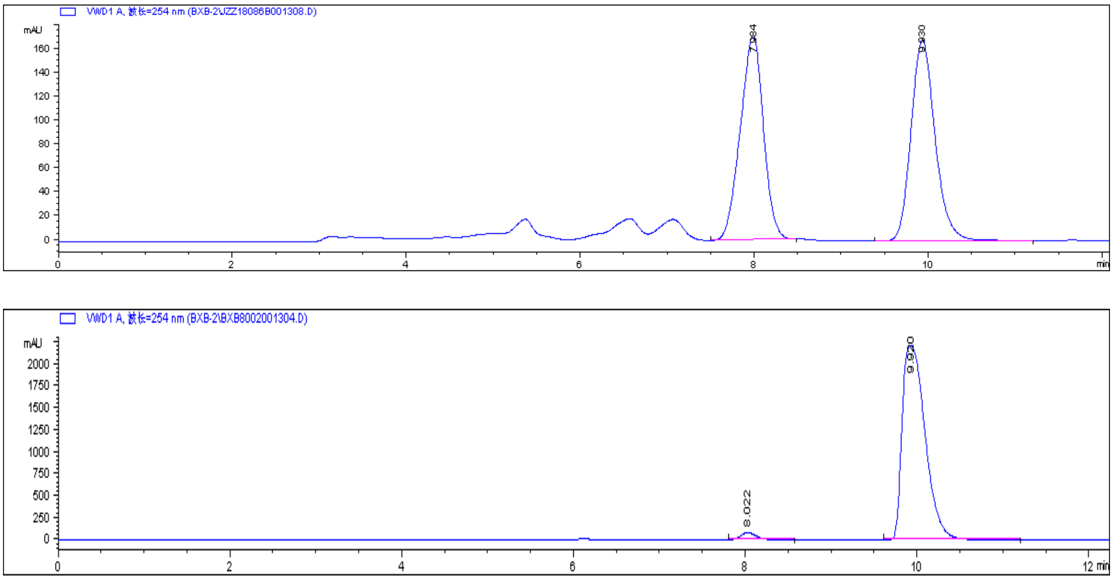

Figure S13. HPLC spectra of **5n.**

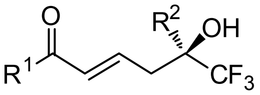

5o: R^1 = 4-ClPh
R^2 = Ph

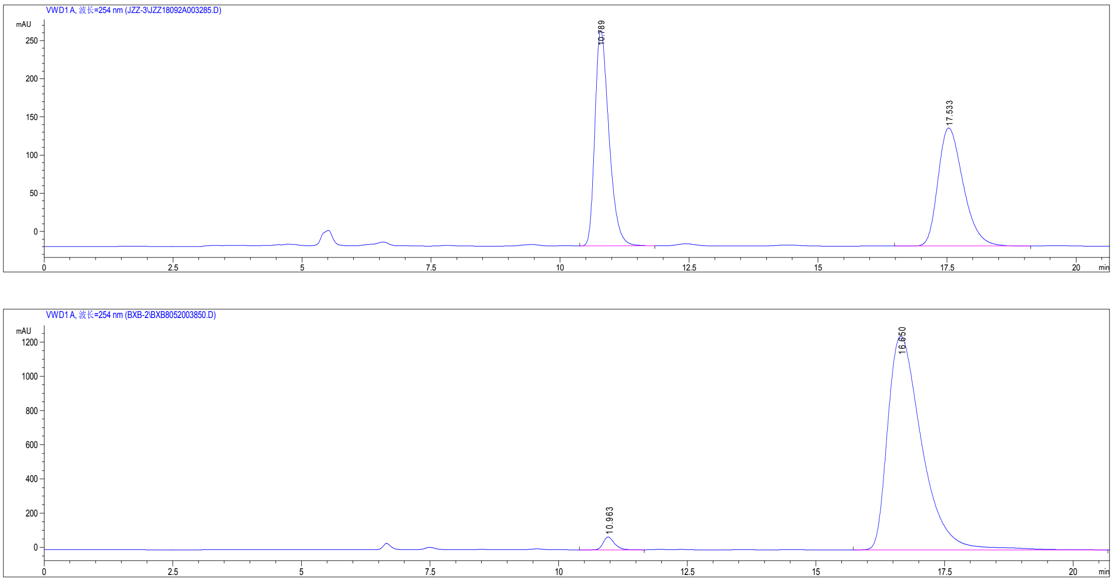

Figure S14. HPLC spectra of **5o.**

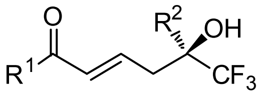

5p: R^1 = 3-FPh
R^2 = Ph

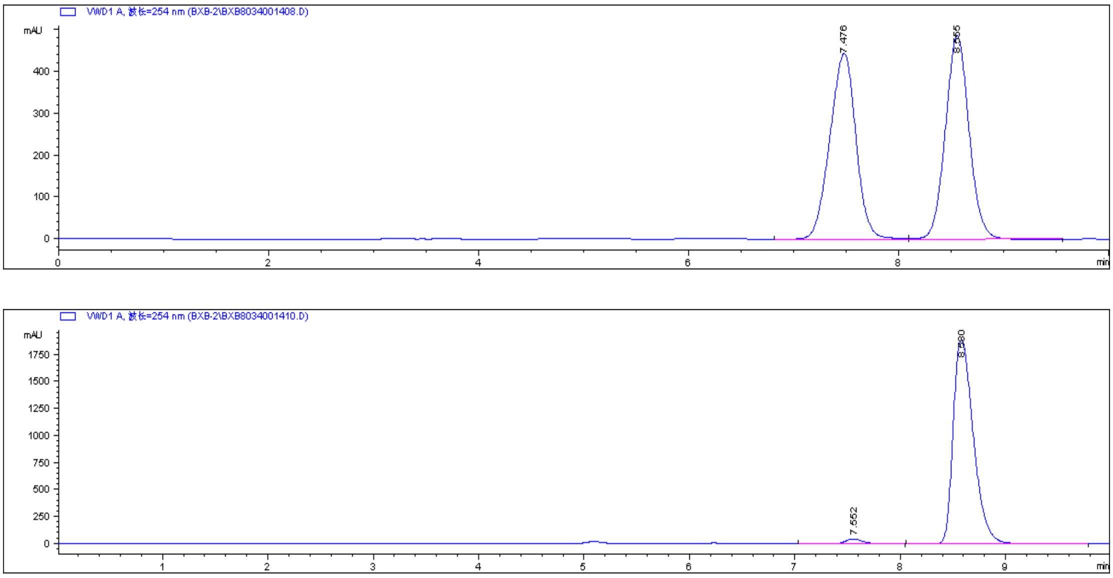

Figure S15. HPLC spectra of **5p.**

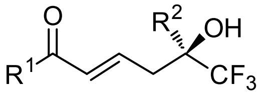

5q: R^1 = 3-ClPh
R^2 = Ph

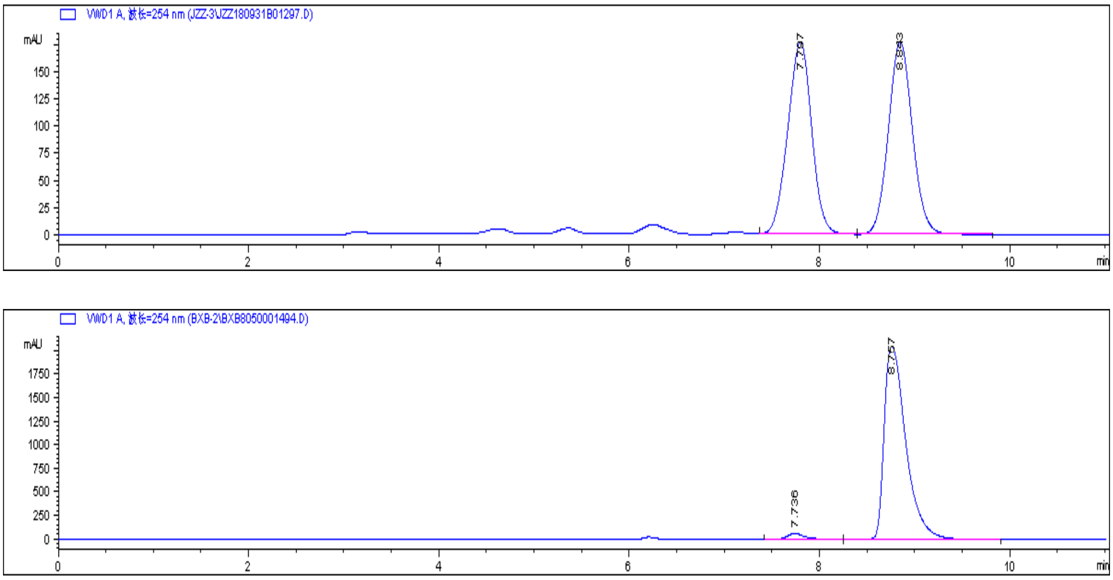

Figure S16. HPLC spectra of **5q.**

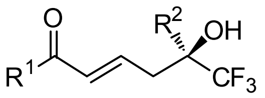

5r: R^1 = 2-FPh
R^2 = Ph

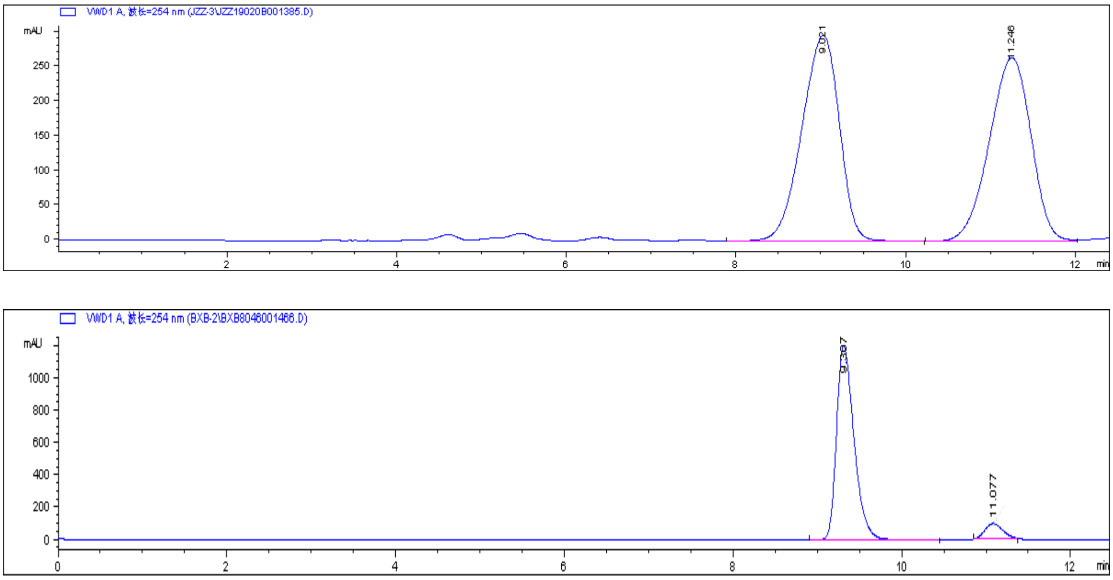

Figure S17. HPLC spectra of **5r.**

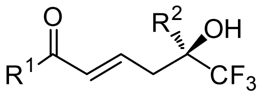

5s: R^1 = 4-MePh
R^2 = Ph

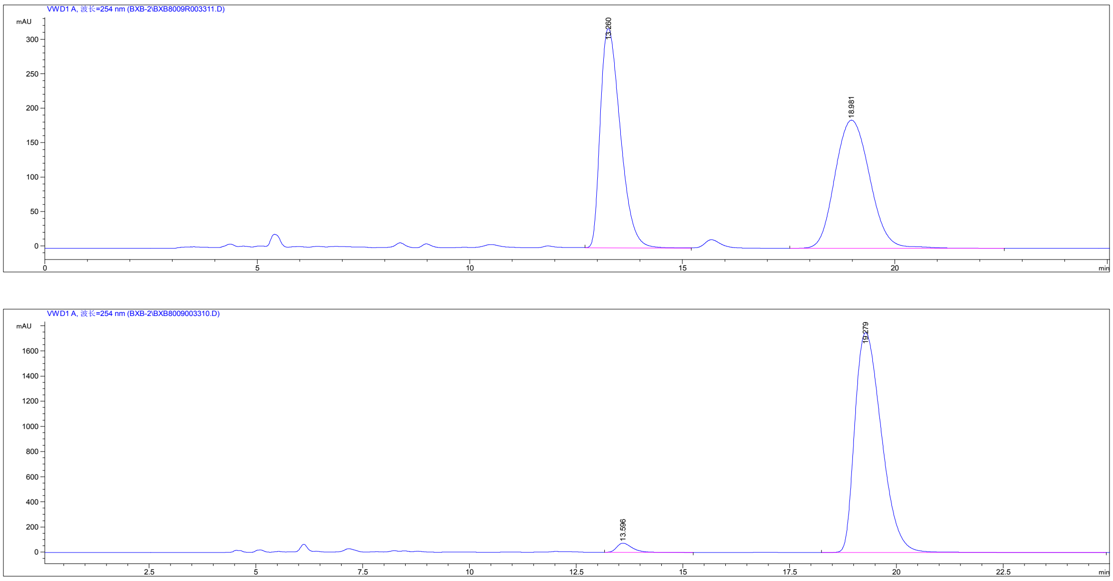

Figure S18. HPLC spectra of **5s.**

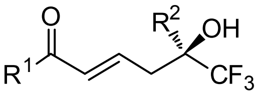

5t: R^1 = 3-MePh
R^2 = Ph

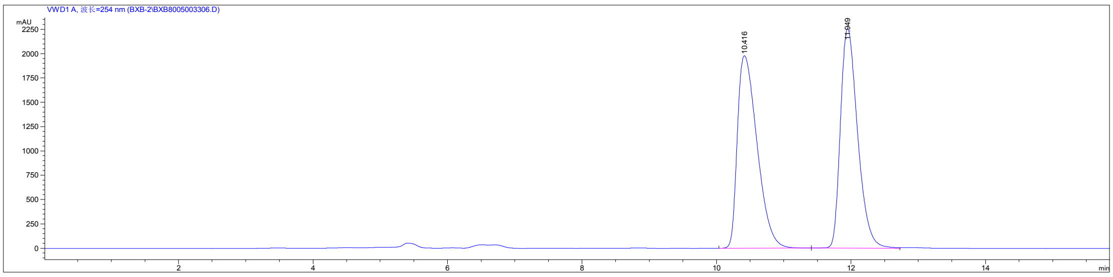

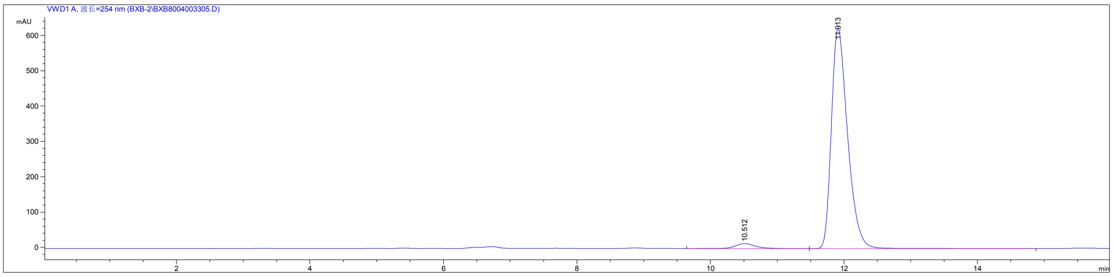

Figure S19. HPLC spectra of **5t.**

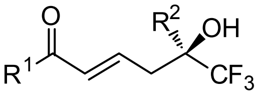

5u: R^1 = 4-MeOPh
R^2 = Ph

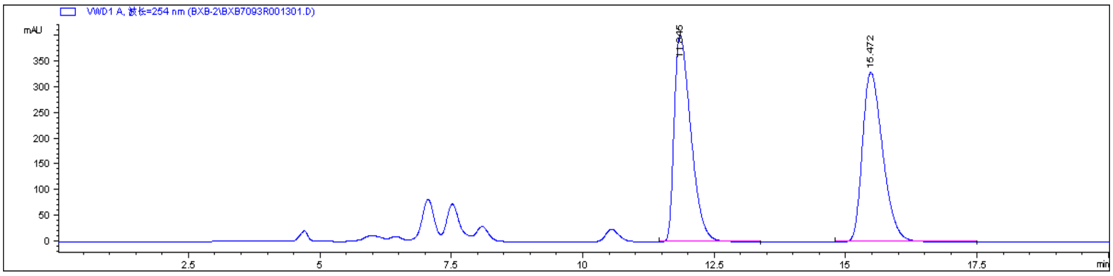

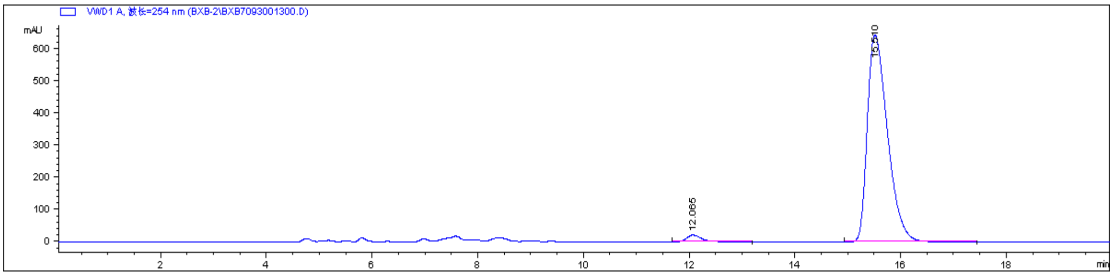

Figure S20. HPLC spectra of **5u.**

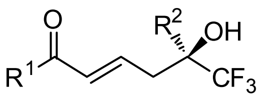

5v: R^1 = 3-MeOPh
R^2 = Ph

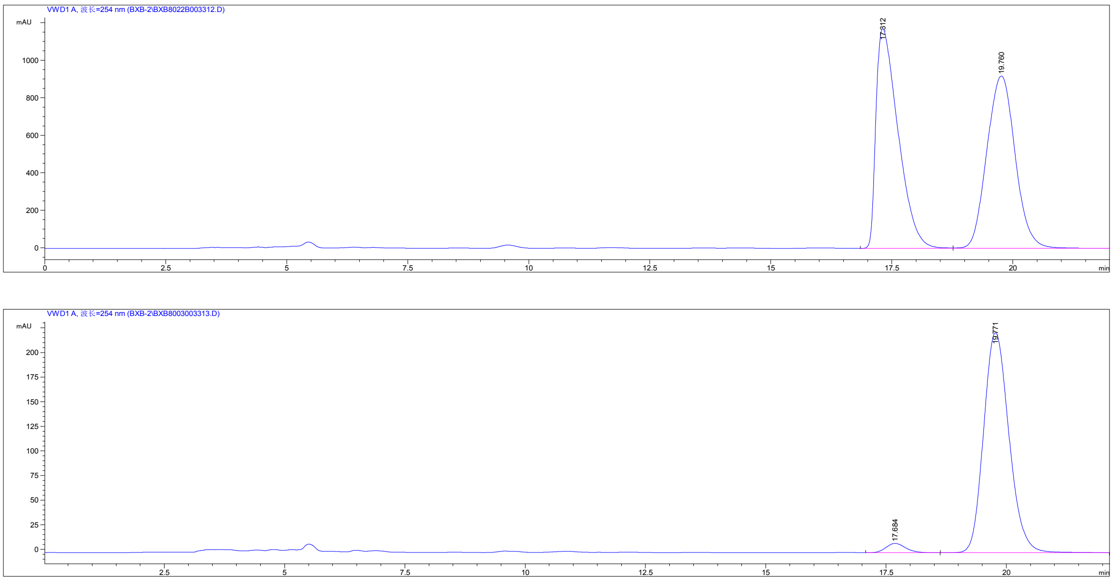

Figure S21. HPLC spectra of **5v.**

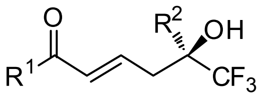

5w: R^1 = 2-MeOPh
R^2 = Ph

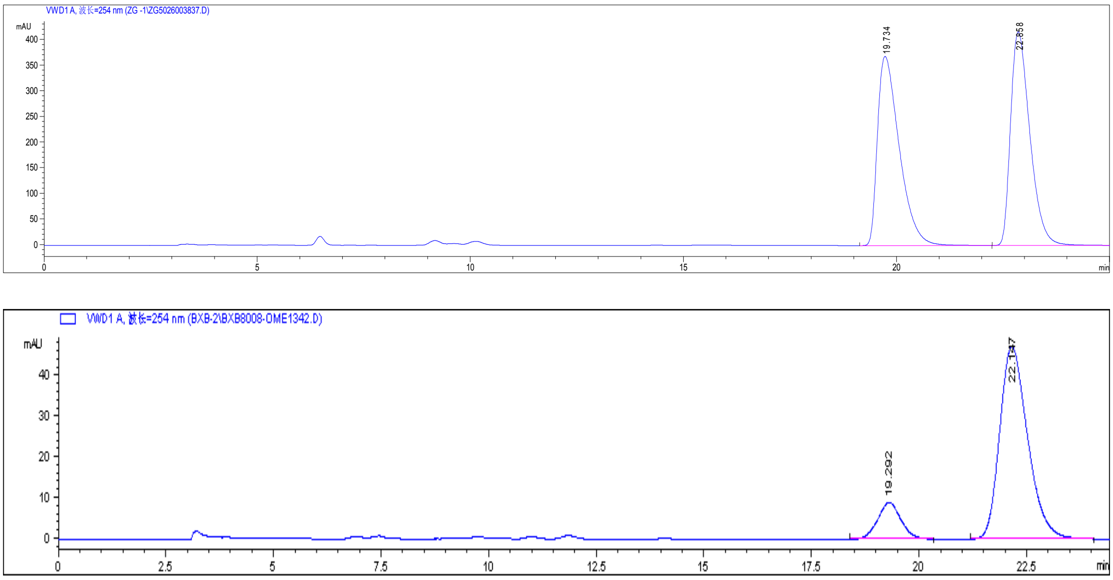

Figure S22. HPLC spectra of **5w.**

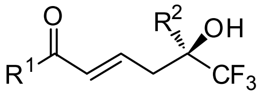

5x: R^1 = 2-naphtyl
R^2 = Ph

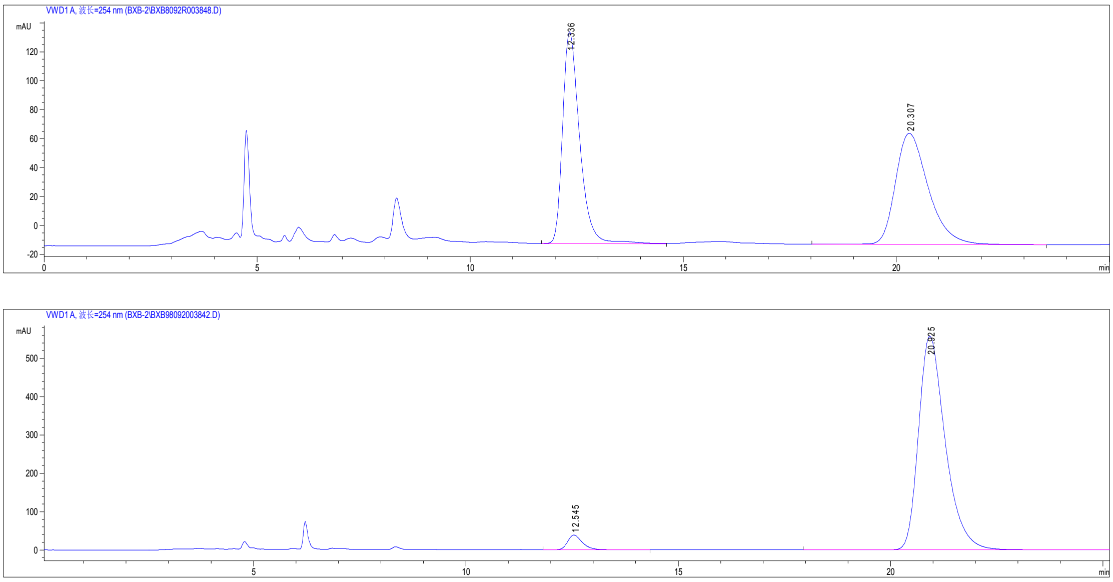

Figure S23. HPLC spectra of **5x.**

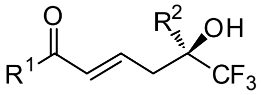

5y: R^1 = 2-thienyl
R^2 = Ph

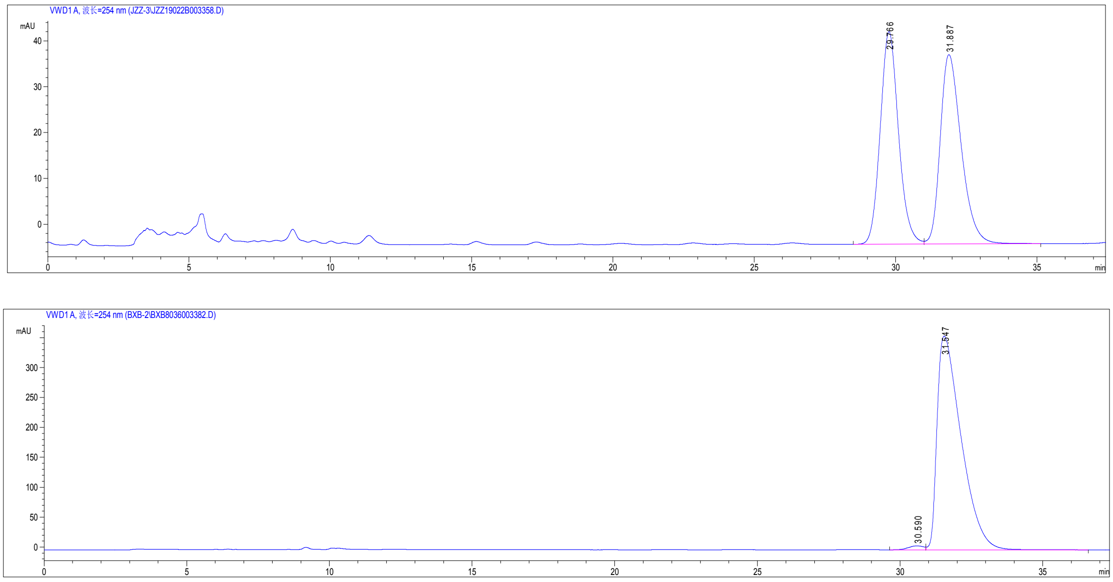

Figure S24. HPLC spectra of **5y.**

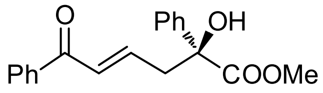

7a

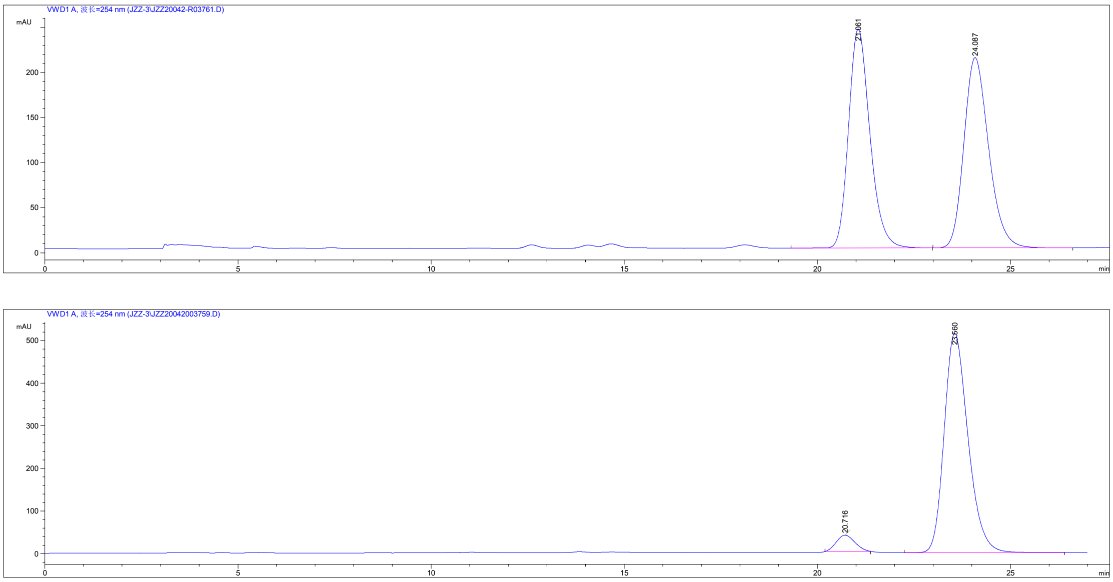

Figure S25. HPLC spectra of **7a.**

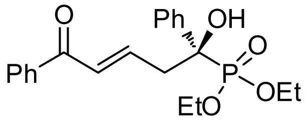

9a

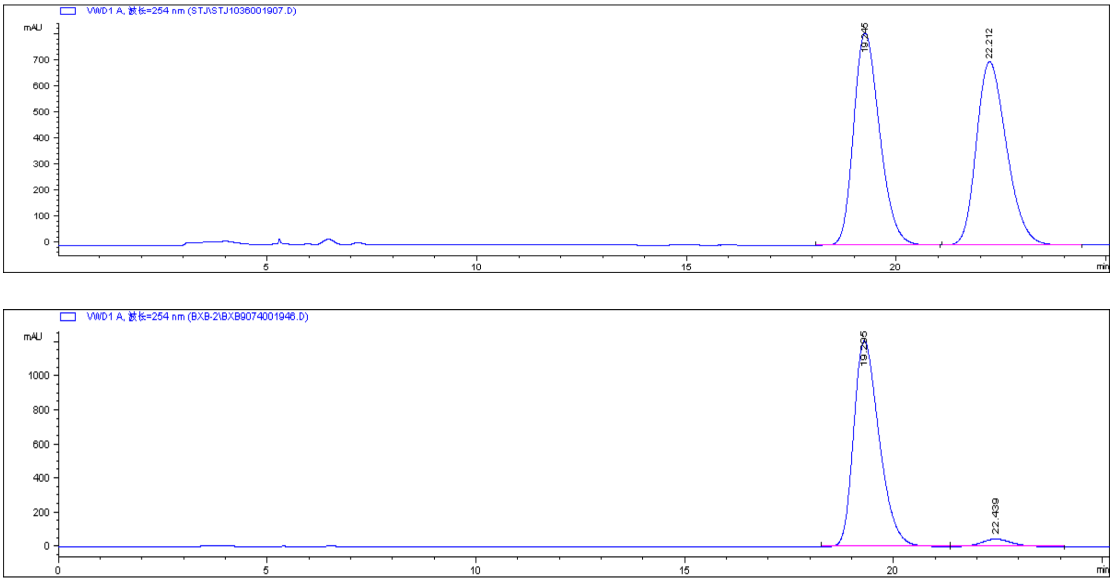

Figure S26. HPLC spectra of **9a.**

3. Proposed Mechanism

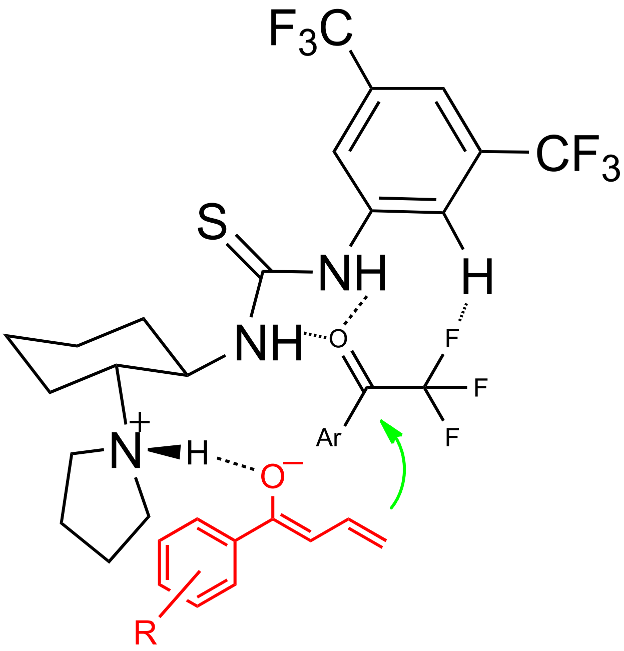

Figure S27. The proposed mechanism.

After abstraction of α-proton of allyl aryl ketones, the generated dienolates of allyl aryl ketones should bind to the R_4N^+ arm of the catalyst via electrostatic interaction. The thiourea group of the catalyst could simultaneously interact with trifluoromethyl ketones via two hydrogen bonds. A non-classic hydrogen bond between C–F bond of trifluoromethyl ketones and C–H bond of aryl substituent of thiourea leads to the most stable interaction. After nucleophilic addition, the vinylogous aldol adducts were obtained with the observed enantioselective results. The ortho-substituents on phenyl rings of allyl aryl ketones should increase the steric effect with pyrrole ring of the catalyst, leading to poor enantioselective results.

4. Copies of NMR Spectra

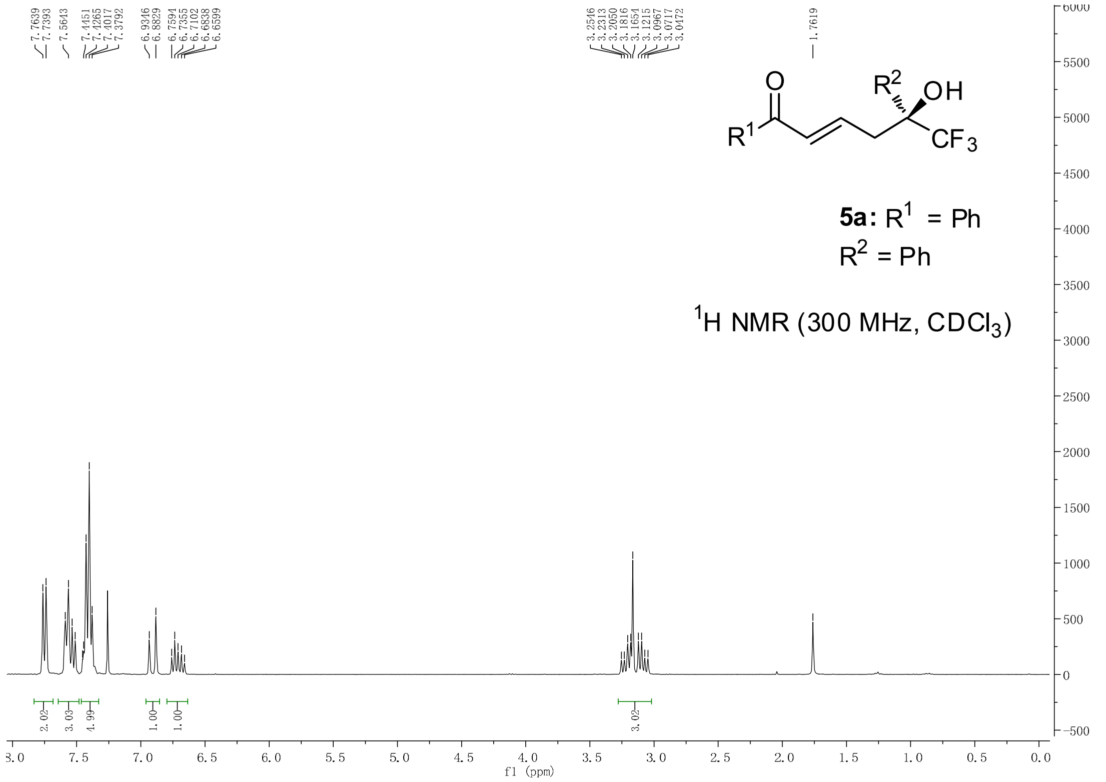

Figure S28 ^{1}H NMR spectrum of **5a.**

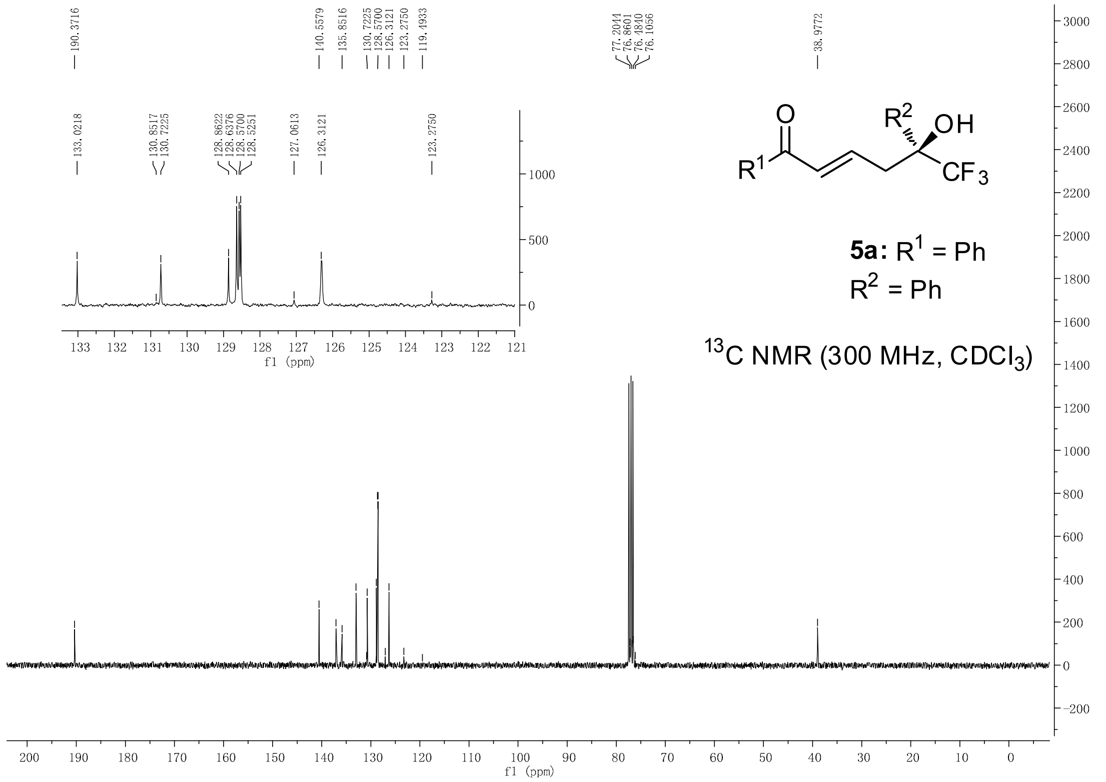

Figure S29 ^{13}C NMR spectrum of **5a.**

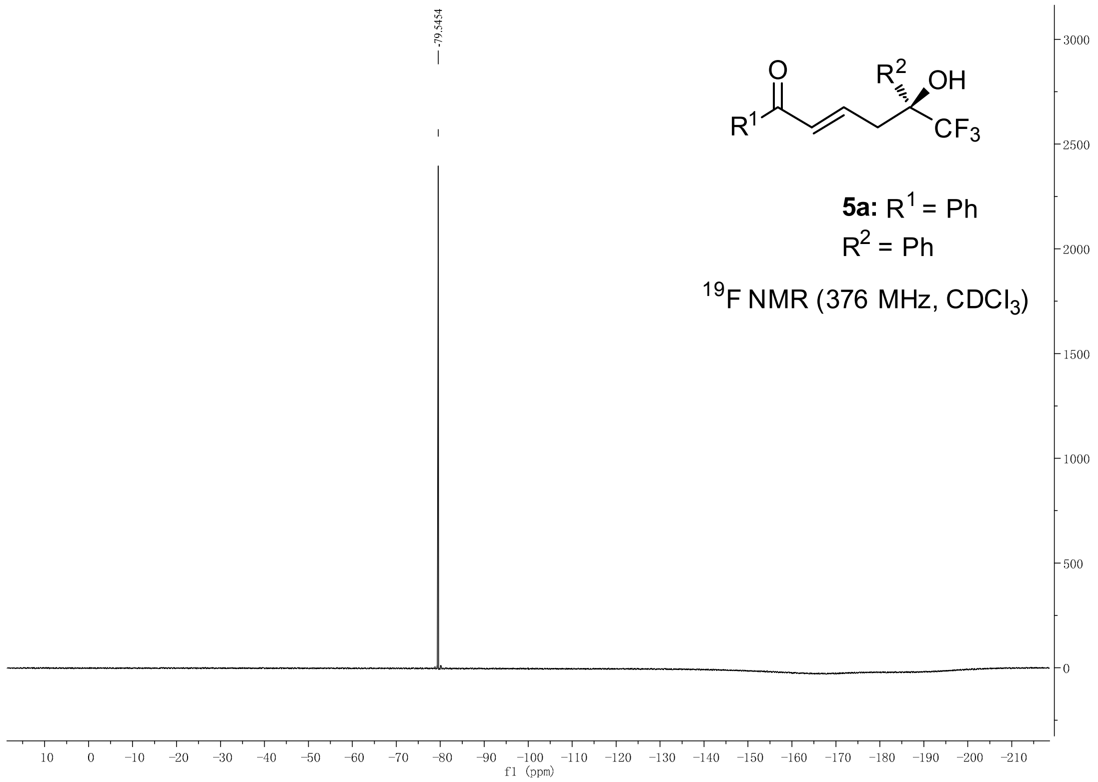

Figure S30 ^{19}F NMR spectrum of **5a.**

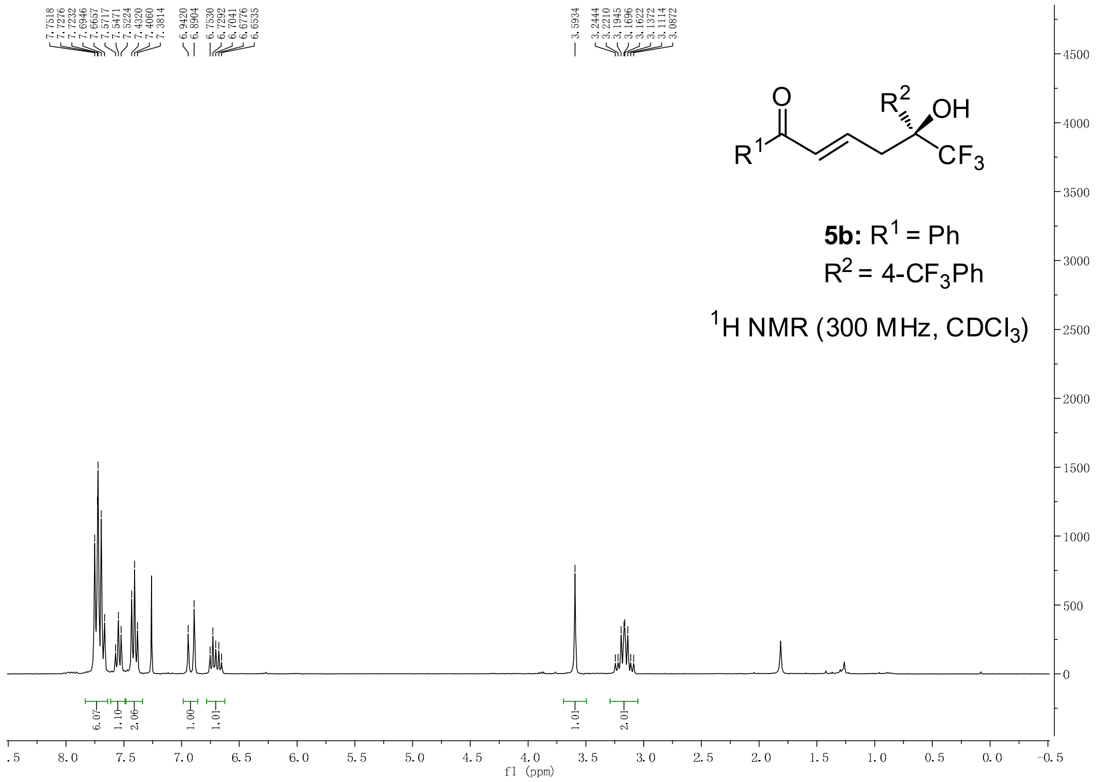

Figure S31 ^{1}H NMR spectrum of **5b.**

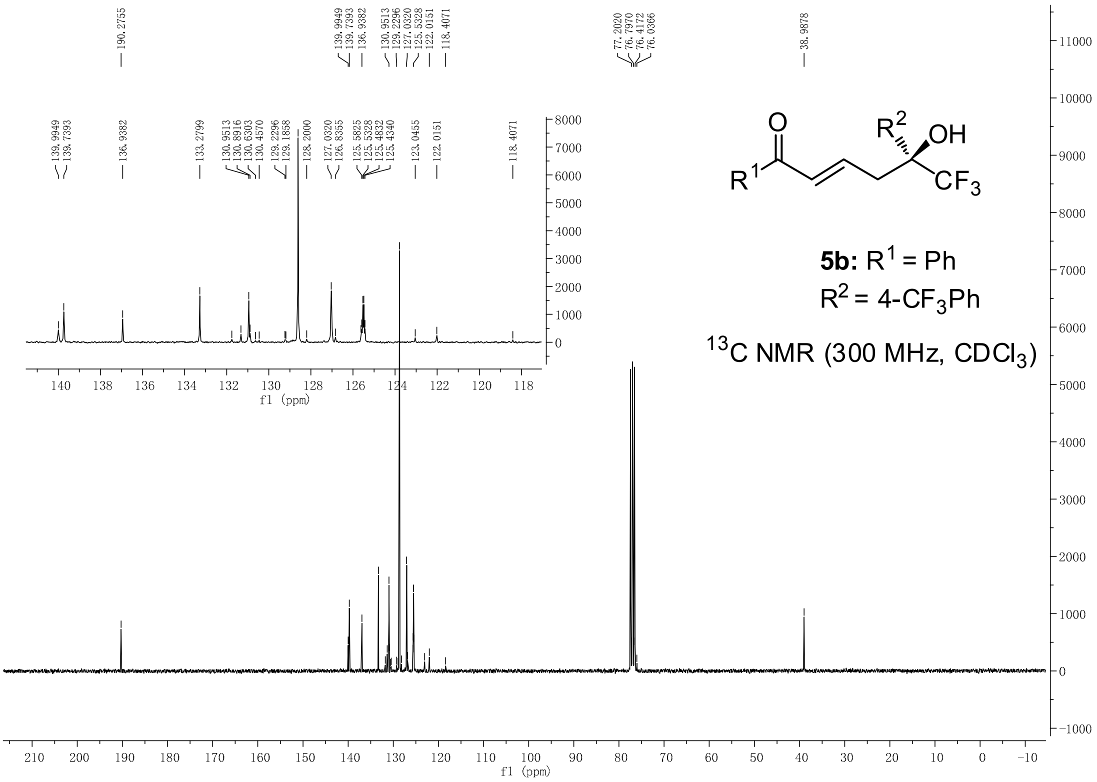

Figure S32 ^{13}C NMR spectrum of **5b.**

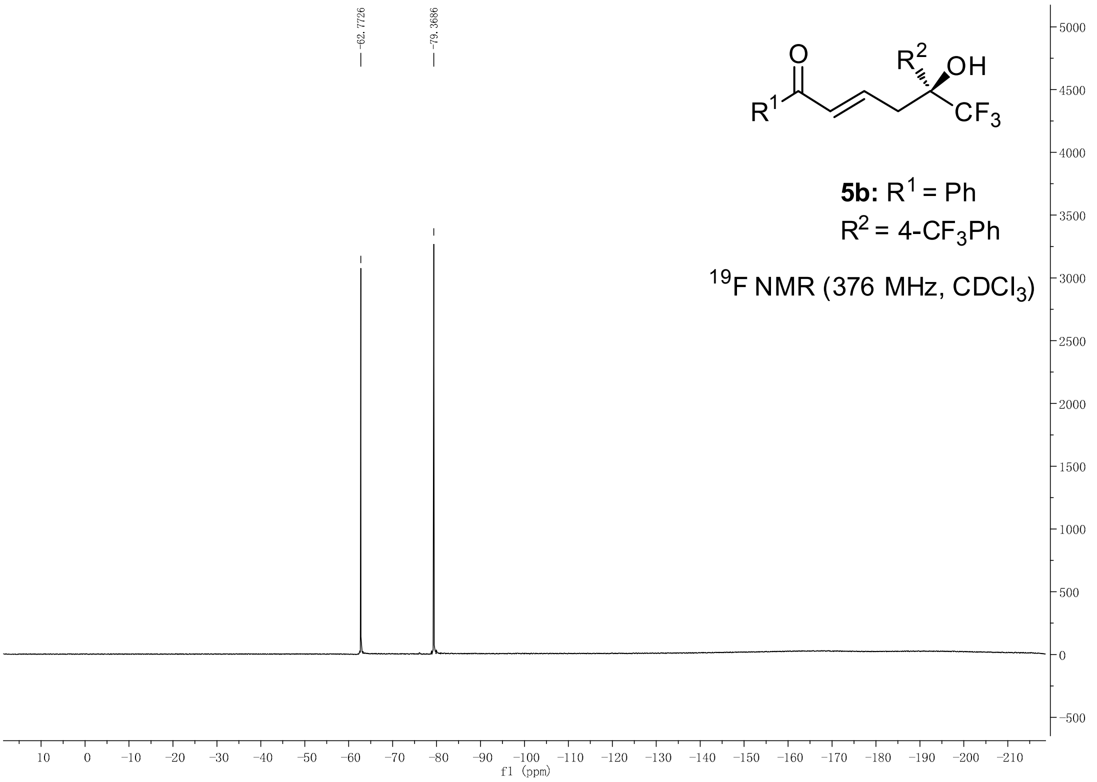

Figure S33 ^{19}F NMR spectrum of **5b.**

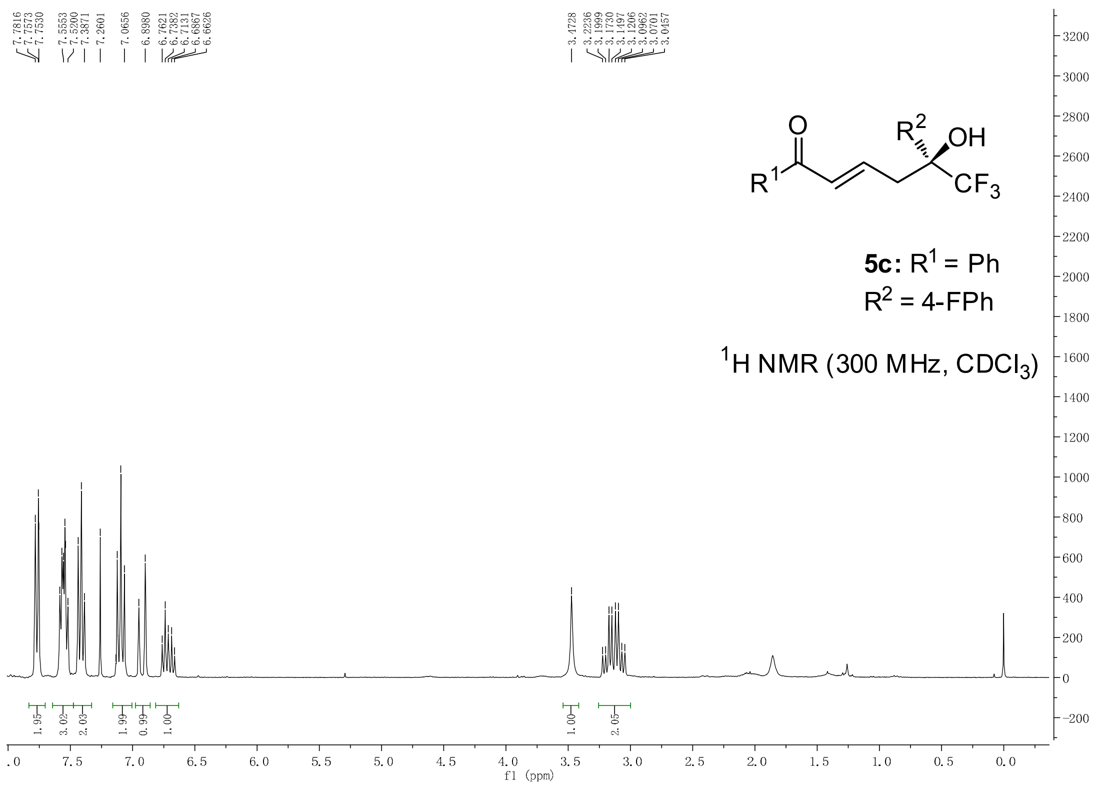

Figure S34 ^{1}H NMR spectrum of **5c.**

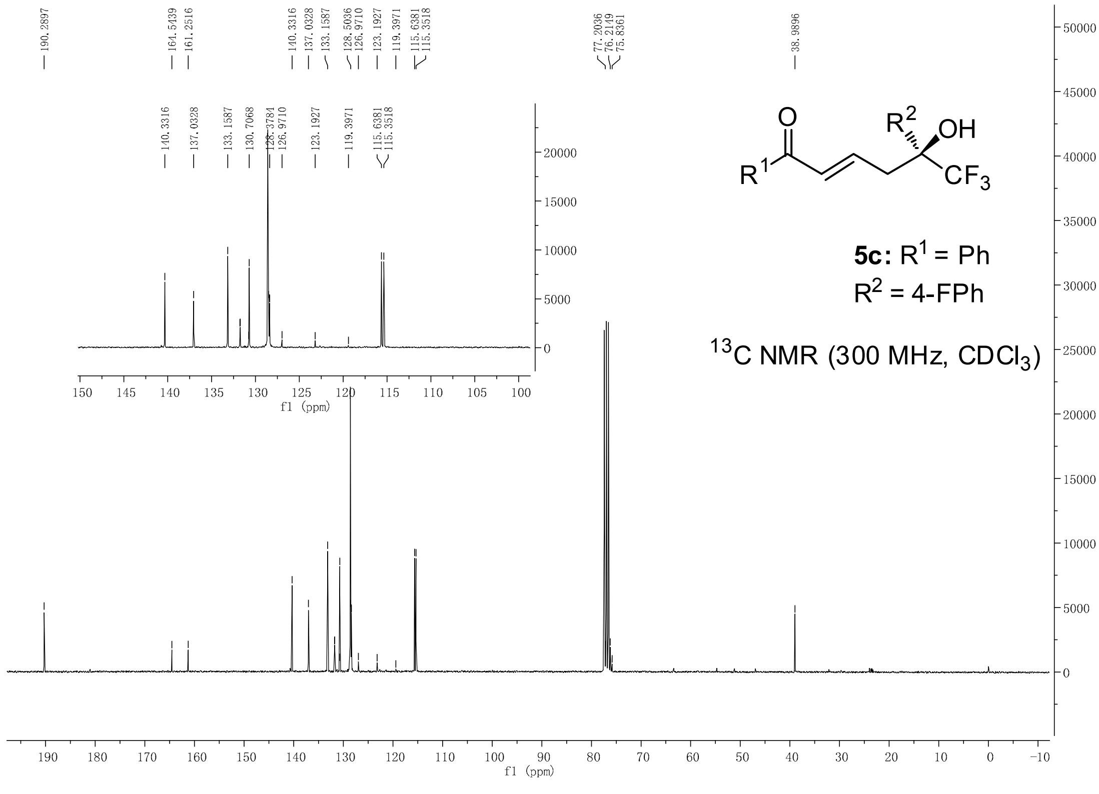

Figure S35 ^{13}C NMR spectrum of **5c.**

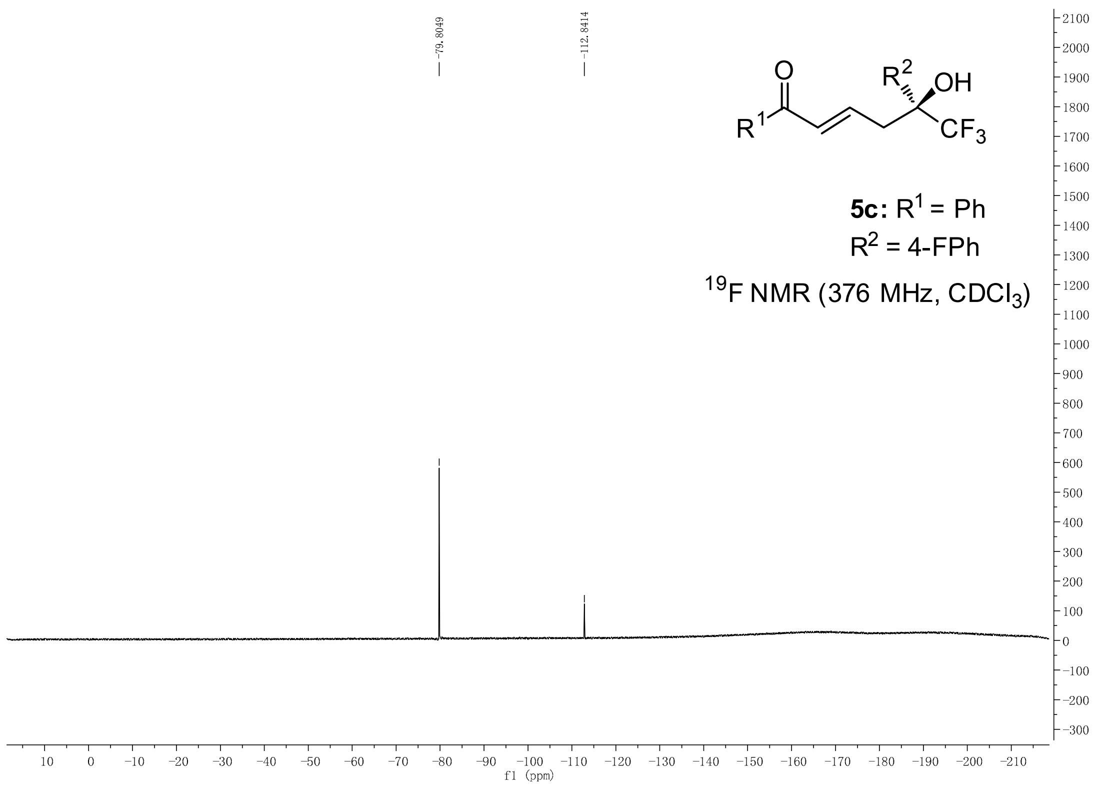

Figure S36 ^{19}F NMR spectrum of **5c.**

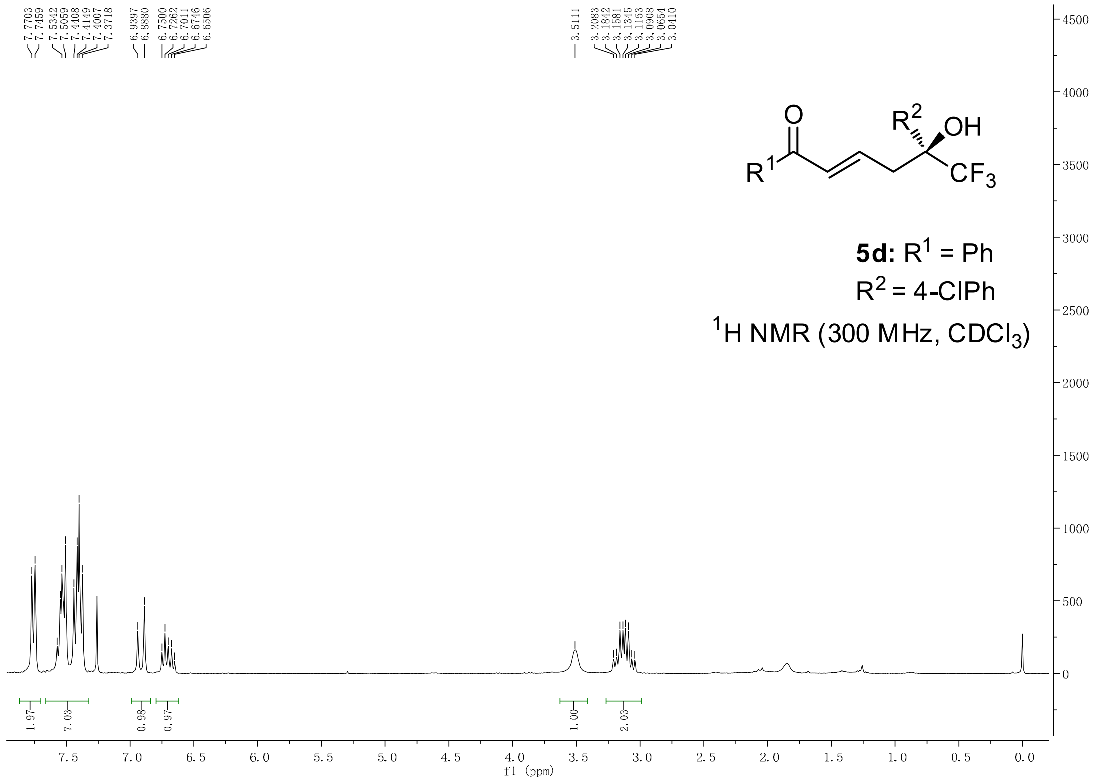

Figure S37 ^{1}H NMR spectrum of **5d.**

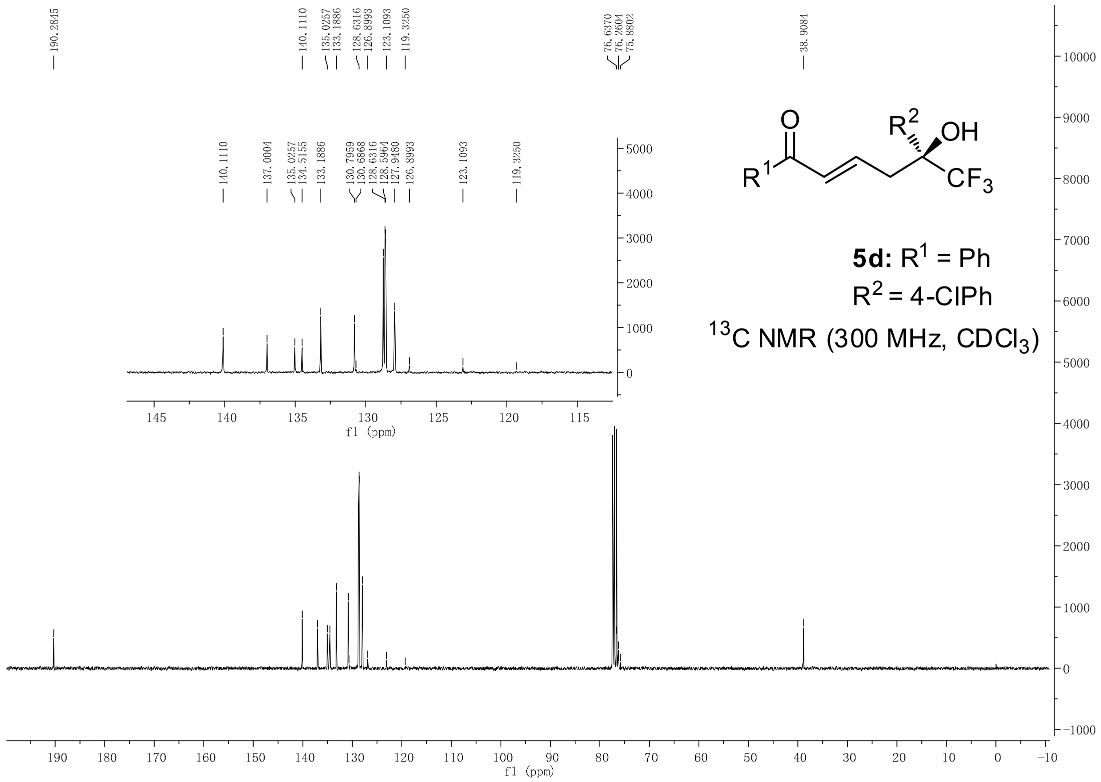

Figure S38 ^{13}C NMR spectrum of **5d.**

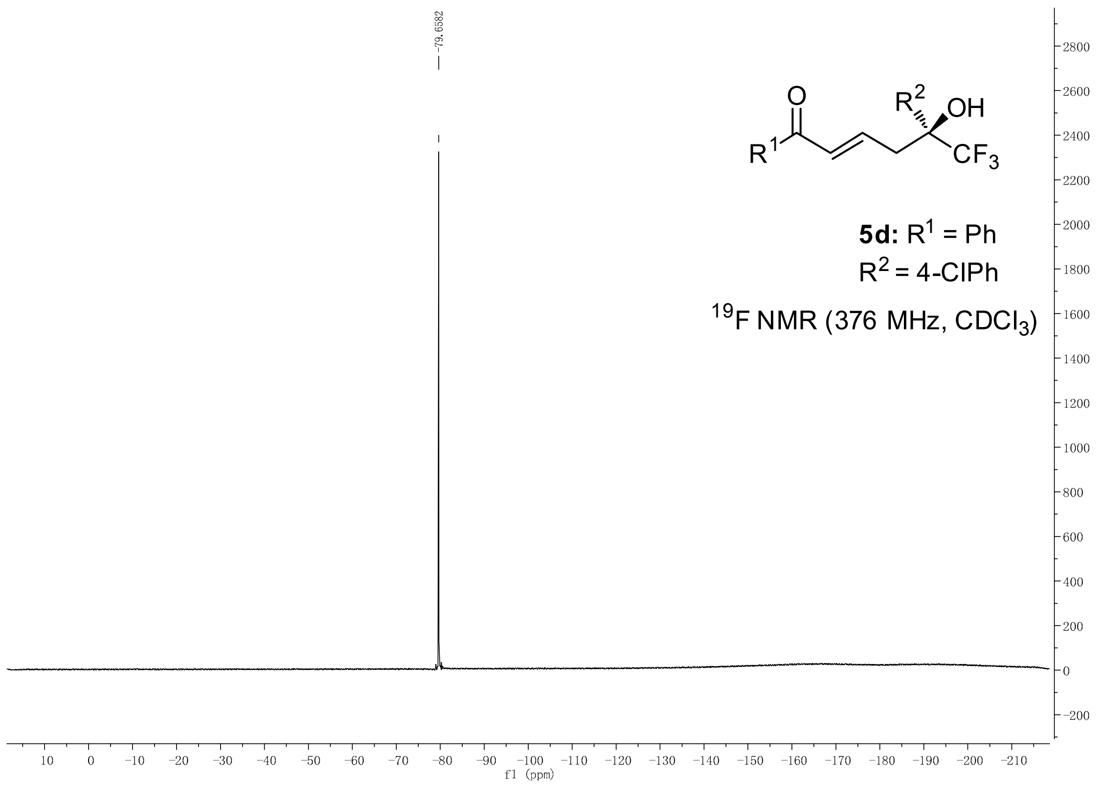

Figure S39 ^{19}F NMR spectrum of **5d.**

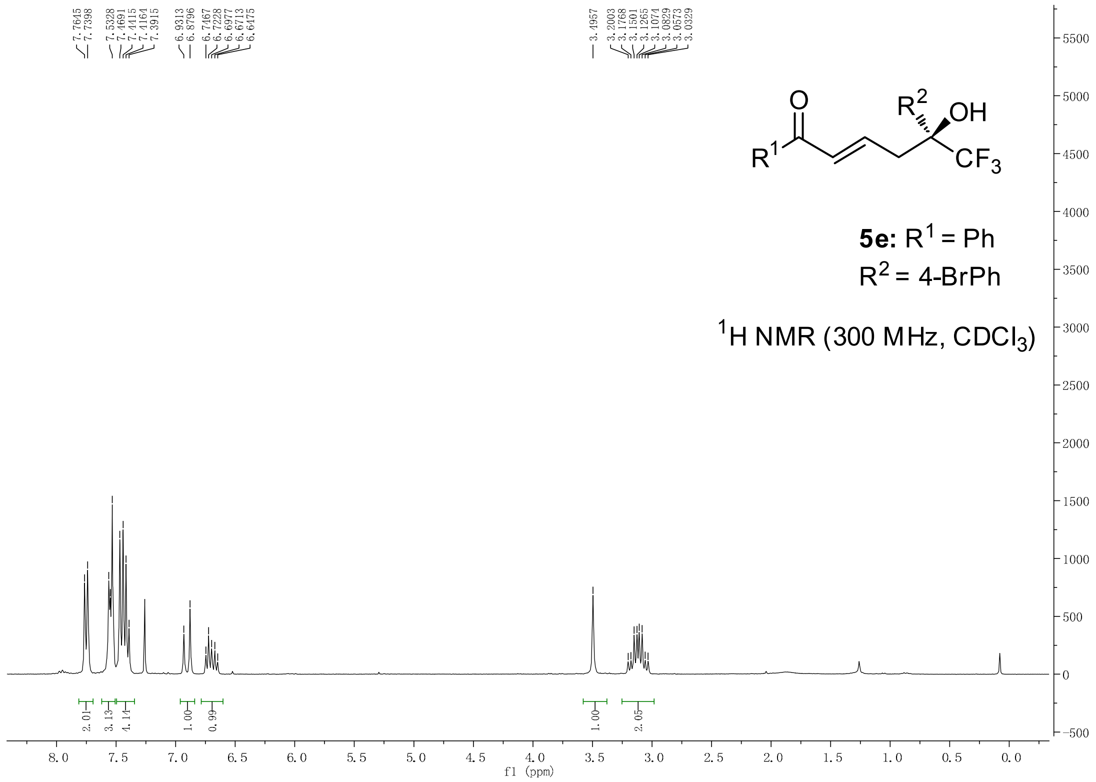

Figure S40 ^{1}H NMR spectrum of **5e.**

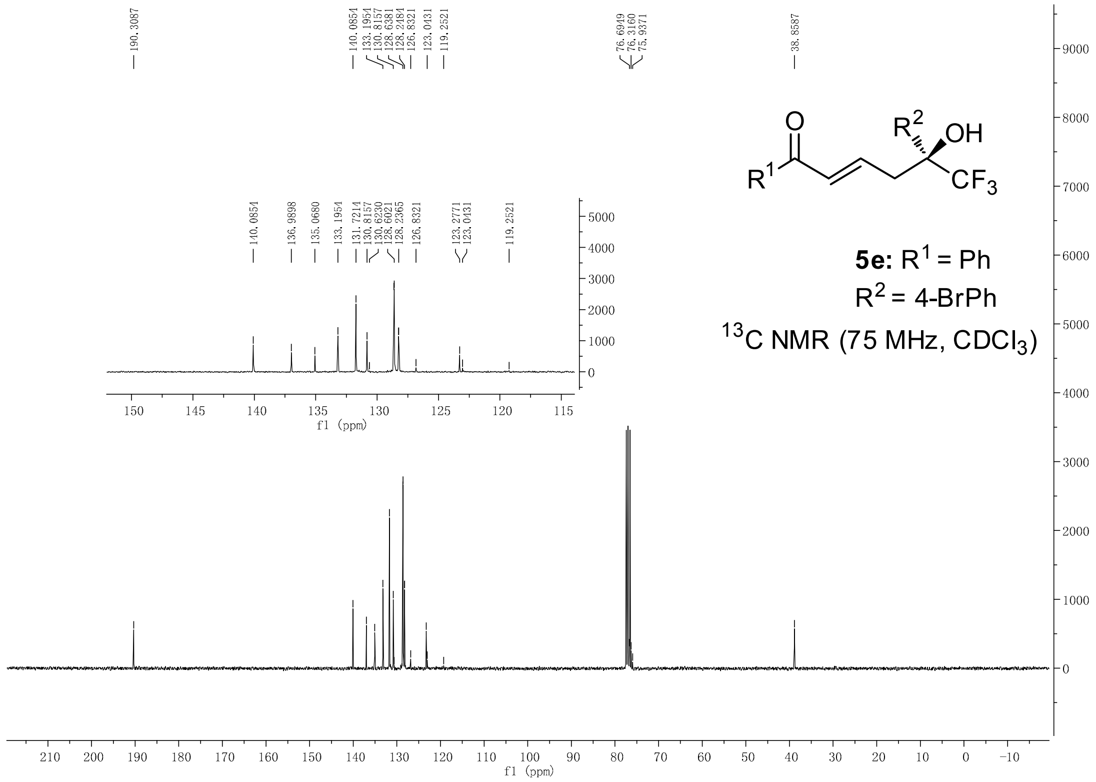

Figure S41 ^{13}C NMR spectrum of **5e.**

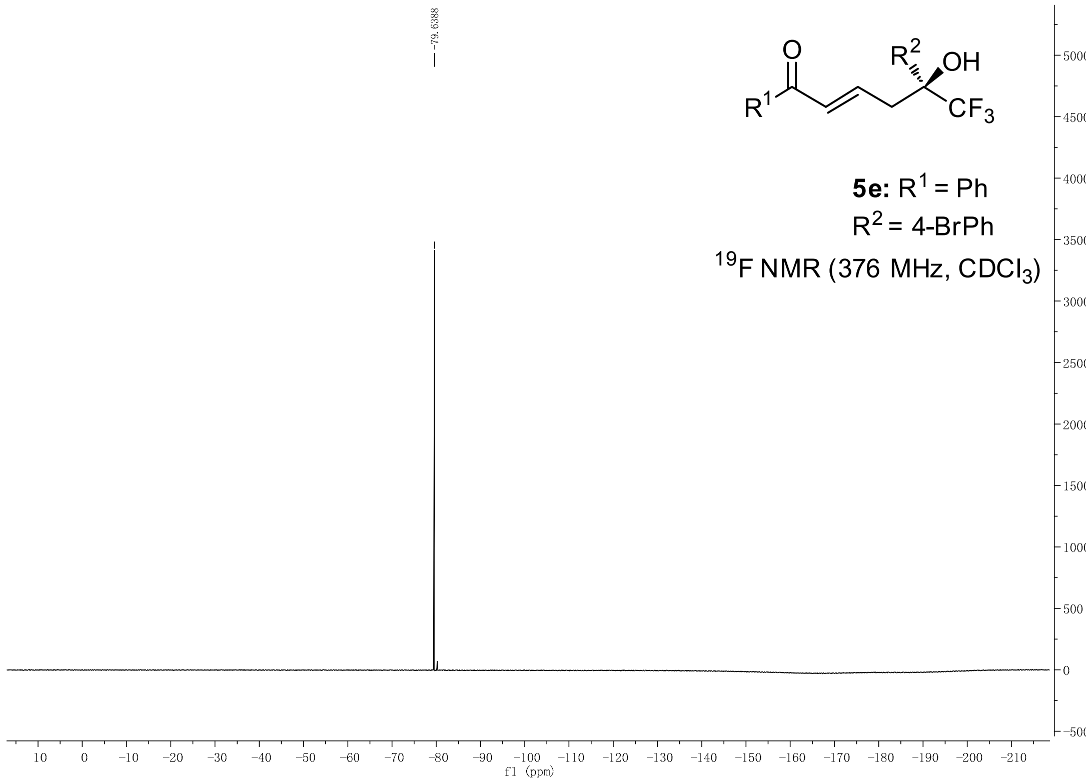

Figure S42 ^{19}F NMR spectrum of **5e.**

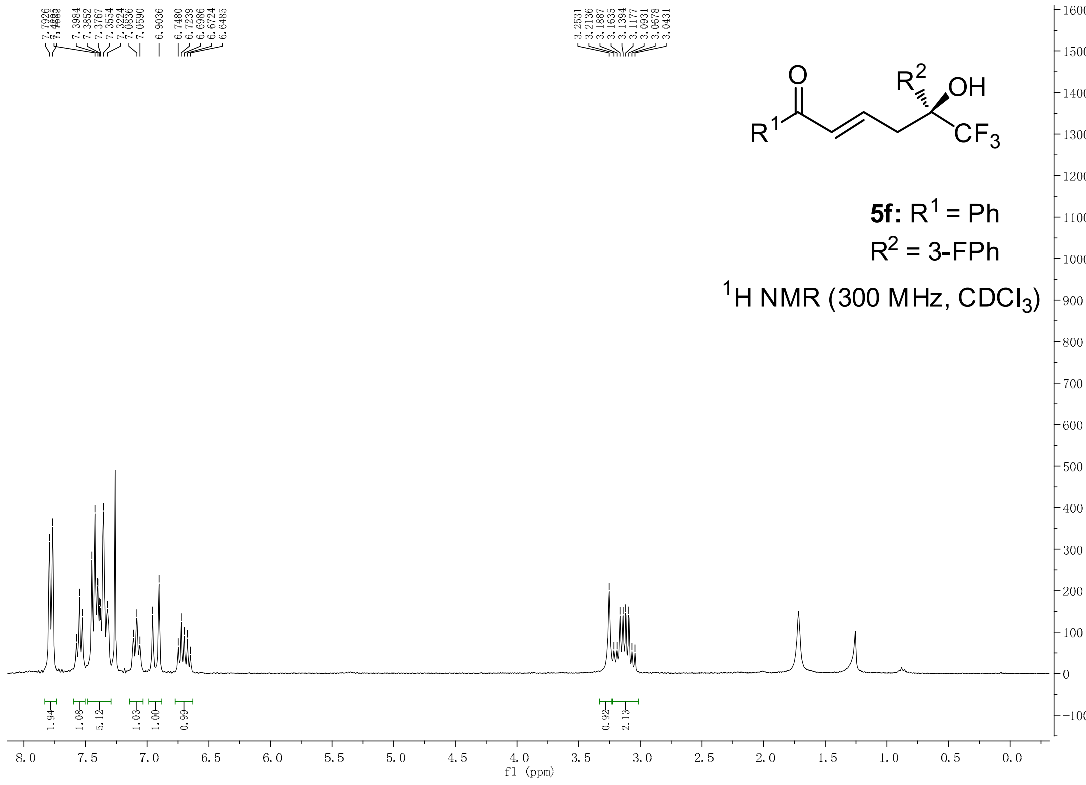

Figure S43 ^{1}H NMR spectrum of **5f.**

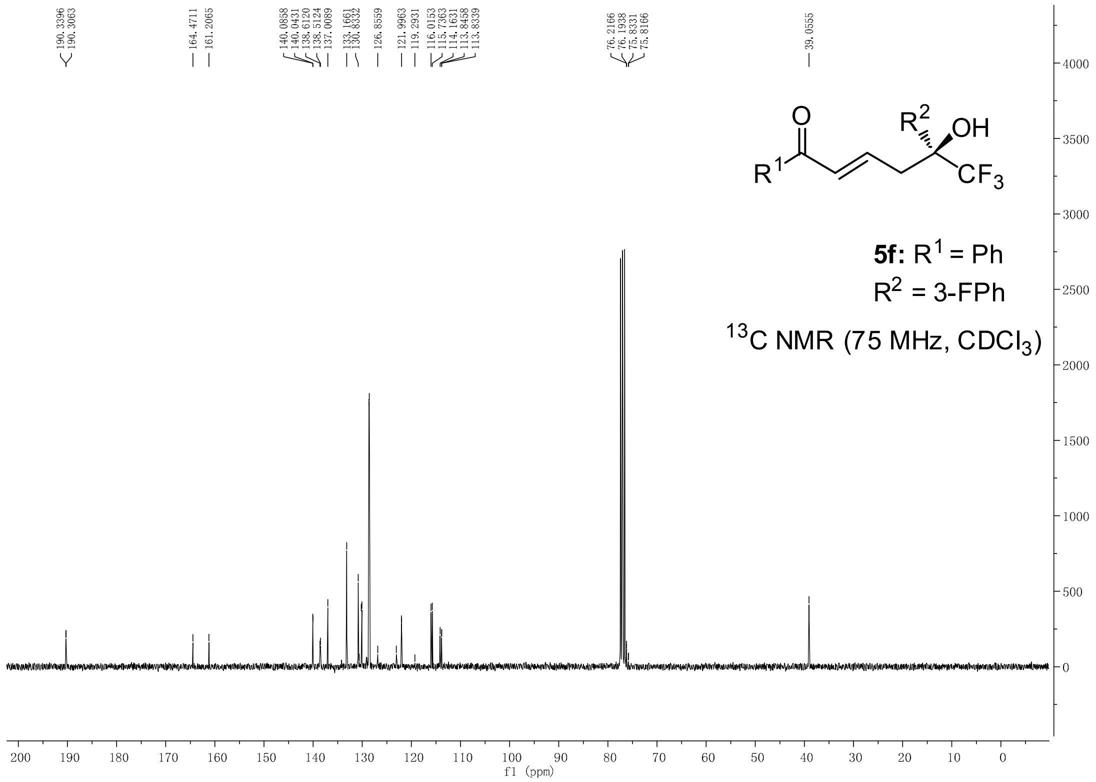

Figure S44 ^{13}C NMR spectrum of **5f.**

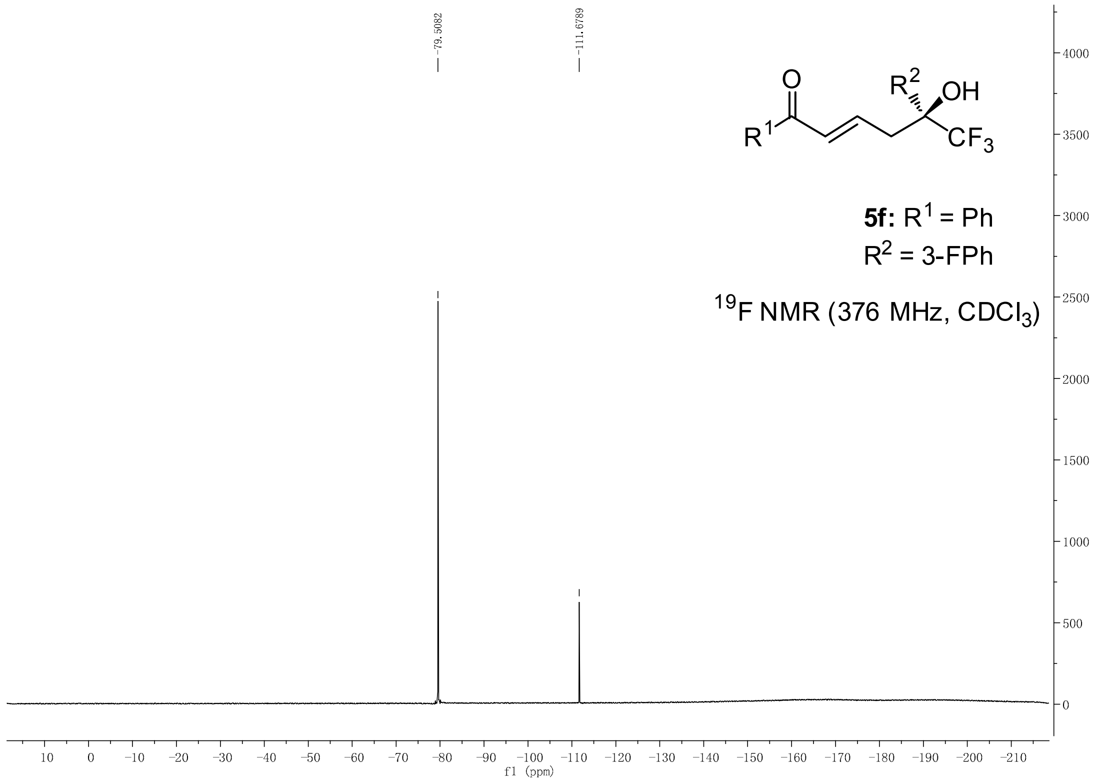

Figure S45 ^{19}F NMR spectrum of **5f.**

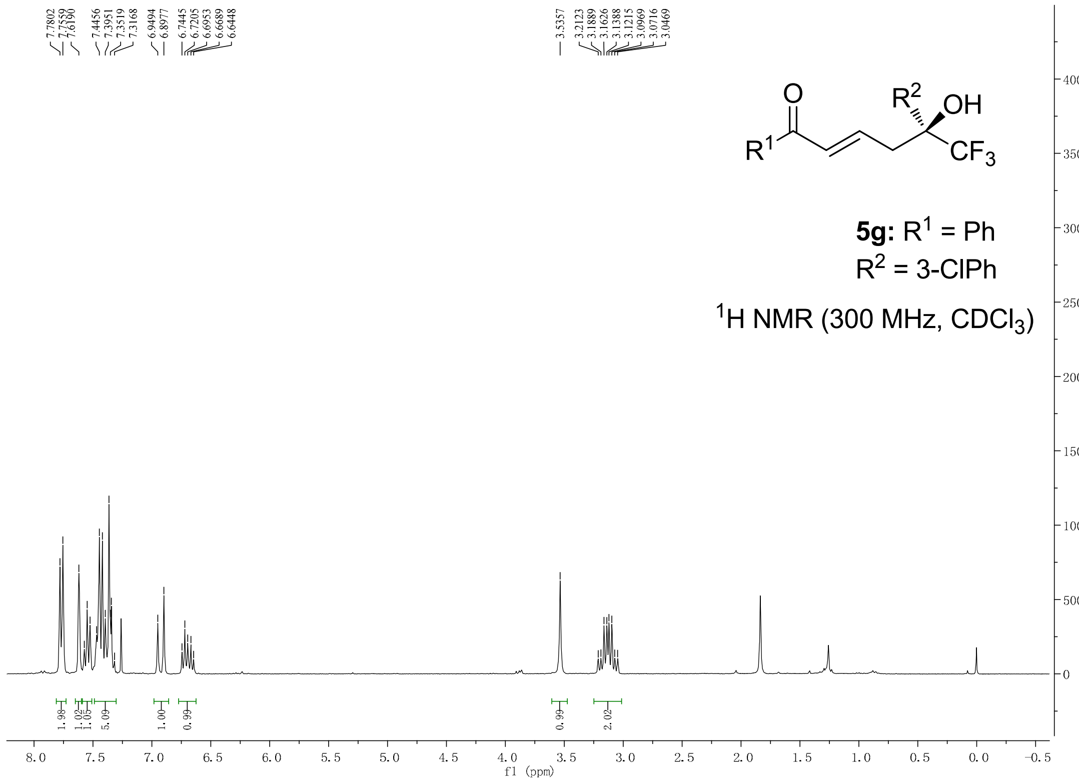

Figure S46 ^{1}H NMR spectrum of **5g.**

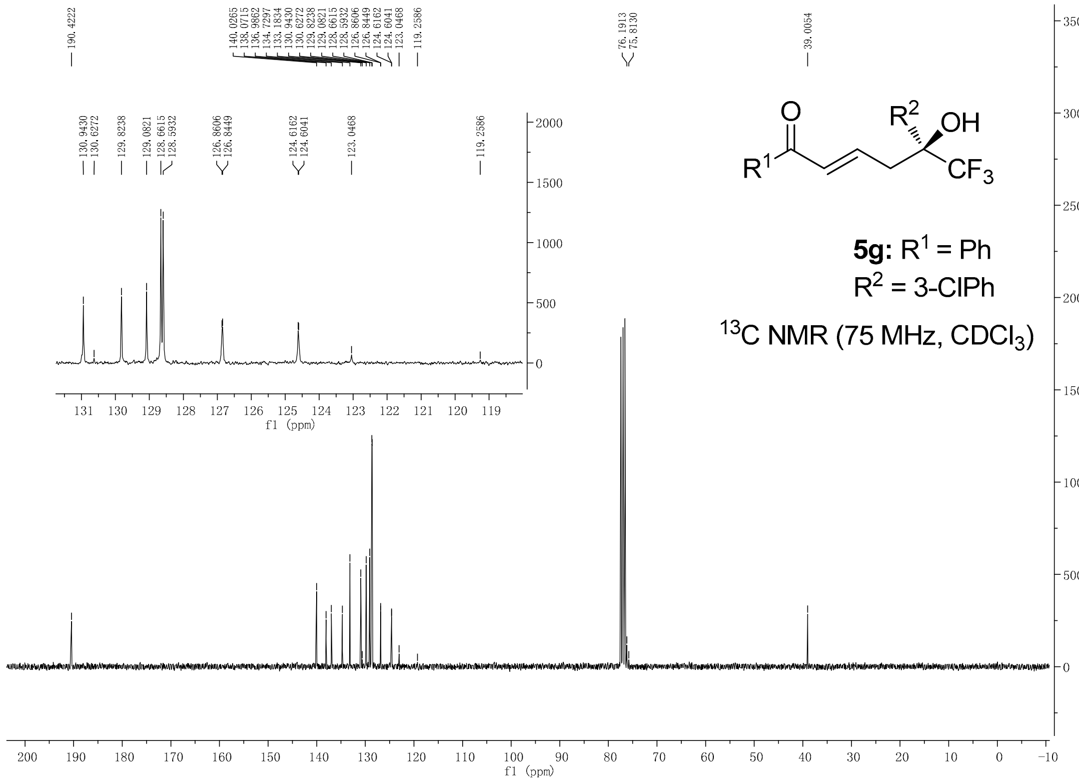

Figure S47 ^{13}C NMR spectrum of **5g.**

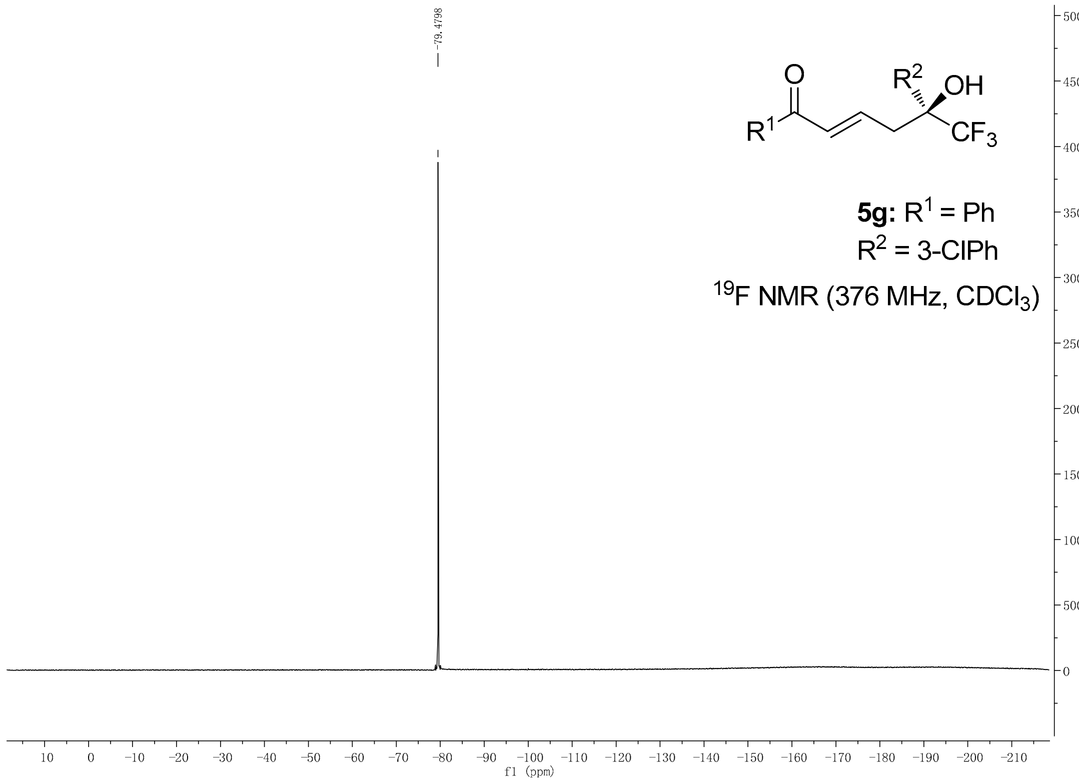

Figure S48 ^{19}F NMR spectrum of **5g.**

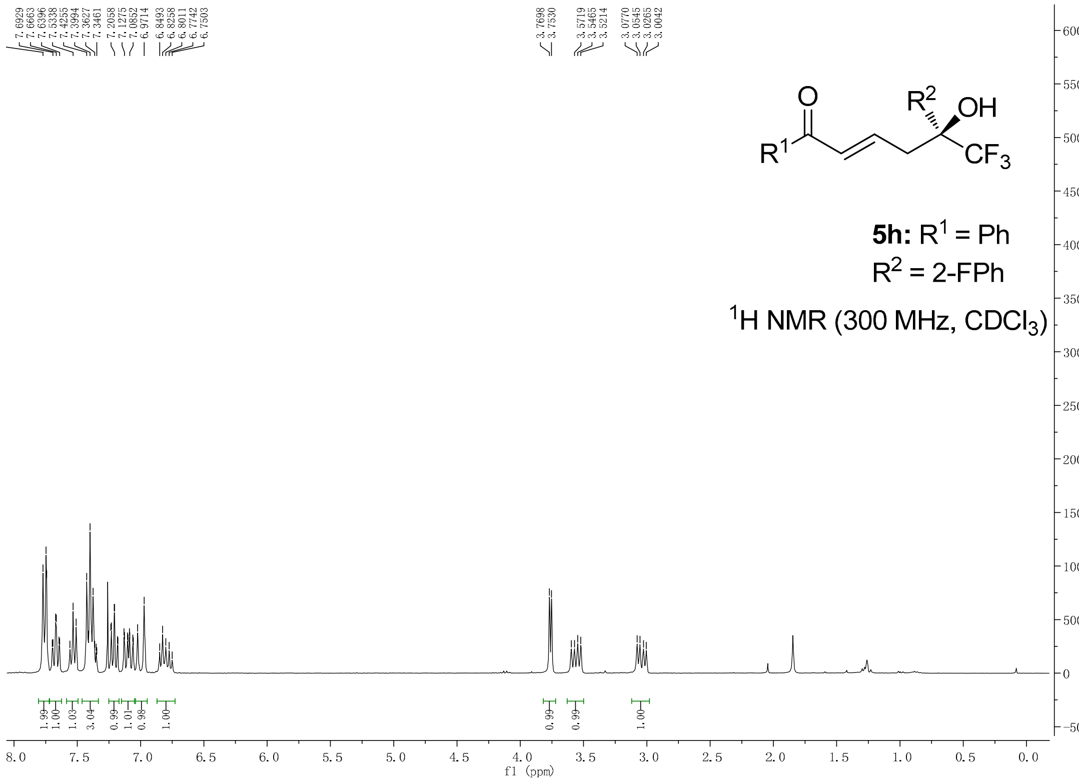

Figure S49 ^{1}H NMR spectrum of **5h.**

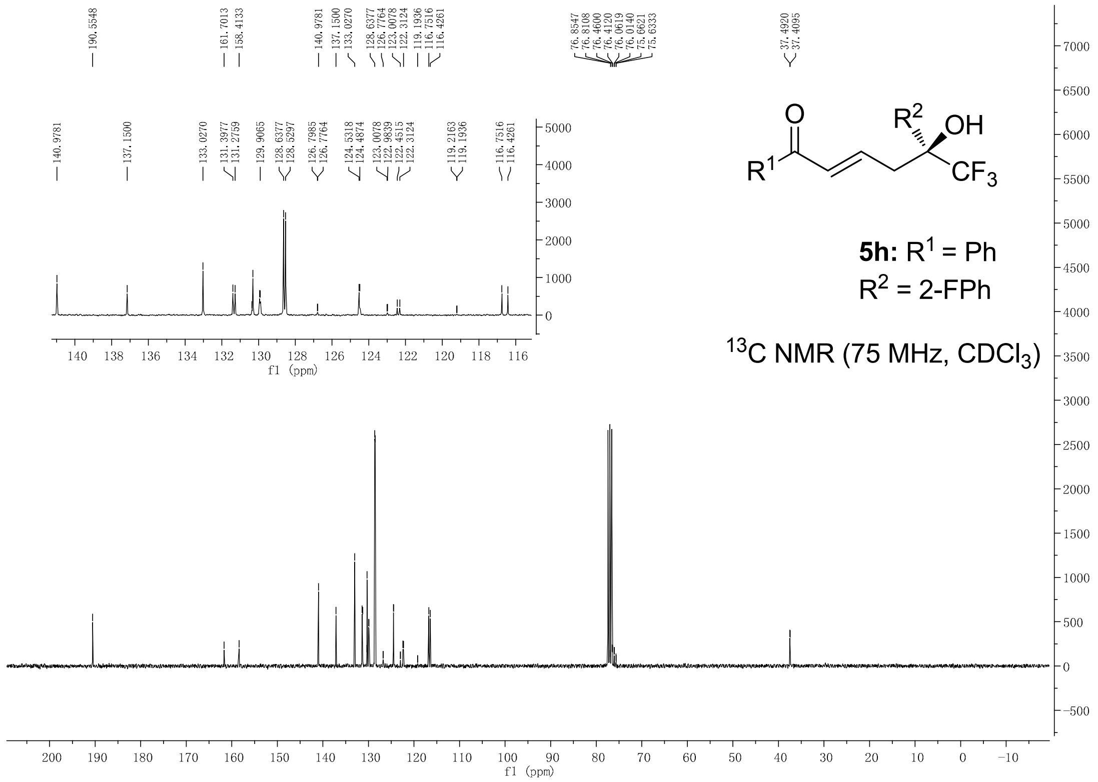

Figure S50 ^{13}C NMR spectrum of **5h.**

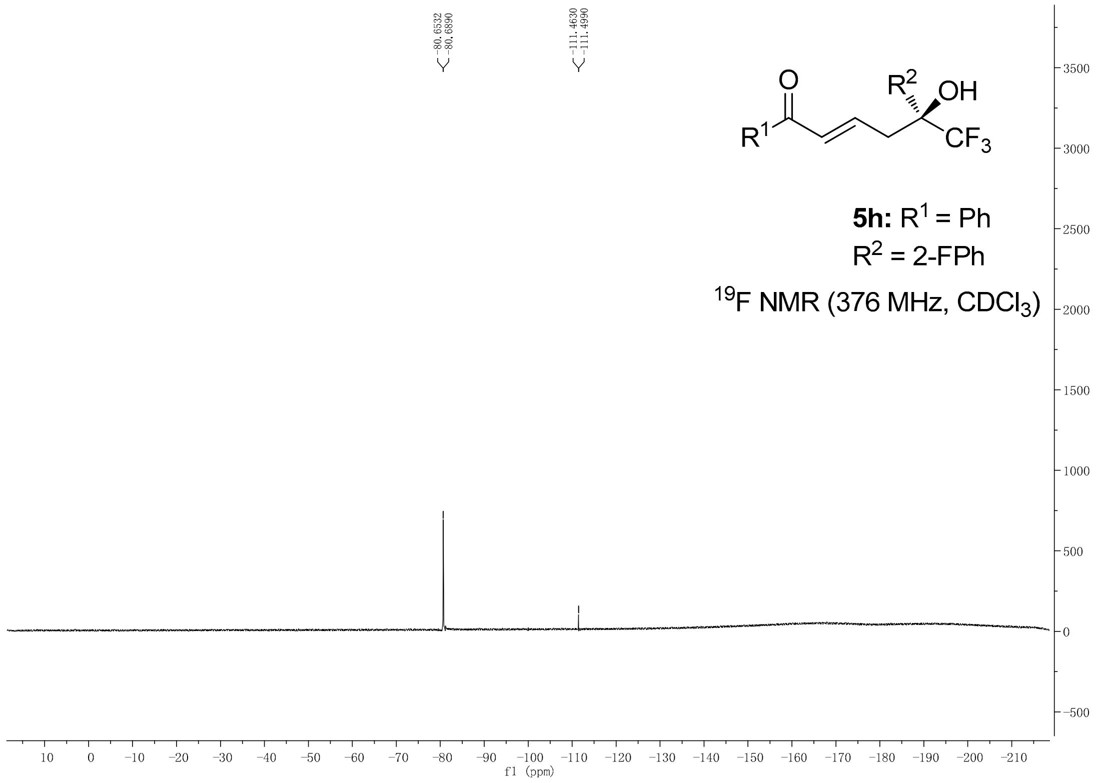

Figure S51 ^{19}F NMR spectrum of **5h.**

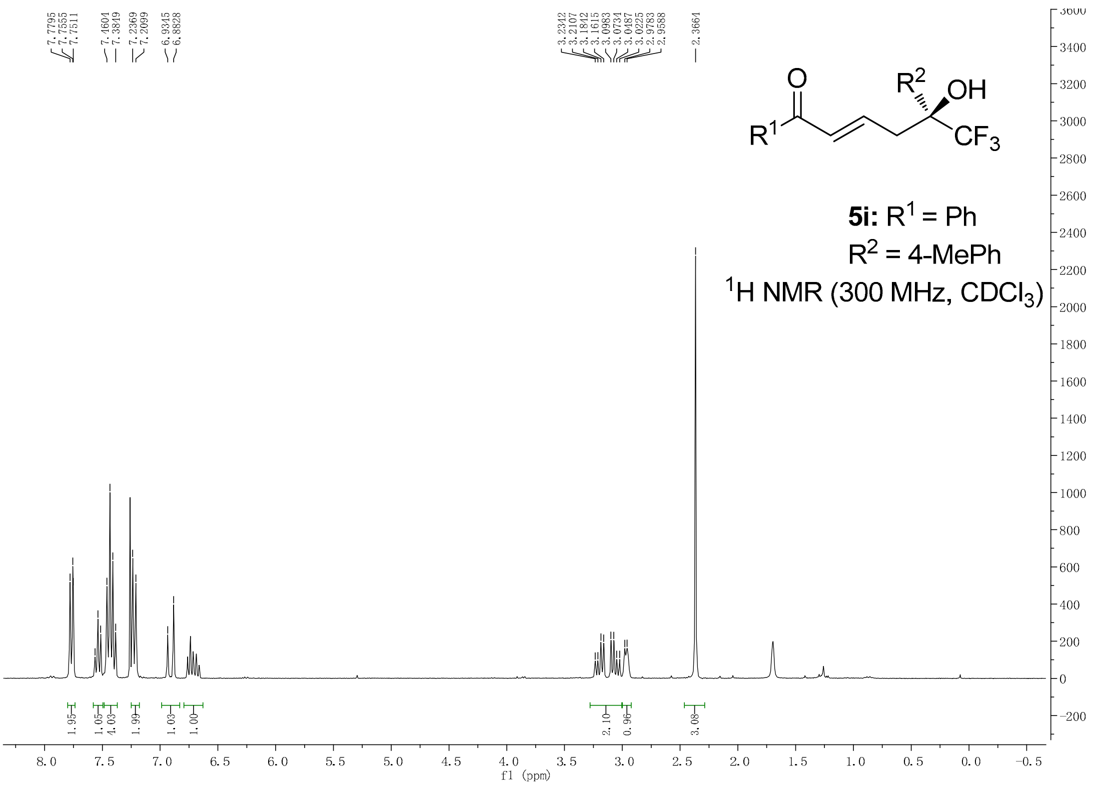

Figure S52 ¹H NMR spectrum of **5i.**

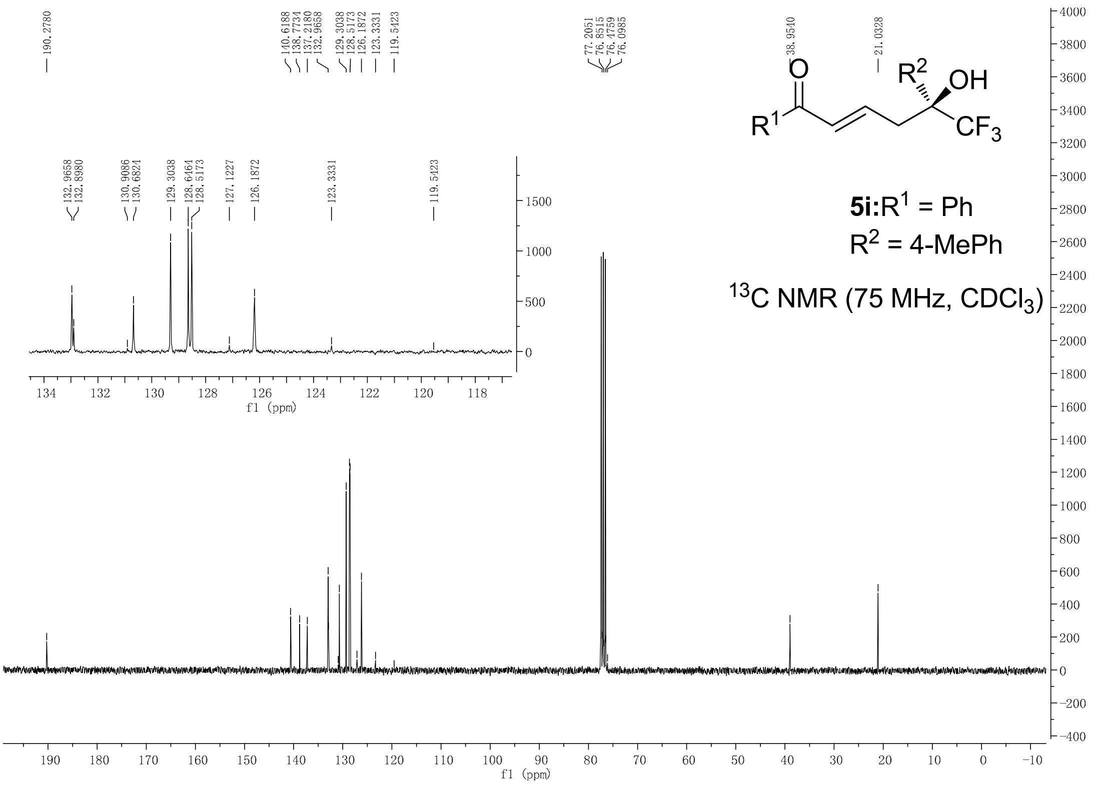

Figure S53 ¹³C NMR spectrum of **5i.**

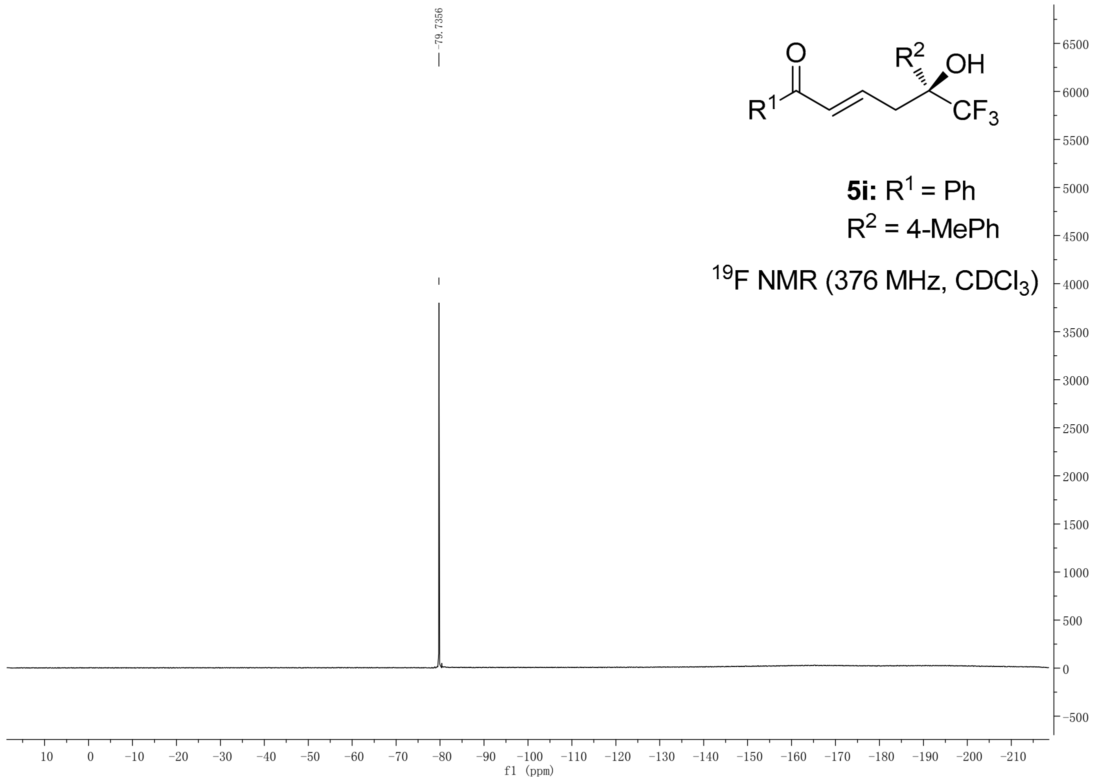

Figure S54 ^{19}F NMR spectrum of **5i.**

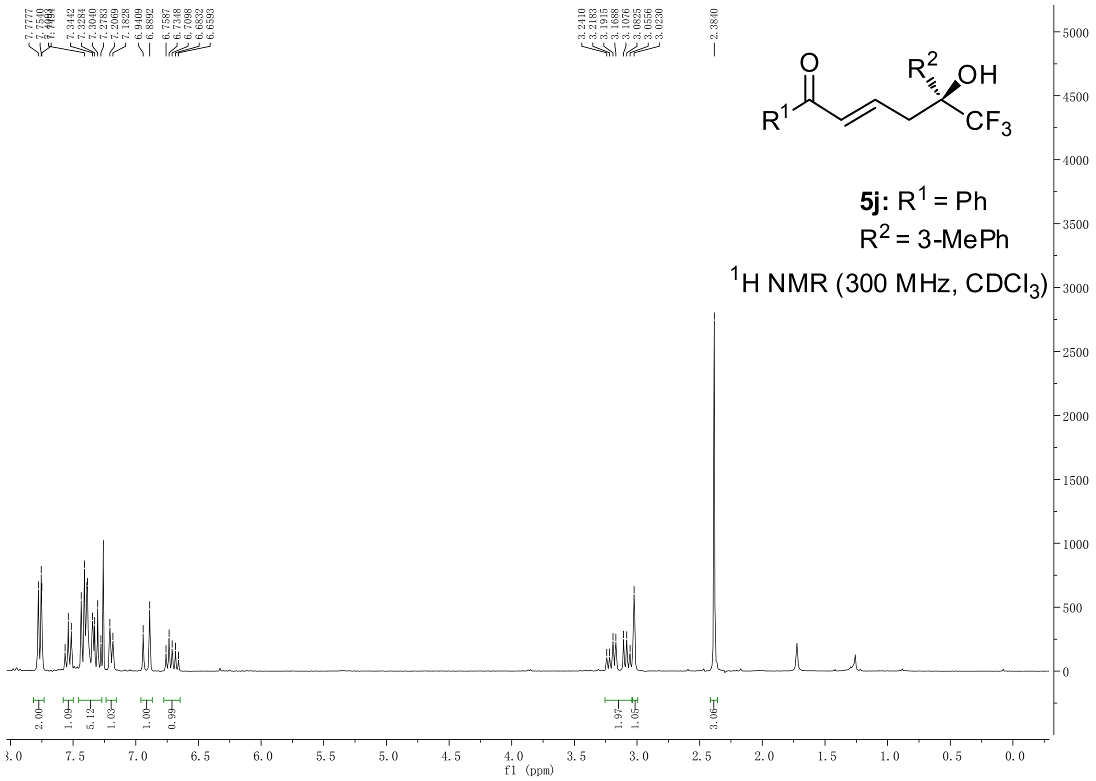

Figure S55 ^{1}H NMR spectrum of **5j.**

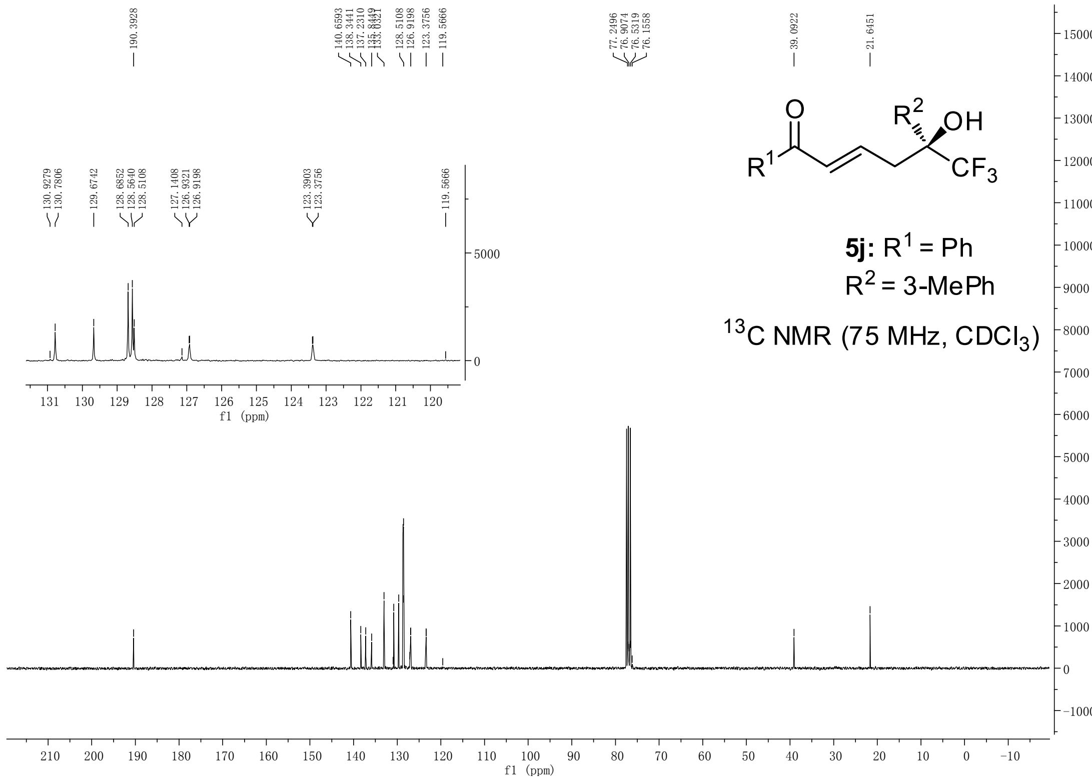

Figure S56 ^{13}C NMR spectrum of **5j.**

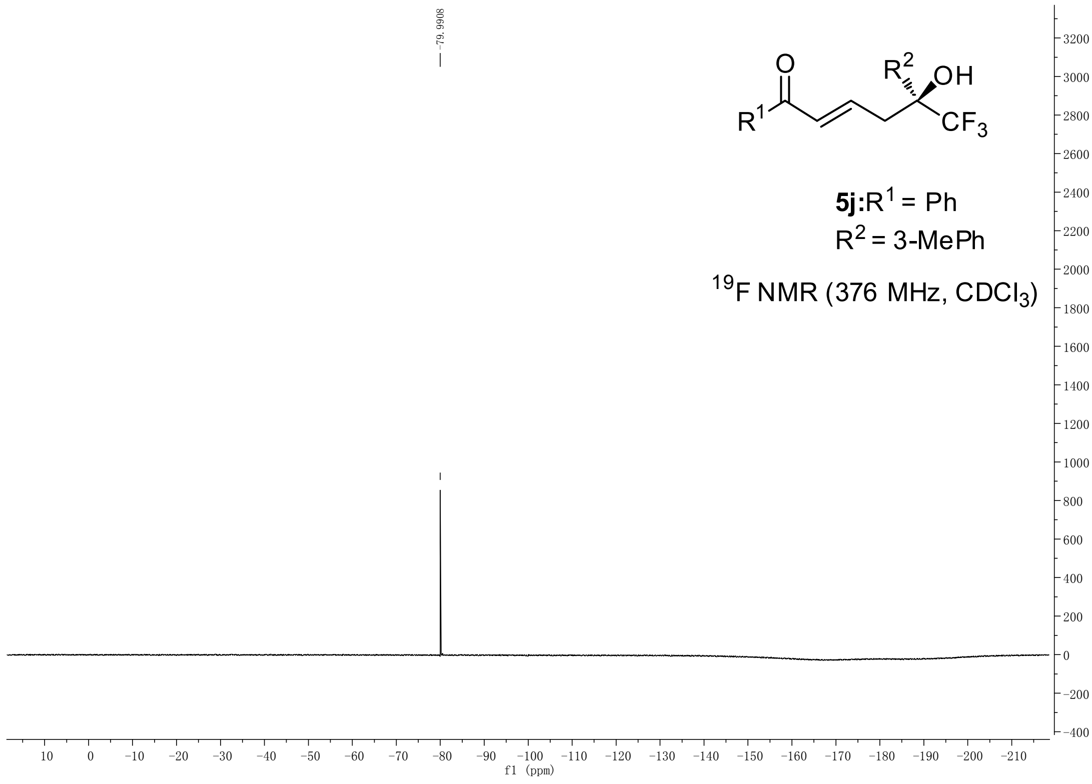

Figure S57 ^{19}F NMR spectrum of **5j.**

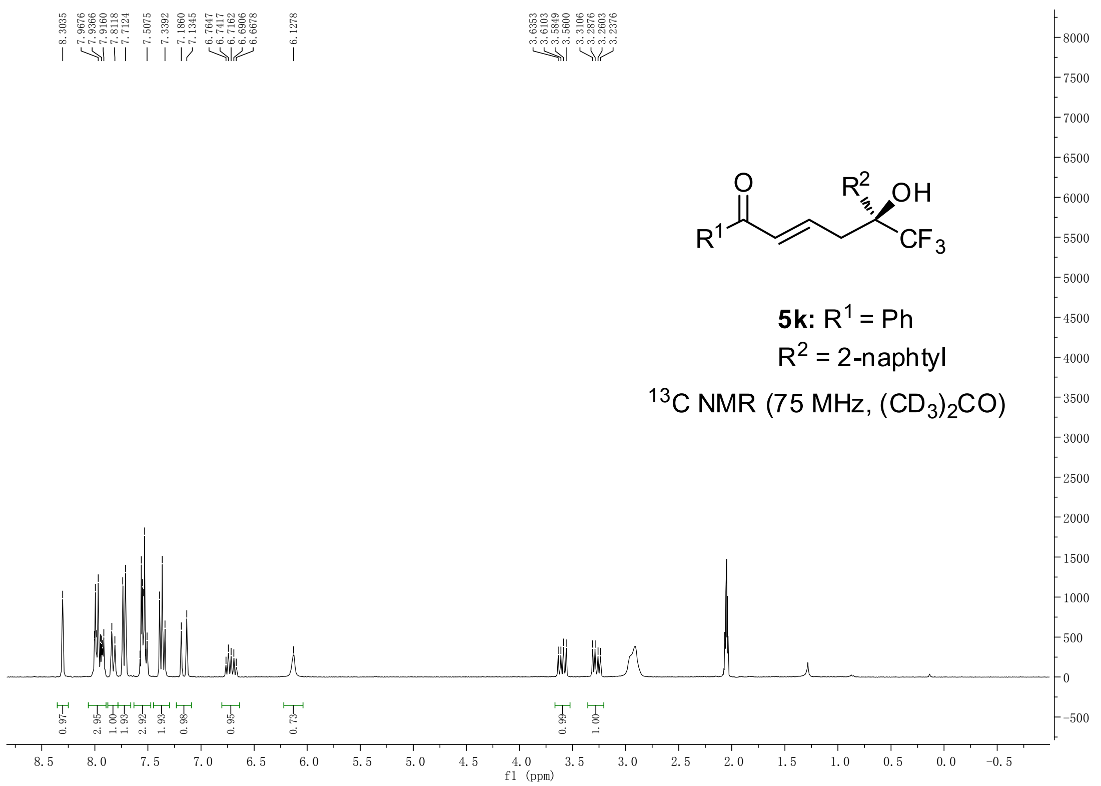

Figure S58 ^{1}H NMR spectrum of **5k.**

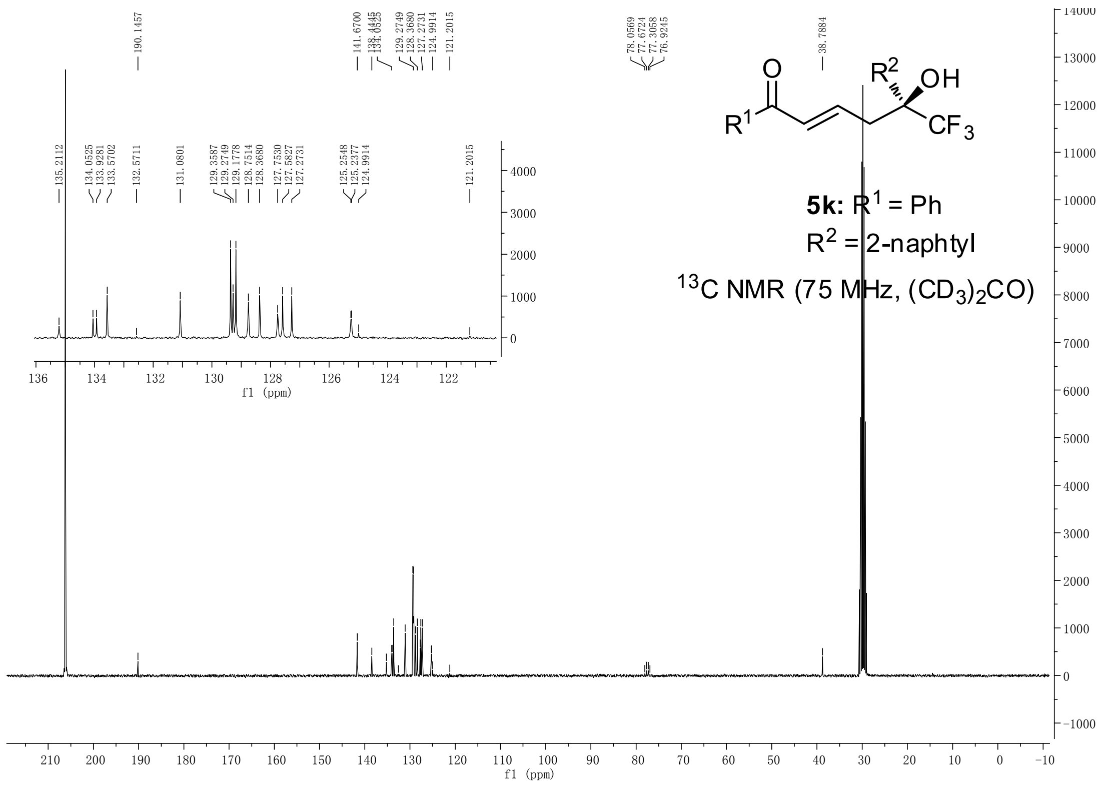

Figure S59 ^{13}C NMR spectrum of **5k.**

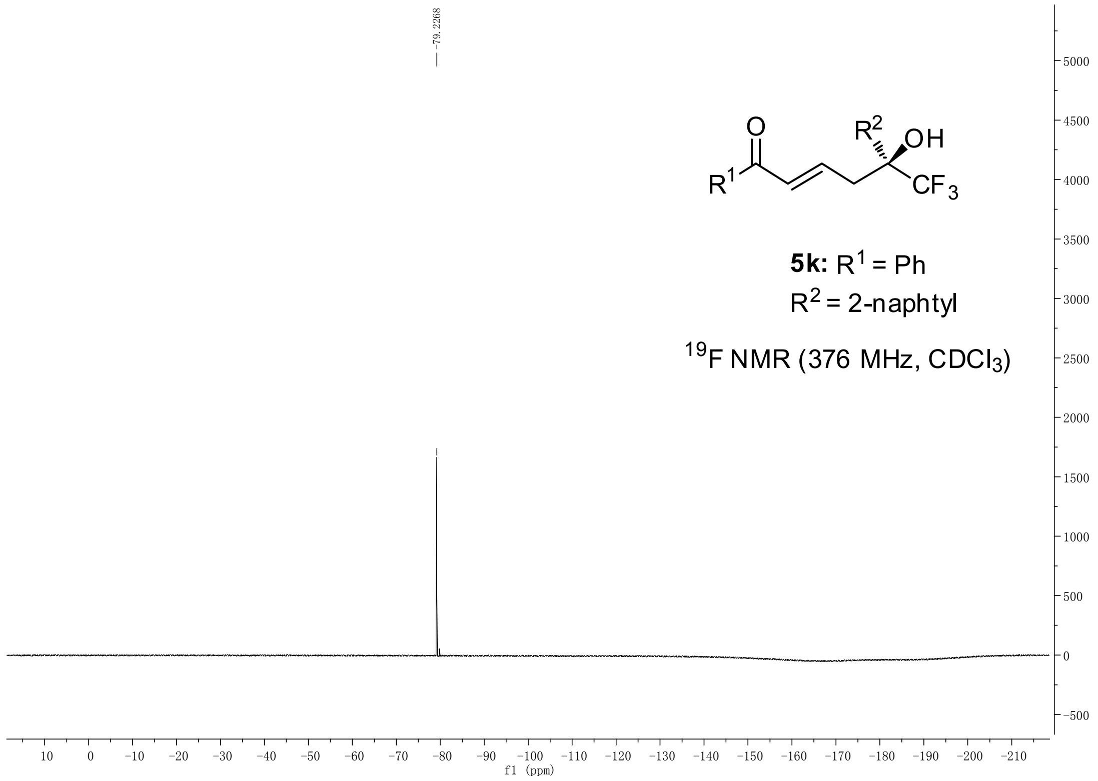

Figure S60 ^{19}F NMR spectrum of **5k.**

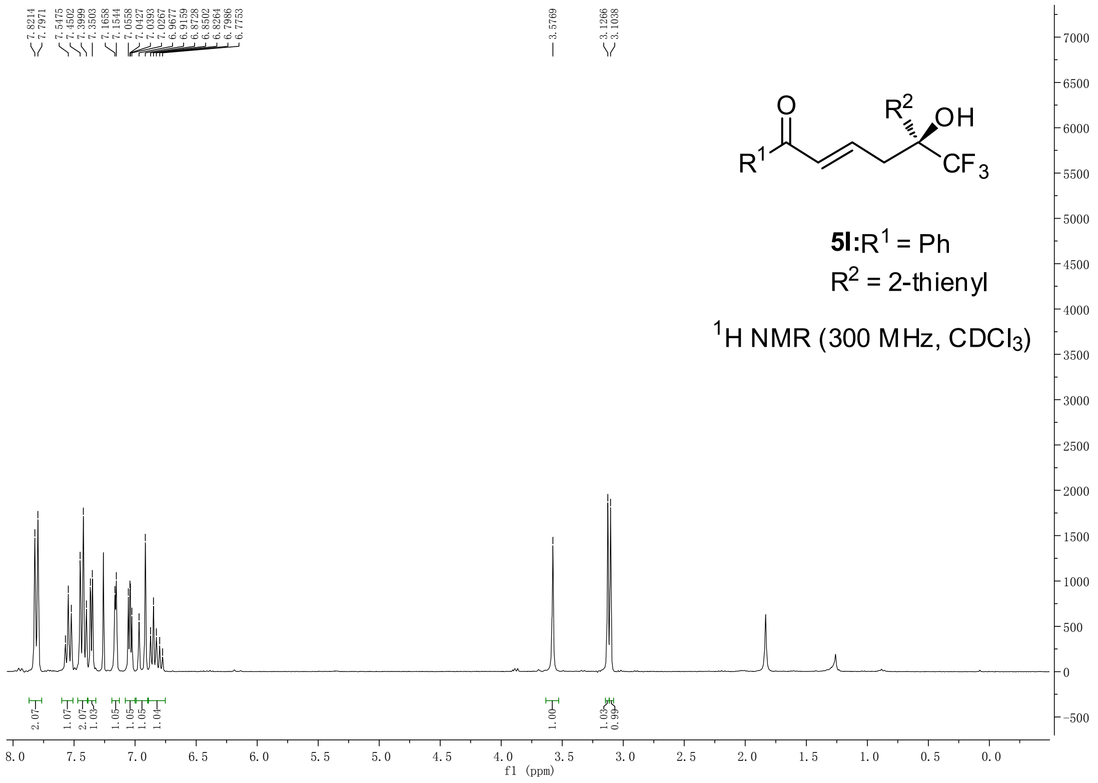

Figure S61 ^{1}H NMR spectrum of **5l.**

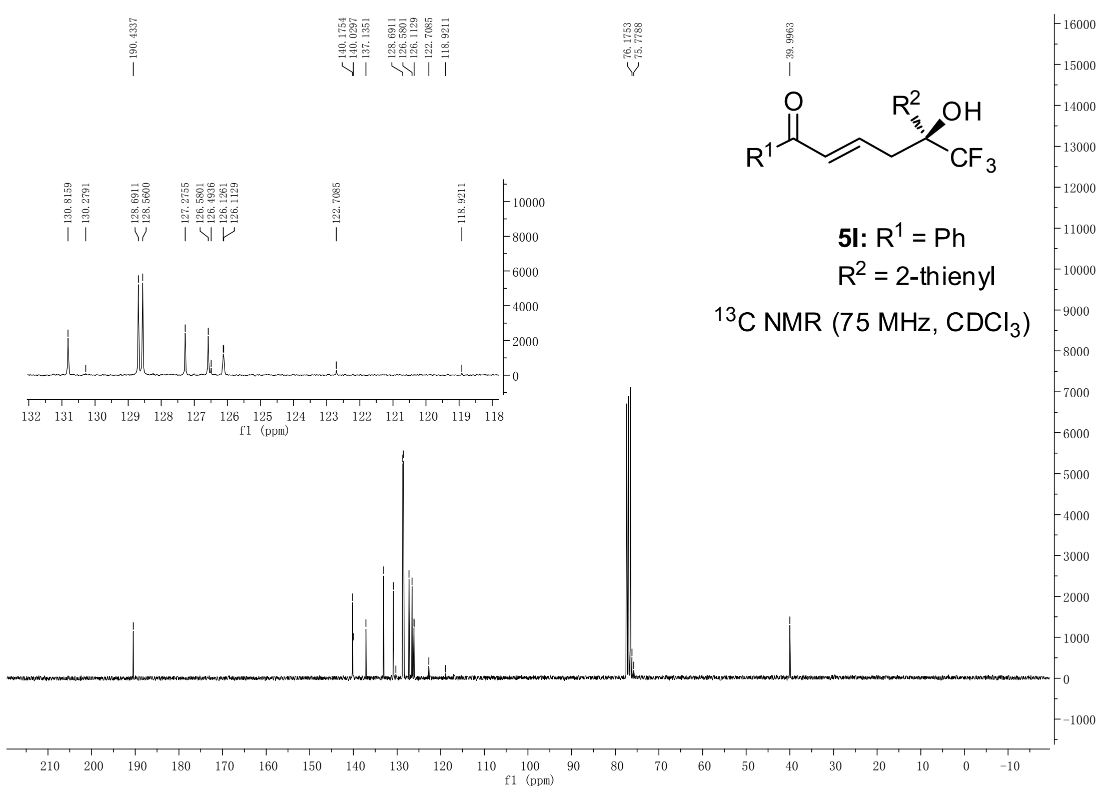

Figure S62 ^{13}C NMR spectrum of **5l.**

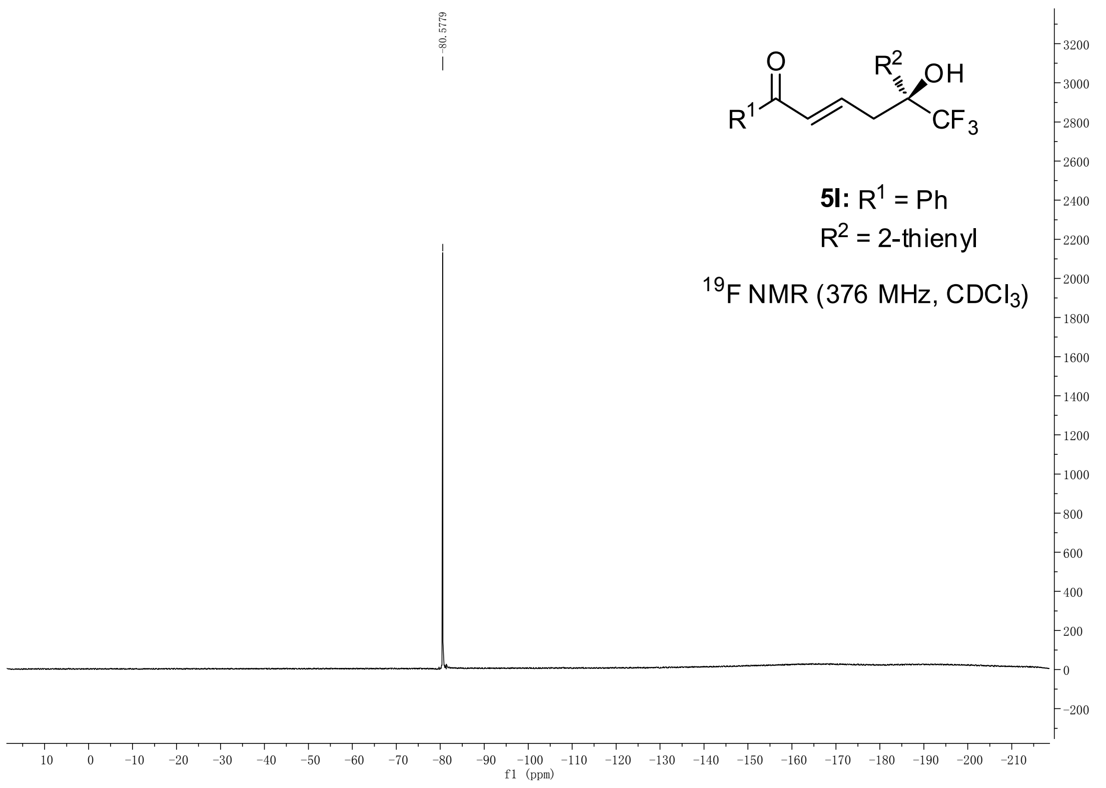

Figure S63 ^{19}F NMR spectrum of **5l.**

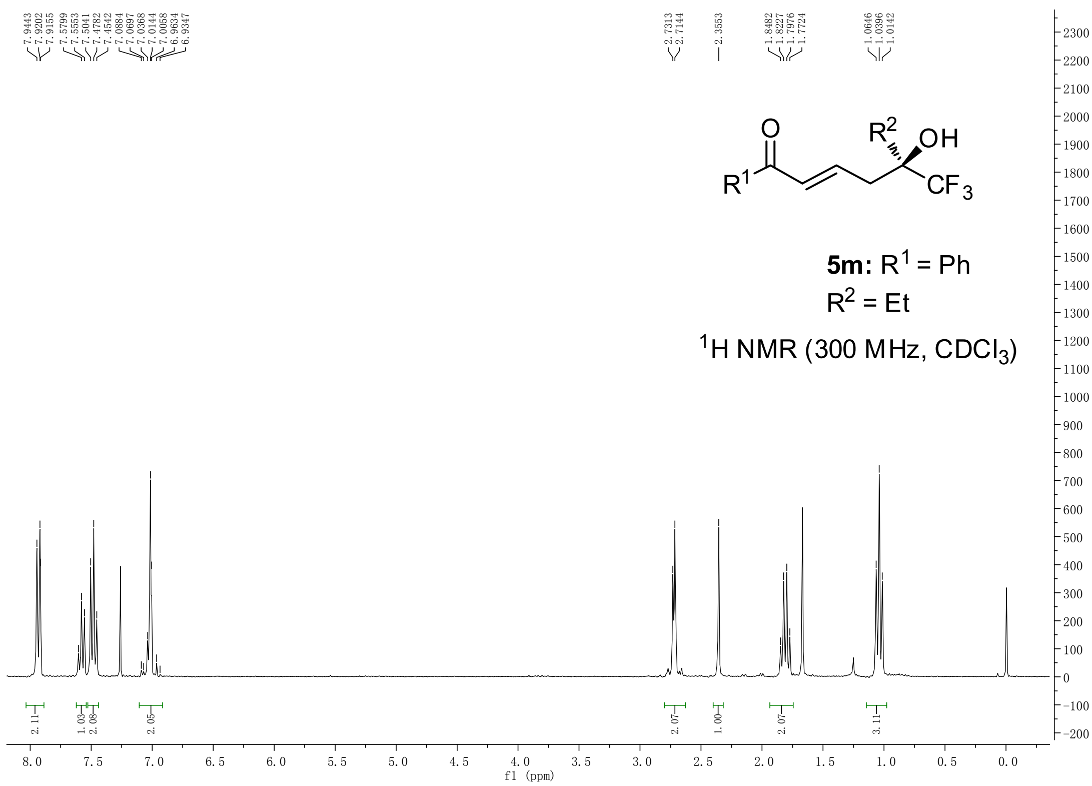

Figure S64 ^{1}H NMR spectrum of **5m.**

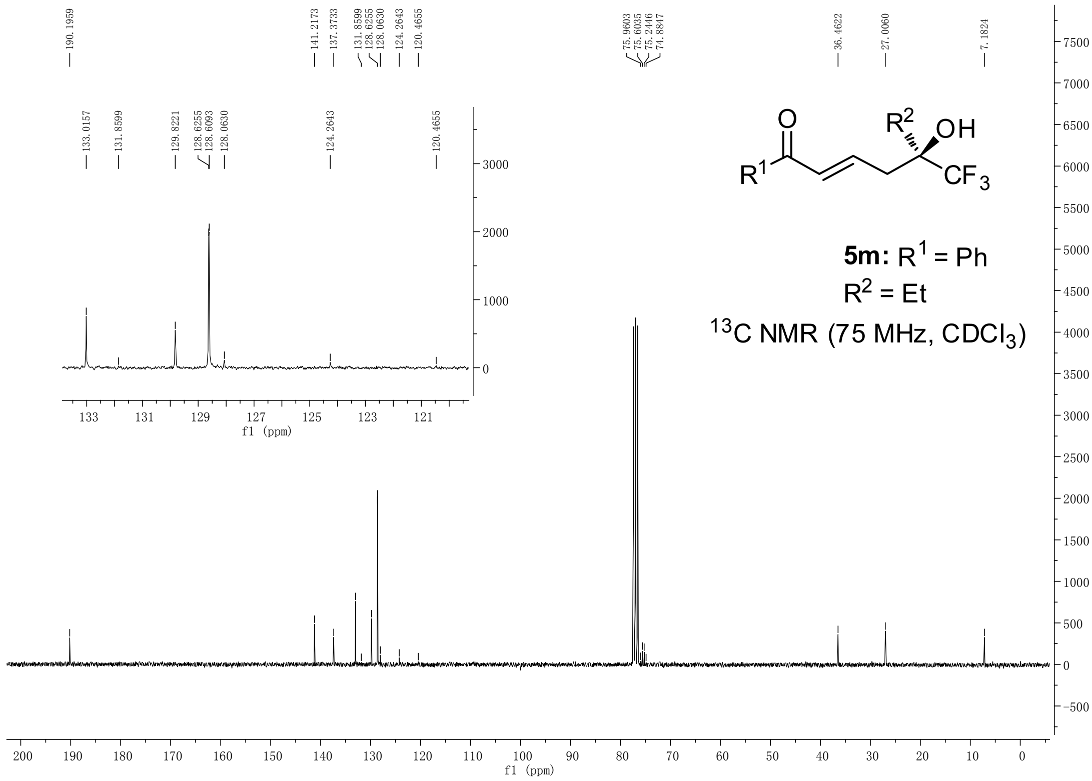

Figure S65 ^{13}C NMR spectrum of **5m.**

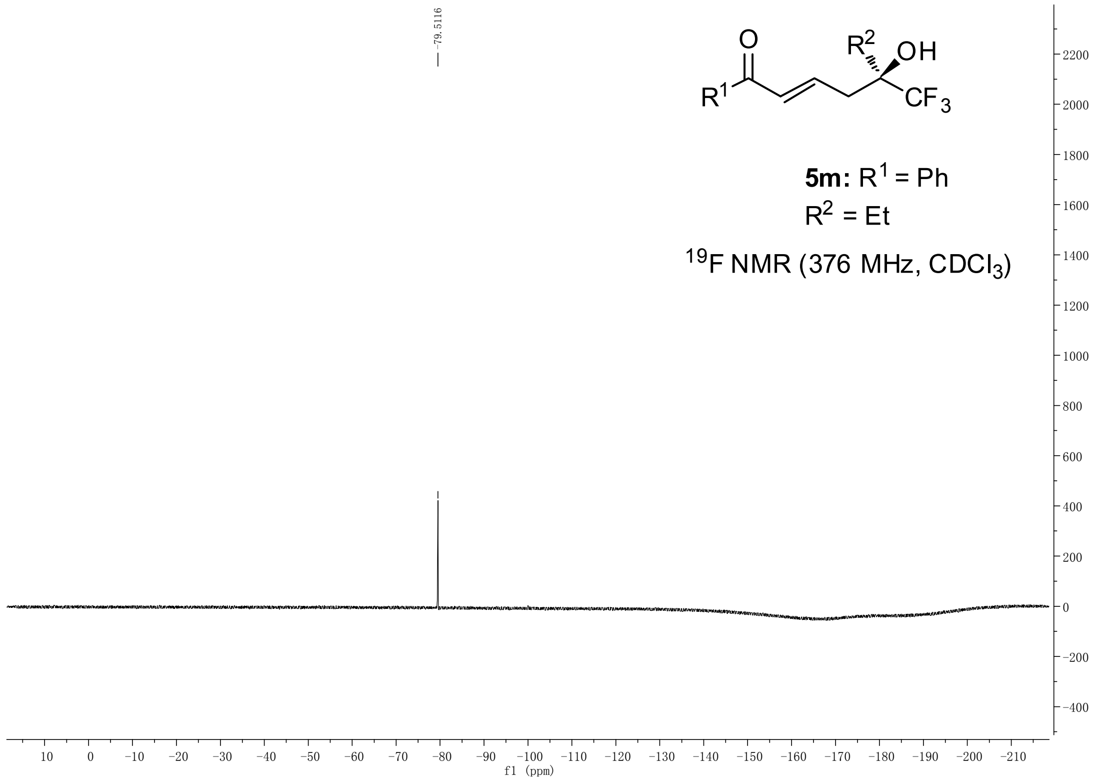

Figure S66 ^{19}F NMR spectrum of **5m.**

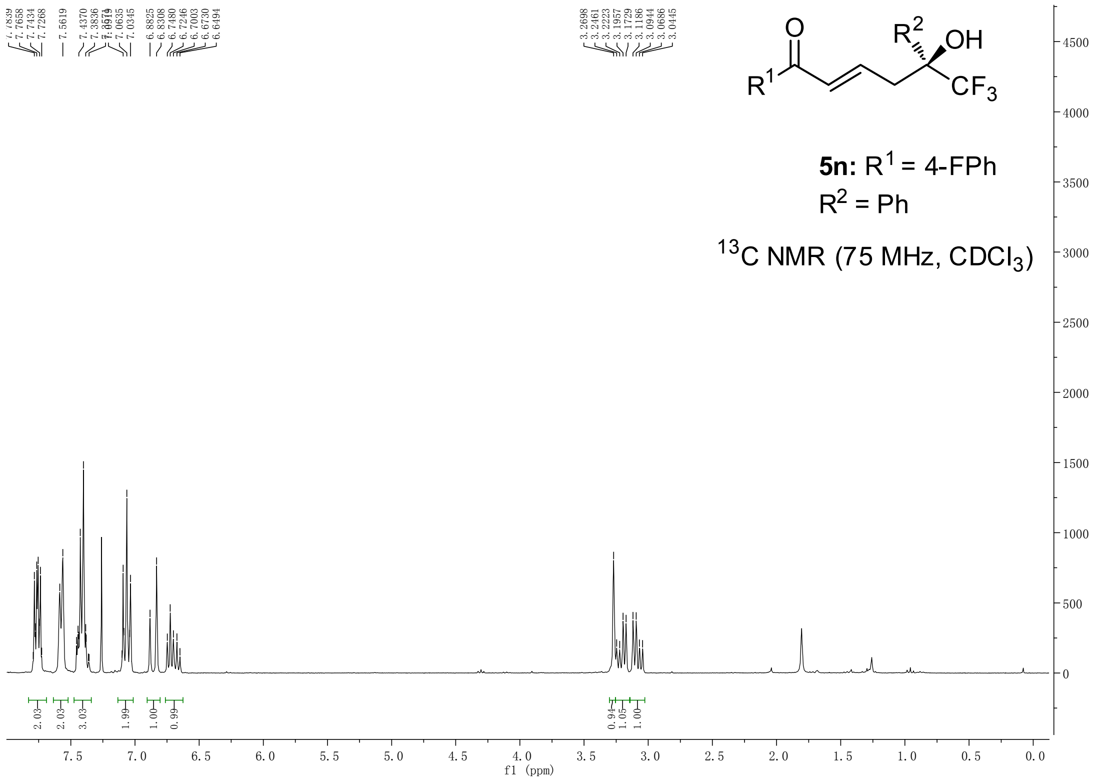

Figure S67 ^{1}H NMR spectrum of **5n.**

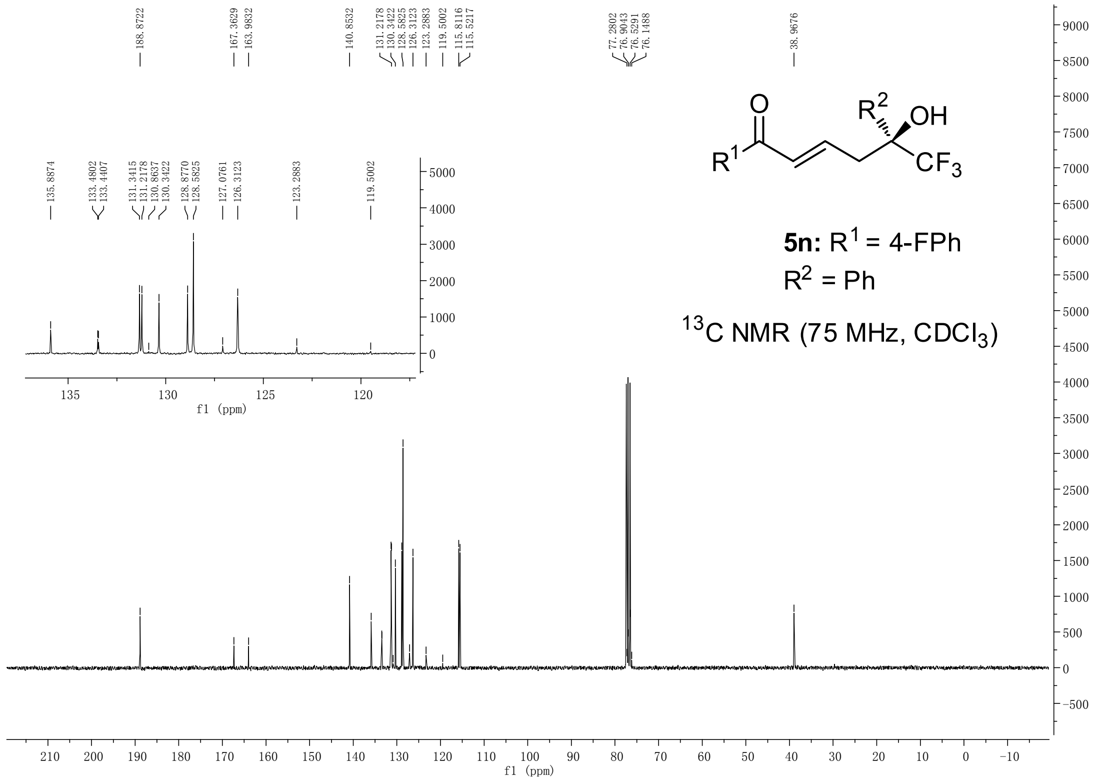

Figure S68 ^{13}C NMR spectrum of **5n.**

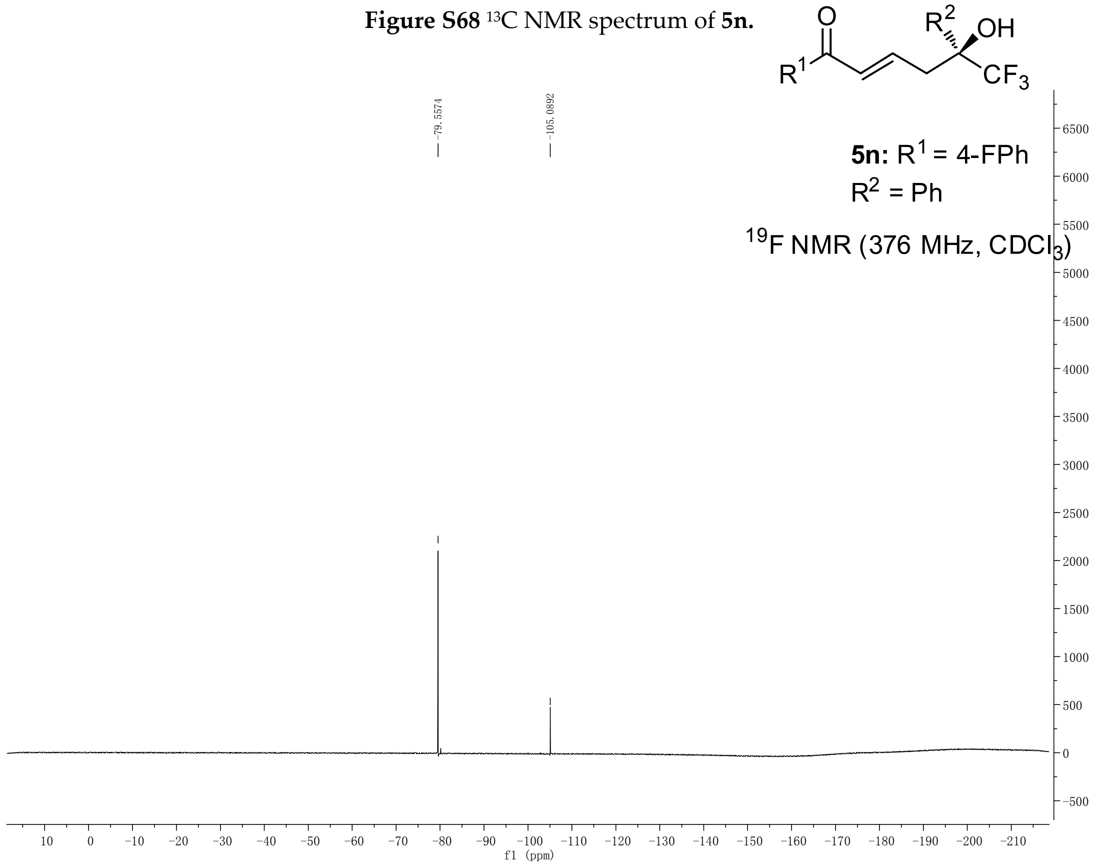

Figure S69 ^{19}F NMR spectrum of **5n.**

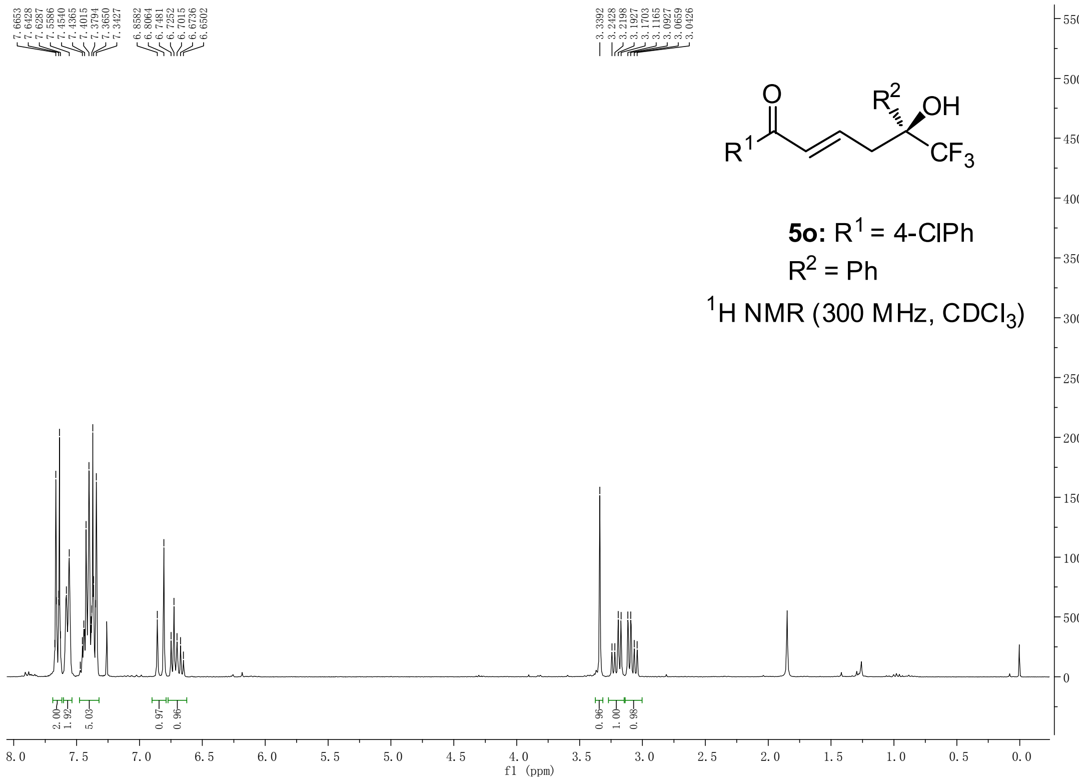

Figure S70 ^{1}H NMR spectrum of **5o.**

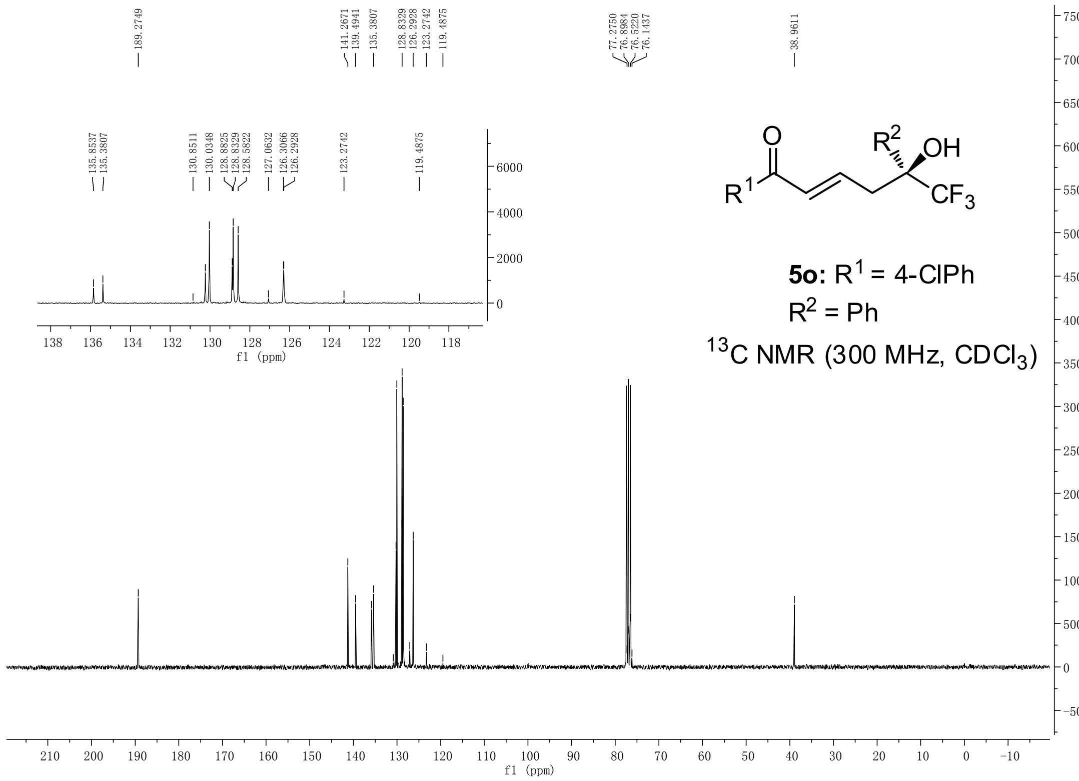

Figure S71 ^{13}C NMR spectrum of **5o.**

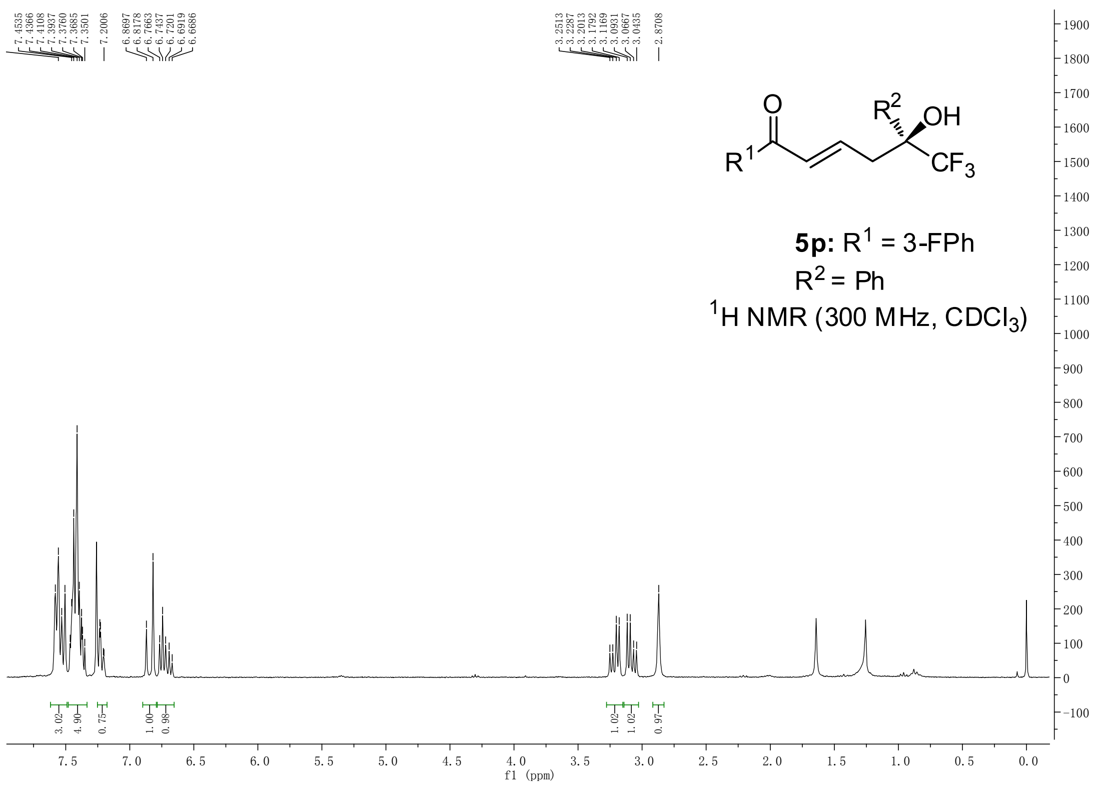

Figure S72 ^{1}H NMR spectrum of **5p.**

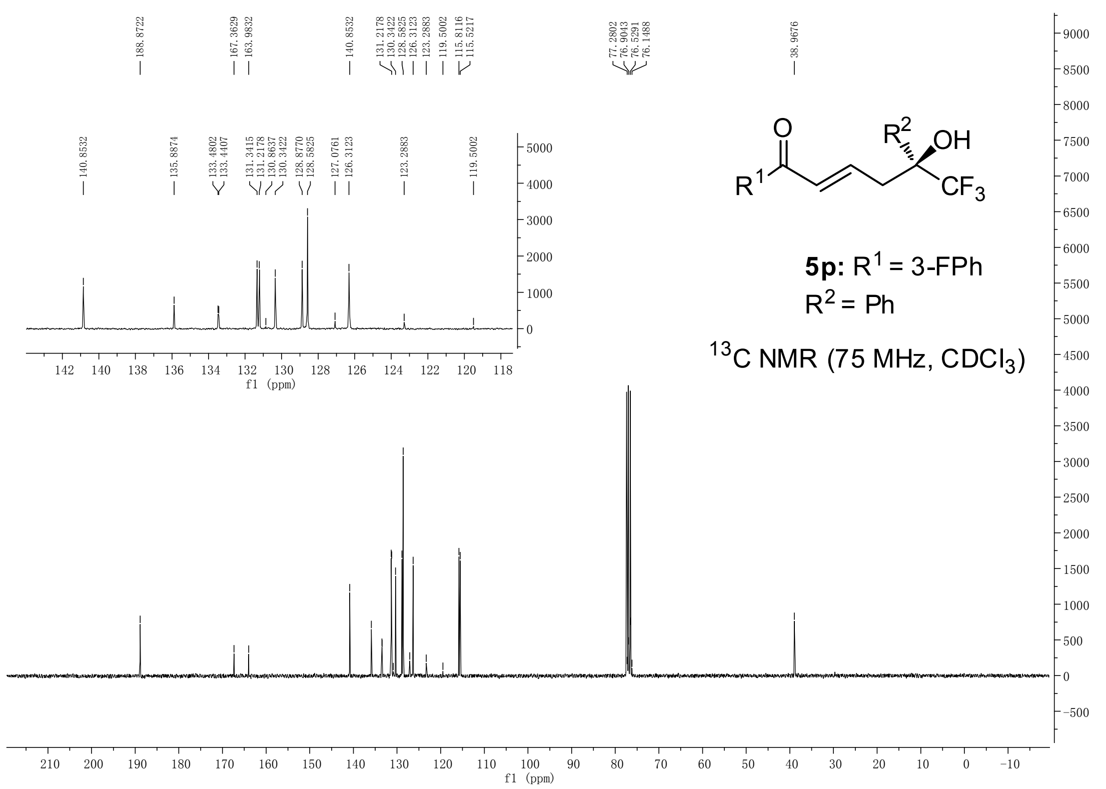

Figure S73 ^{13}C NMR spectrum of **5p.**

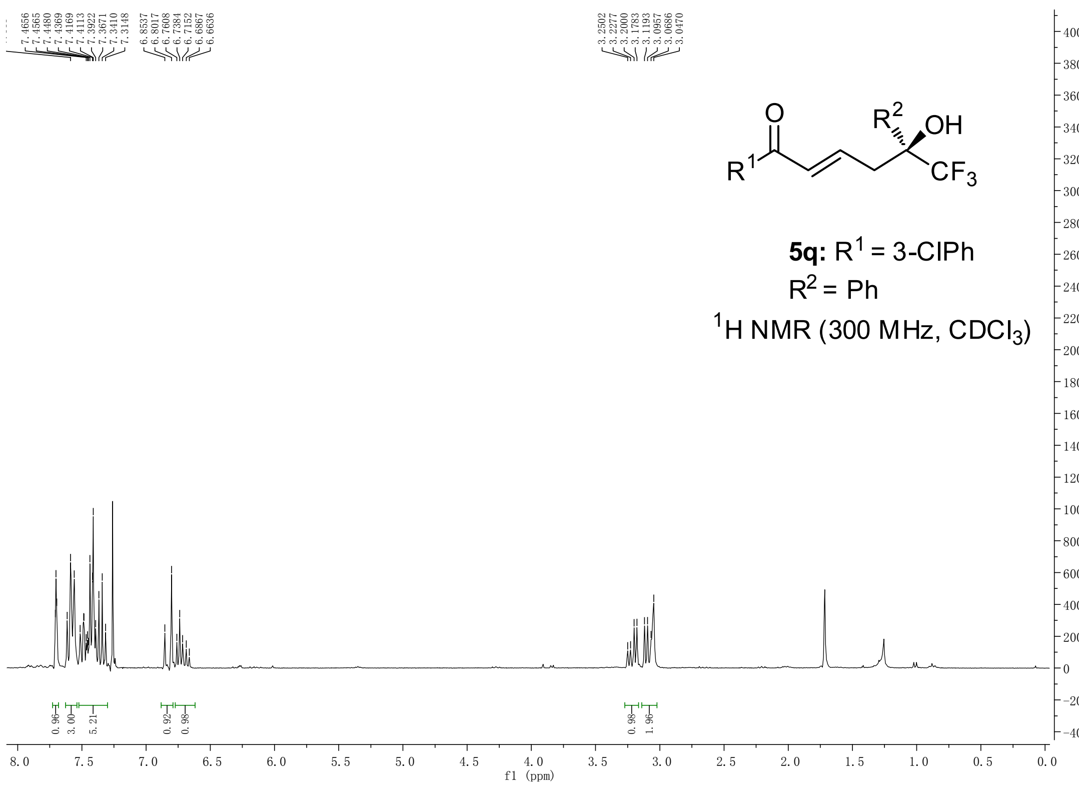

Figure S74 ^{1}H NMR spectrum of **5q.**

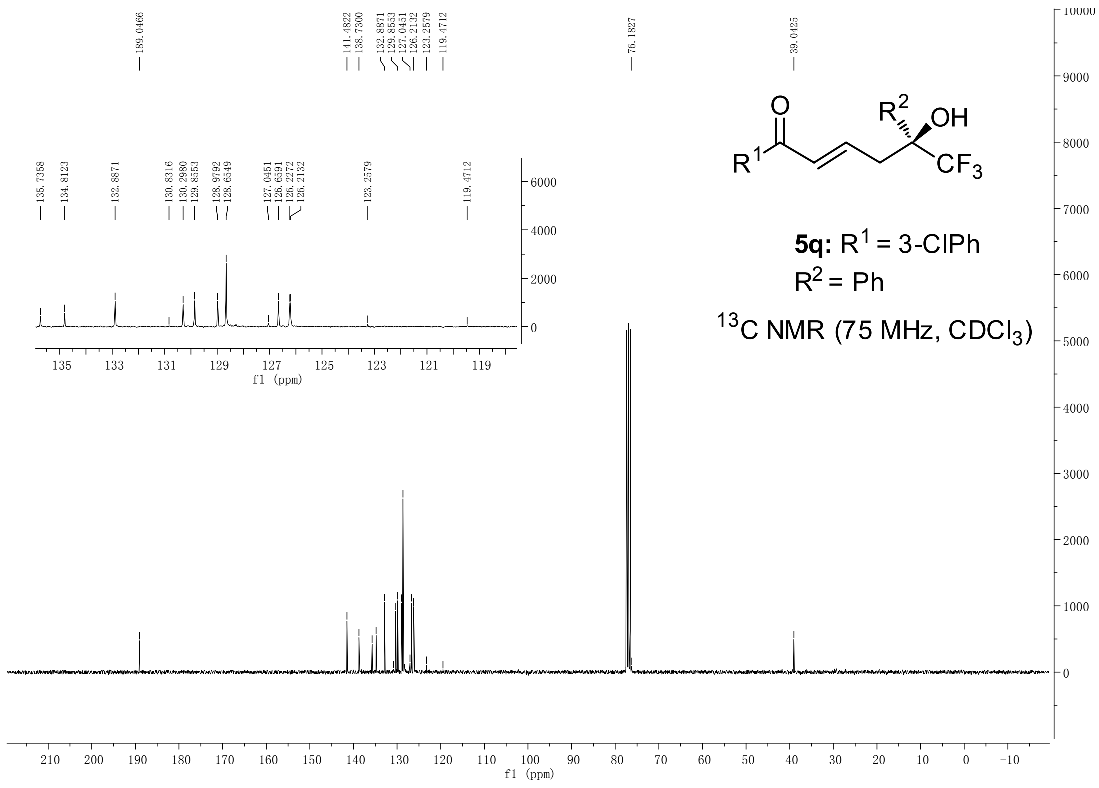

Figure S75 ^{13}C NMR spectrum of **5q.**

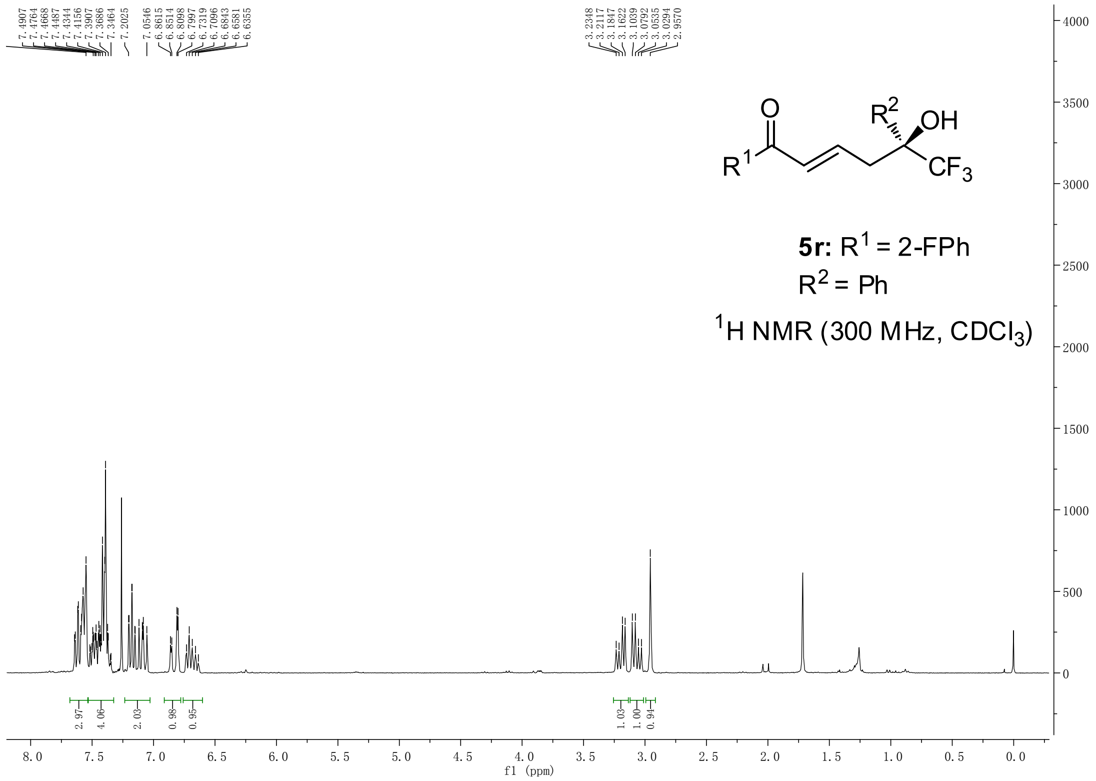

Figure S76 ^{1}H NMR spectrum of **5r.**

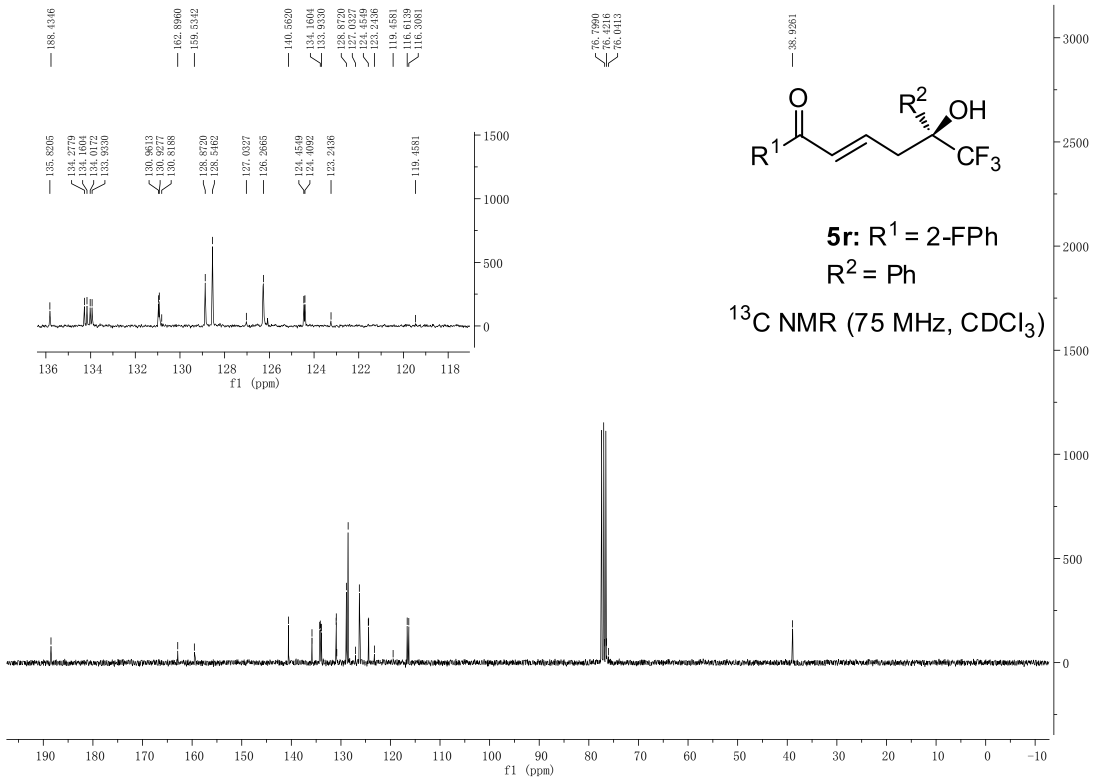

Figure S77 ^{13}C NMR spectrum of **5r.**

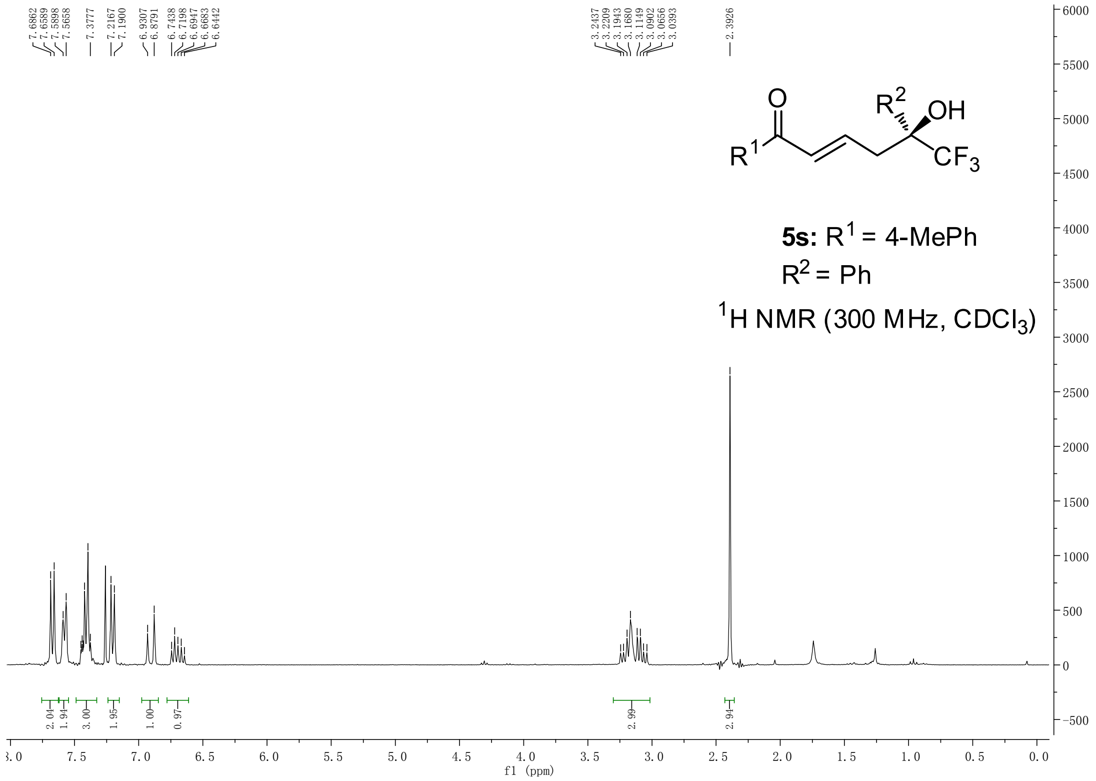

Figure S78 ^{1}H NMR spectrum of **5s.**

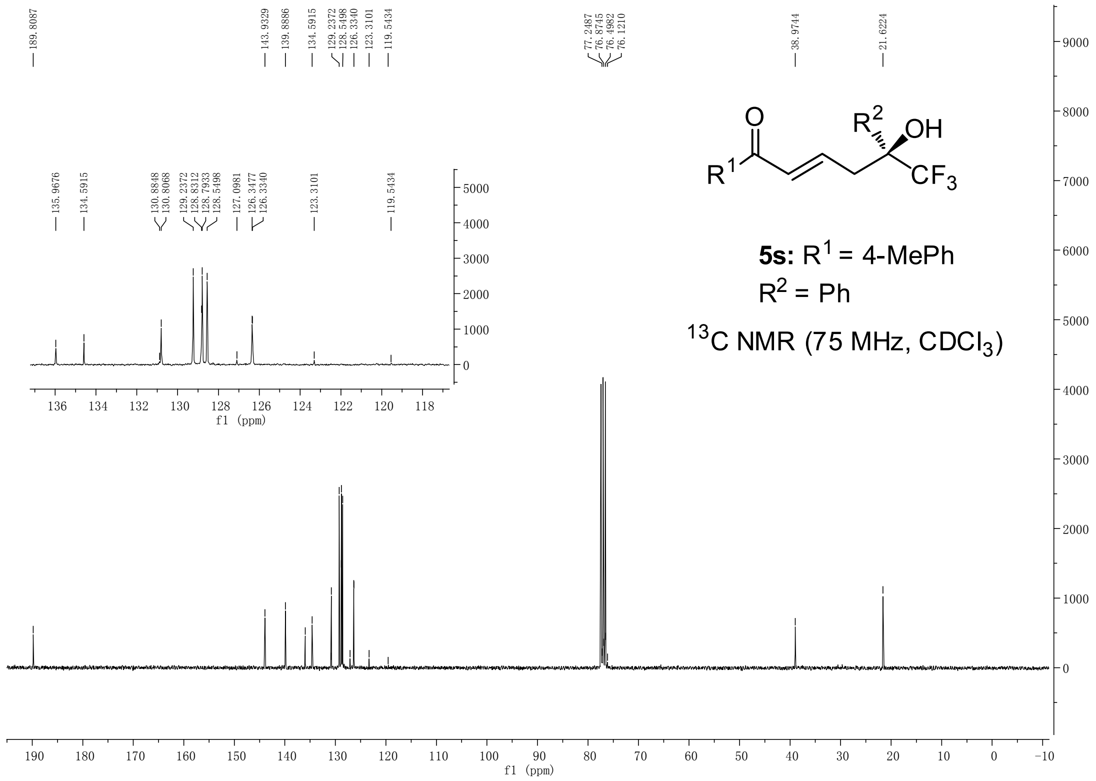

Figure S79 ^{13}C NMR spectrum of **5s.**

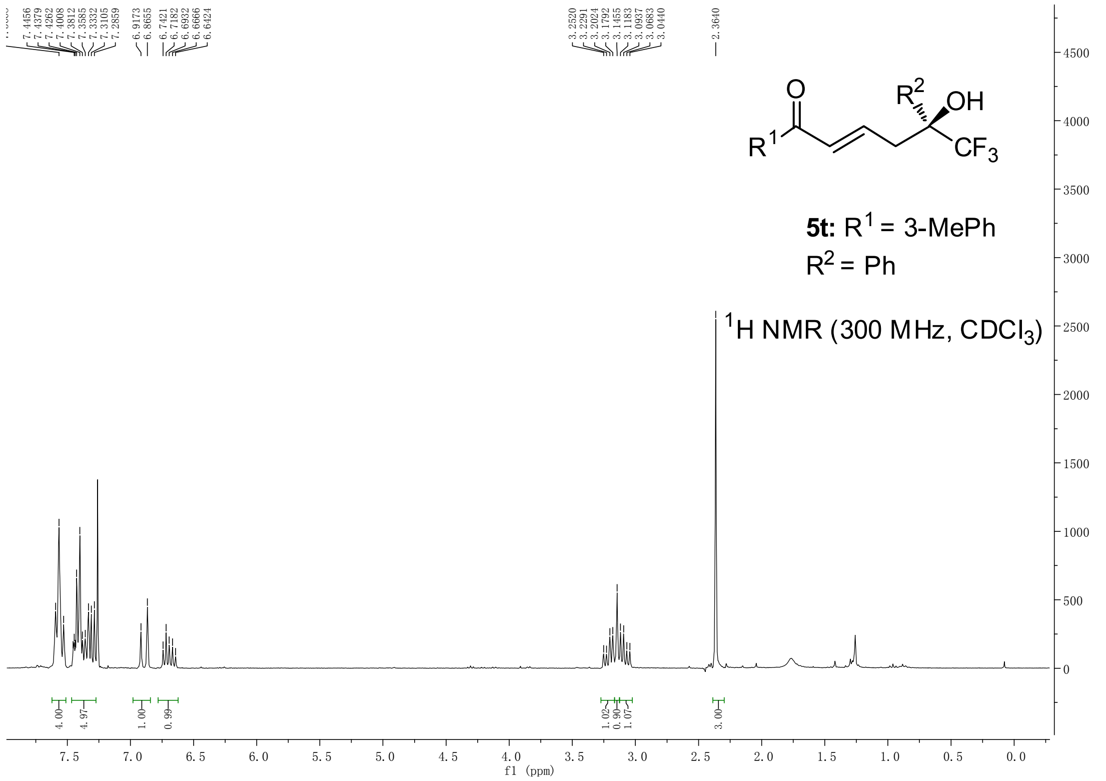

Figure S80 ^{1}H NMR spectrum of **5t.**

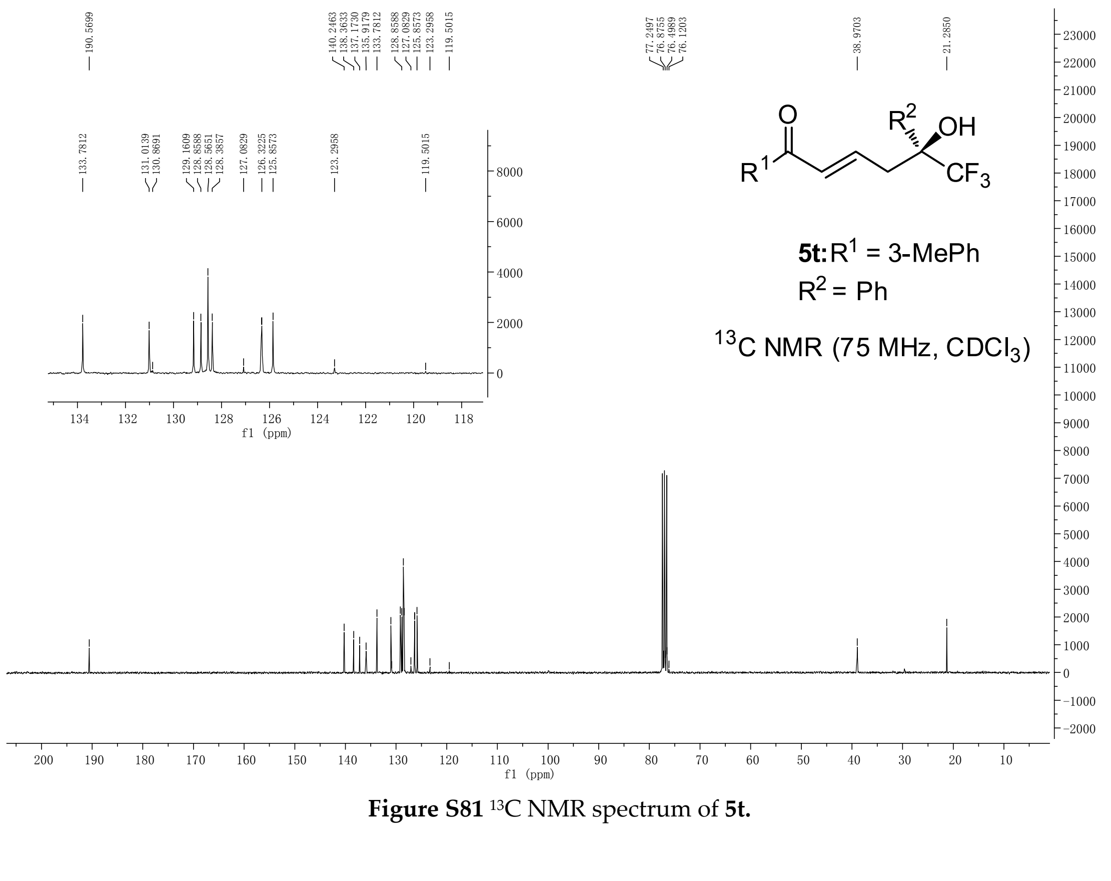

Figure S81 ^{13}C NMR spectrum of **5t.**

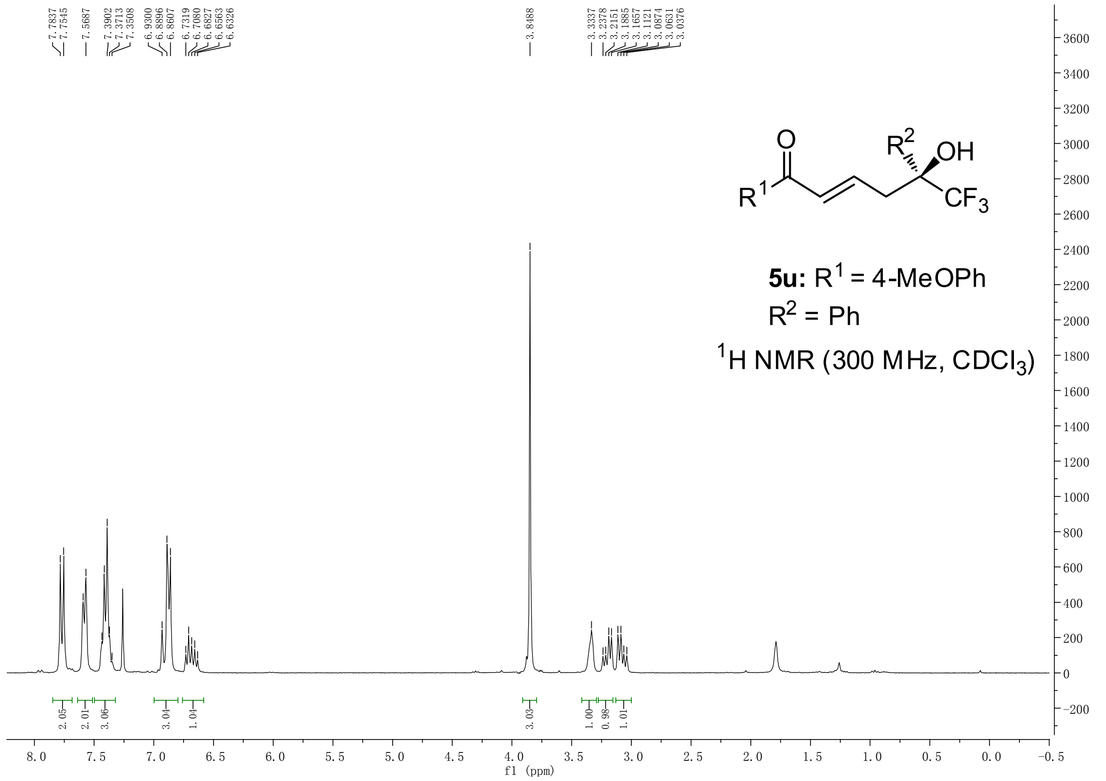

Figure S82 ^{1}H NMR spectrum of **5u.**

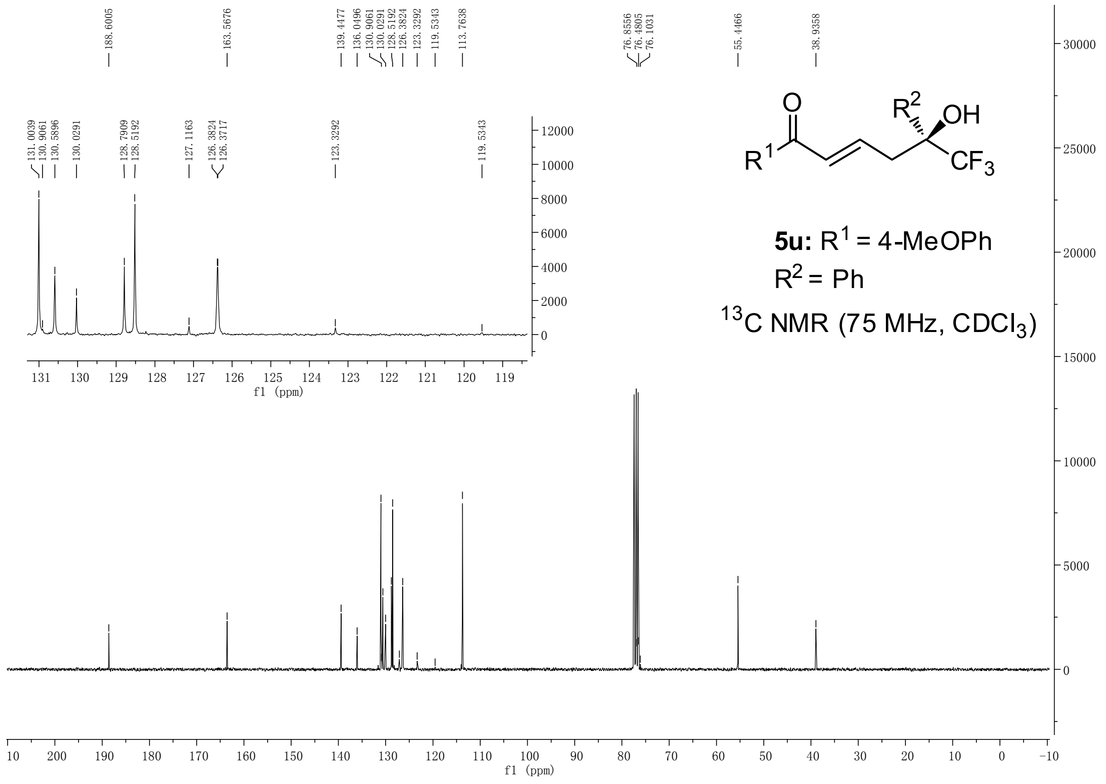

Figure S83 ^{13}C NMR spectrum of **5u.**

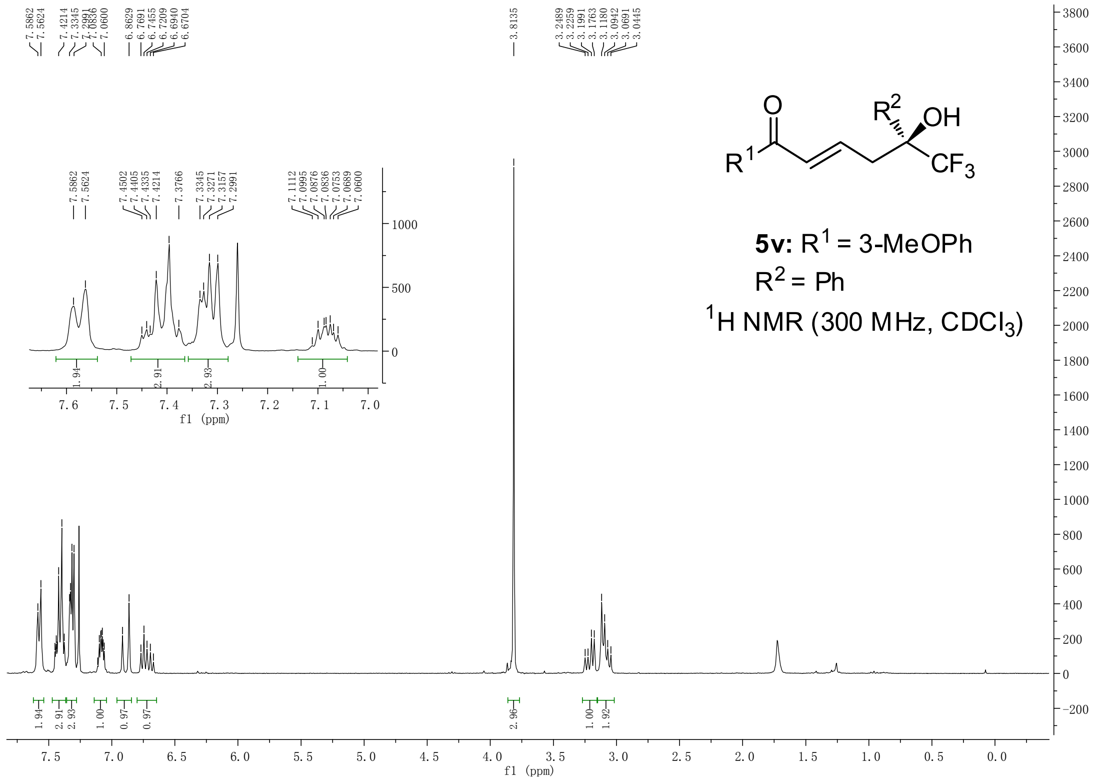

Figure S84 ^{1}H NMR spectrum of **5v.**

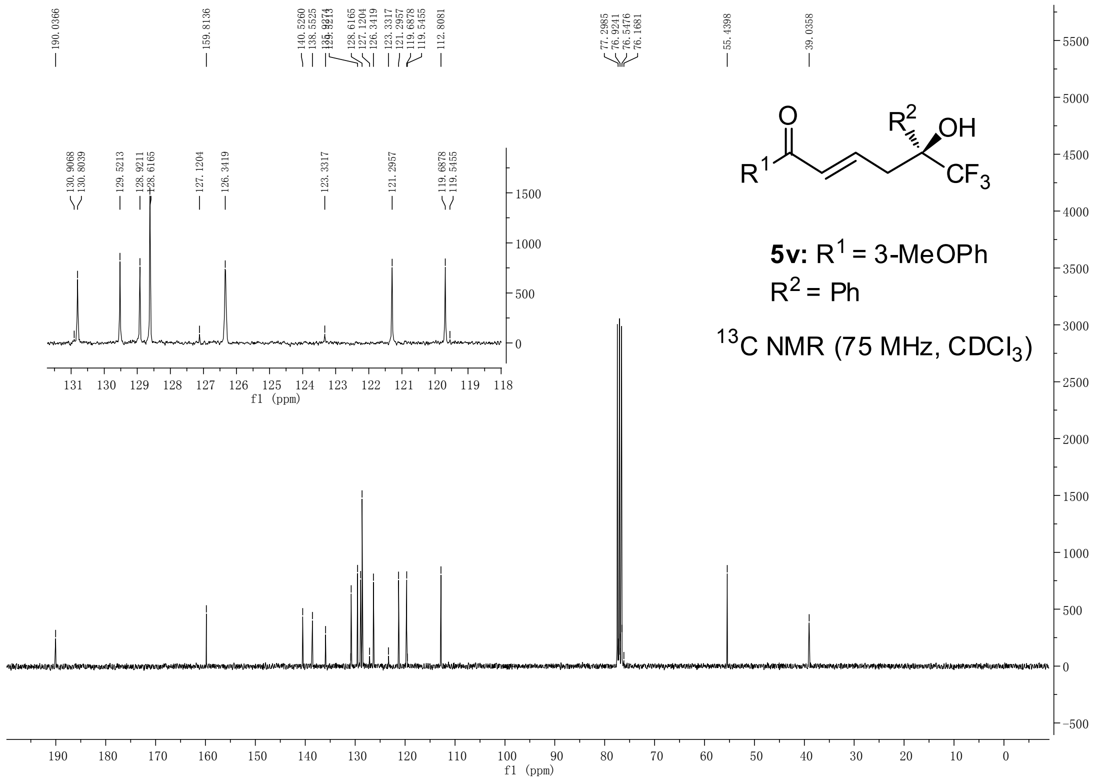

Figure S85 ^{13}C NMR spectrum of **5v.**

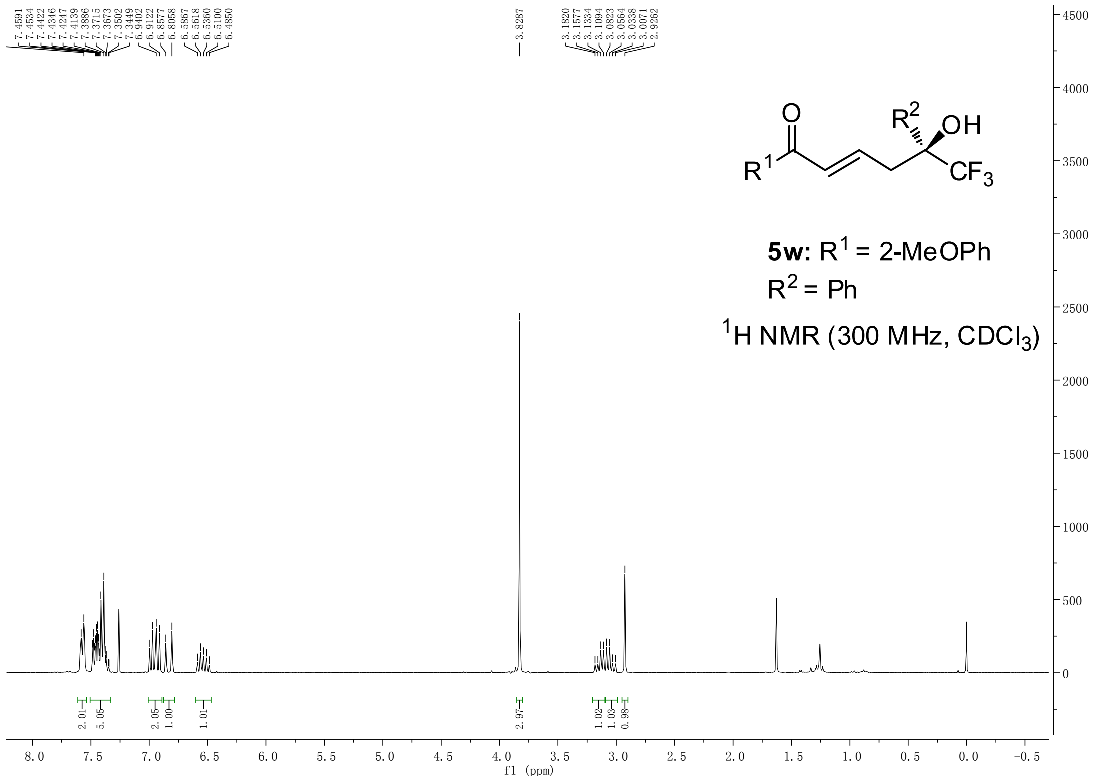

Figure S86 ^{1}H NMR spectrum of **5w.**

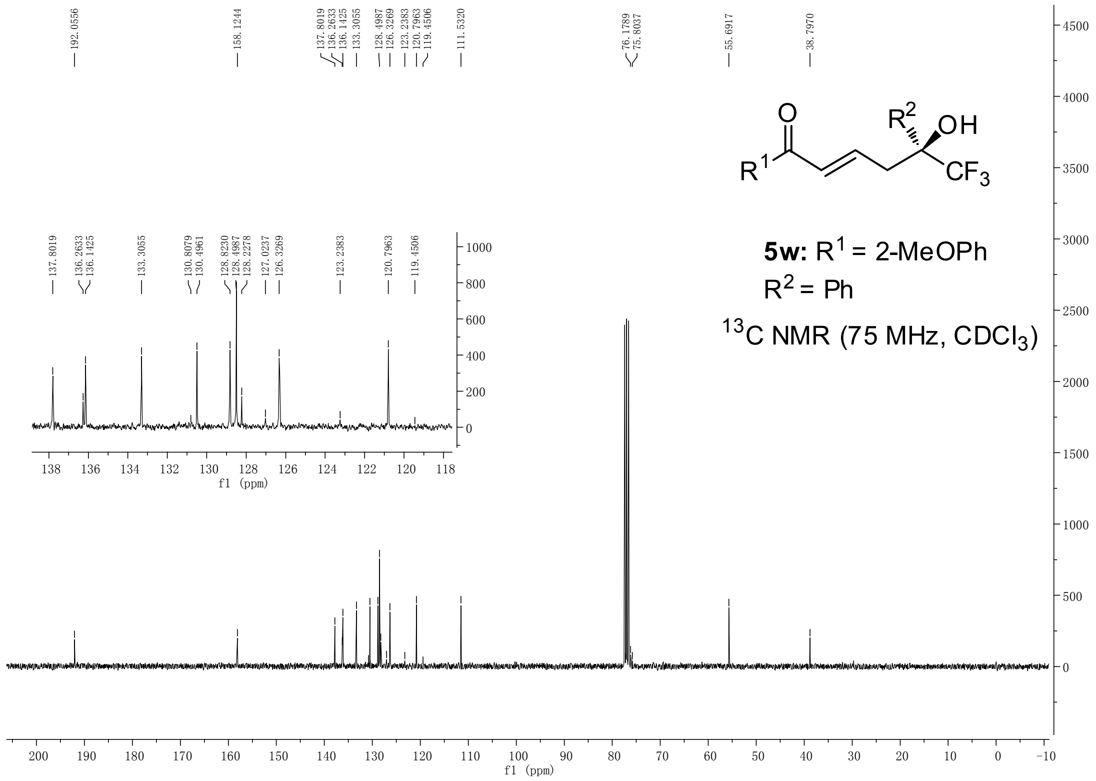

Figure S87 ^{13}C NMR spectrum of **5w.**

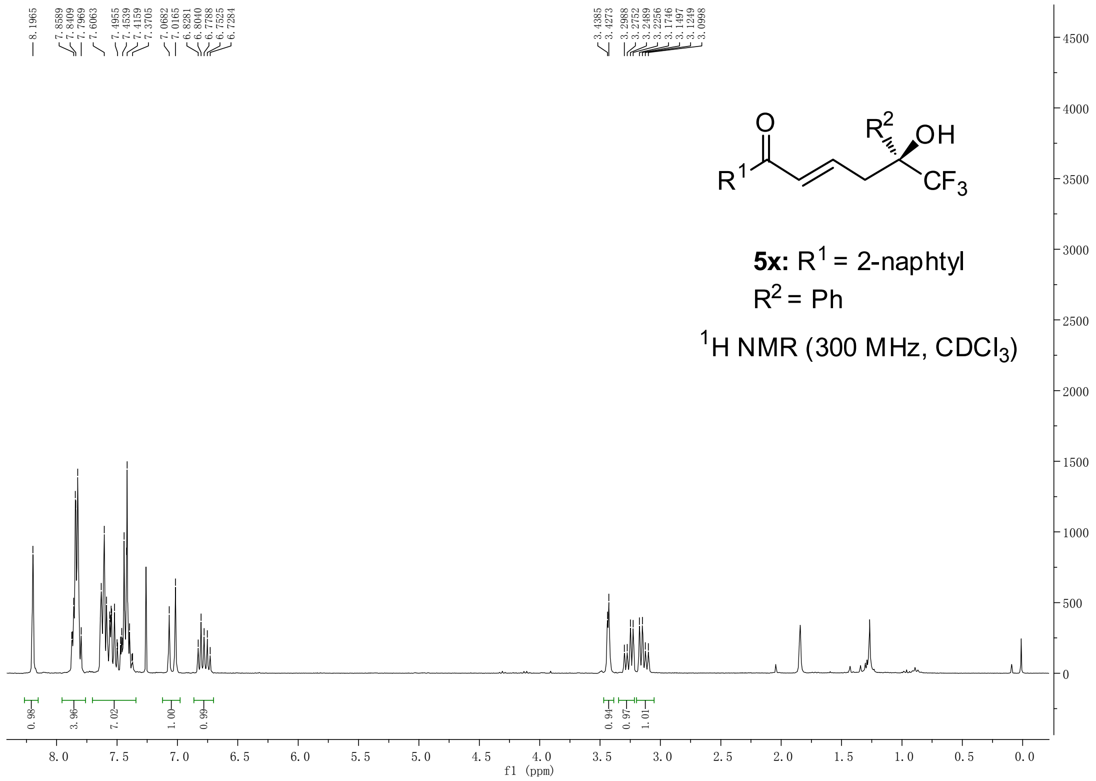

Figure S88 ^{1}H NMR spectrum of **5x.**

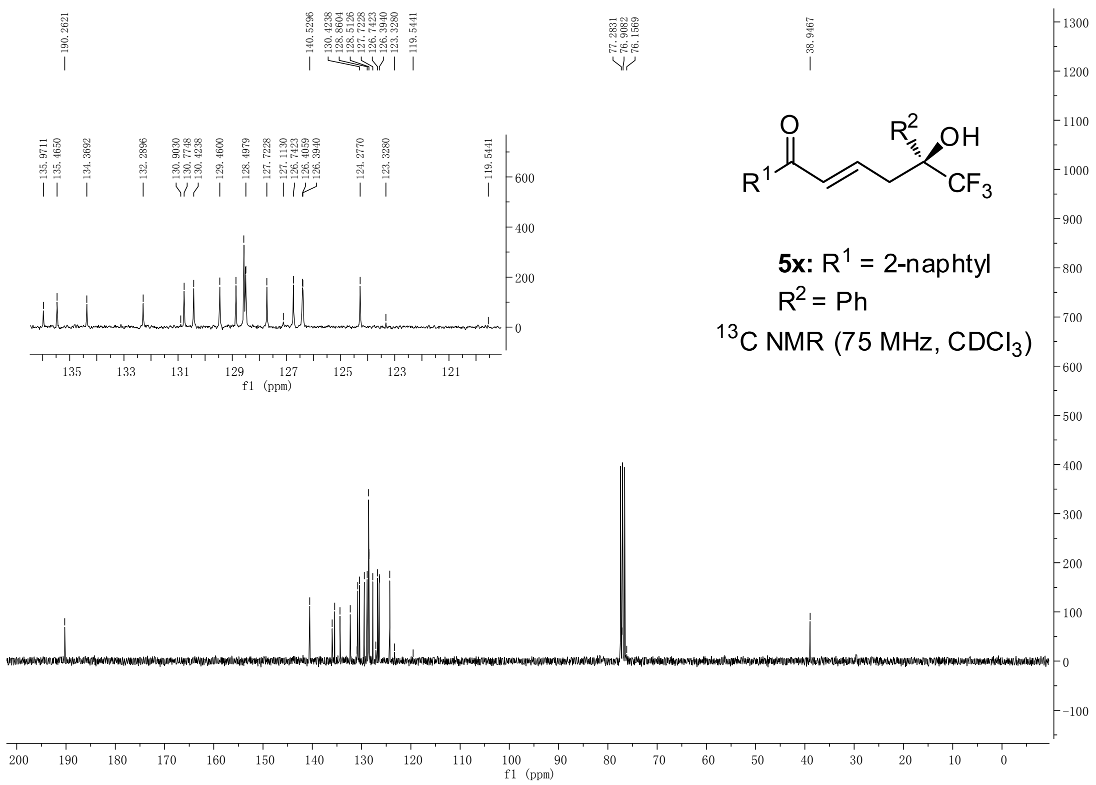

Figure S89 ^{13}C NMR spectrum of **5x.**

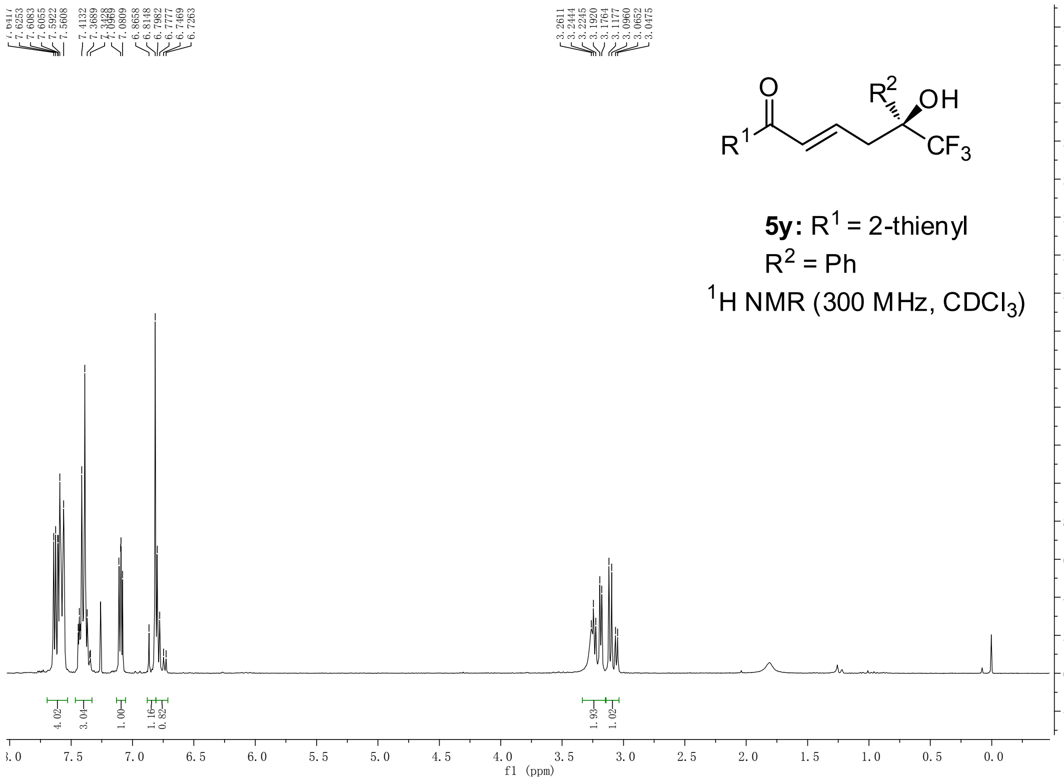

Figure S90 ¹H NMR spectrum of **5y.**

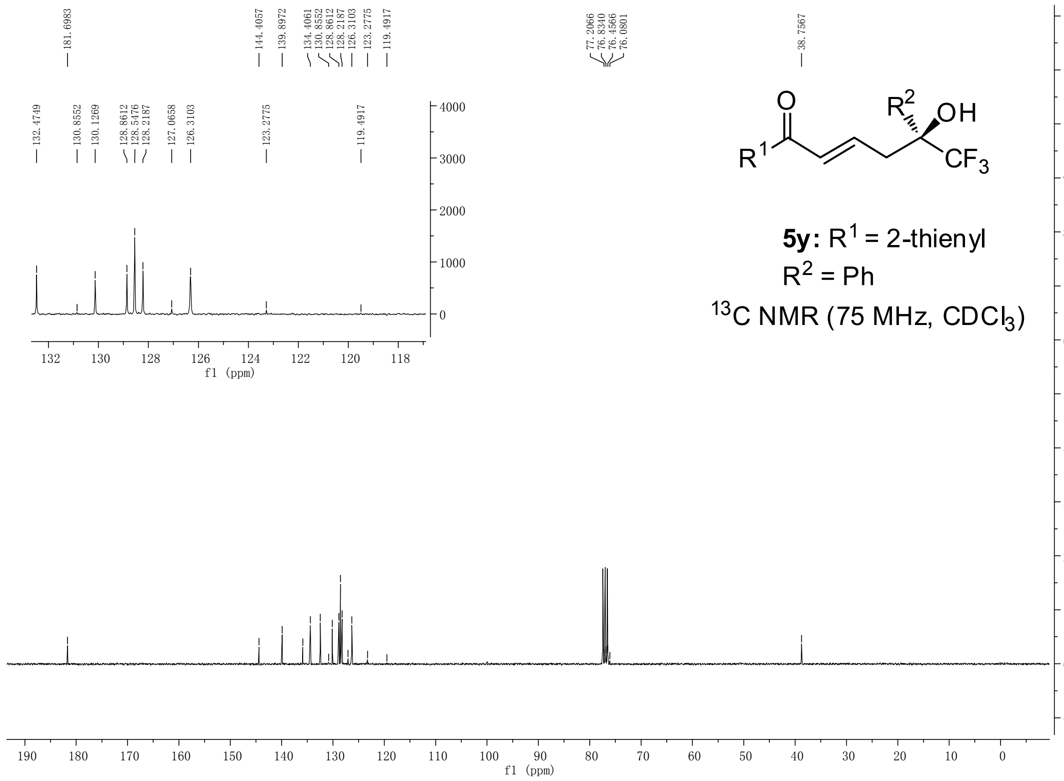

Figure S91 ¹³C NMR spectrum of **5y.**

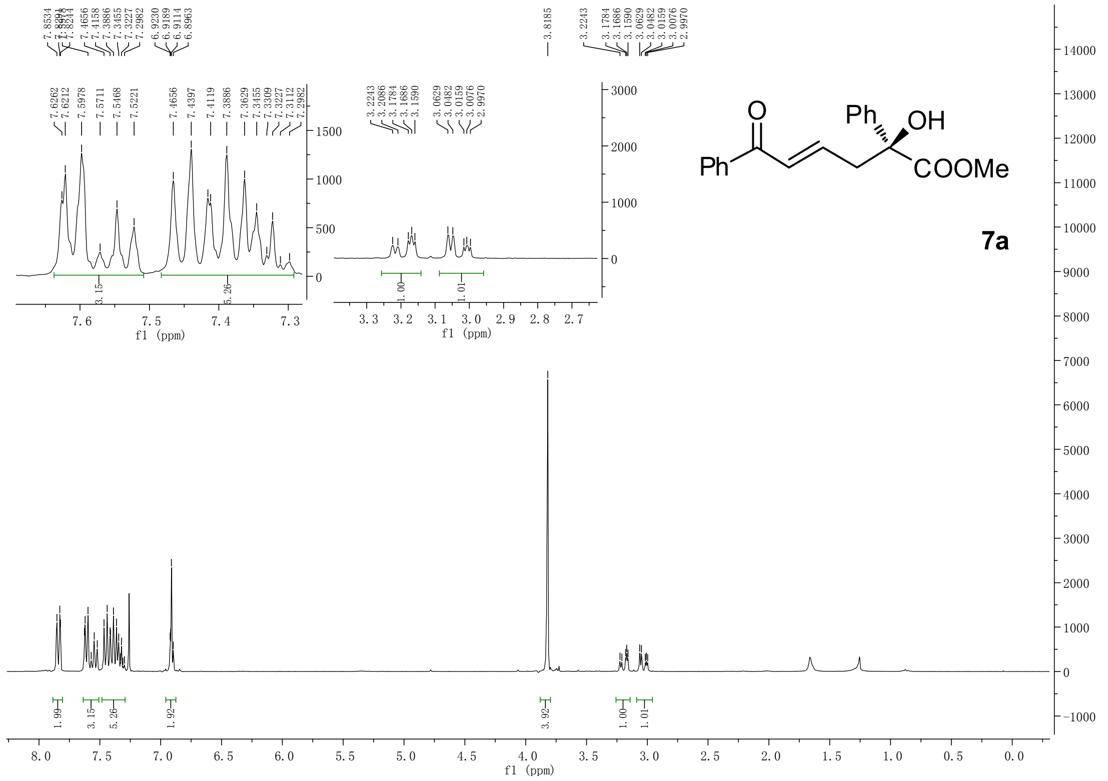

Figure S92 ^{1}H NMR spectrum of **7a.**

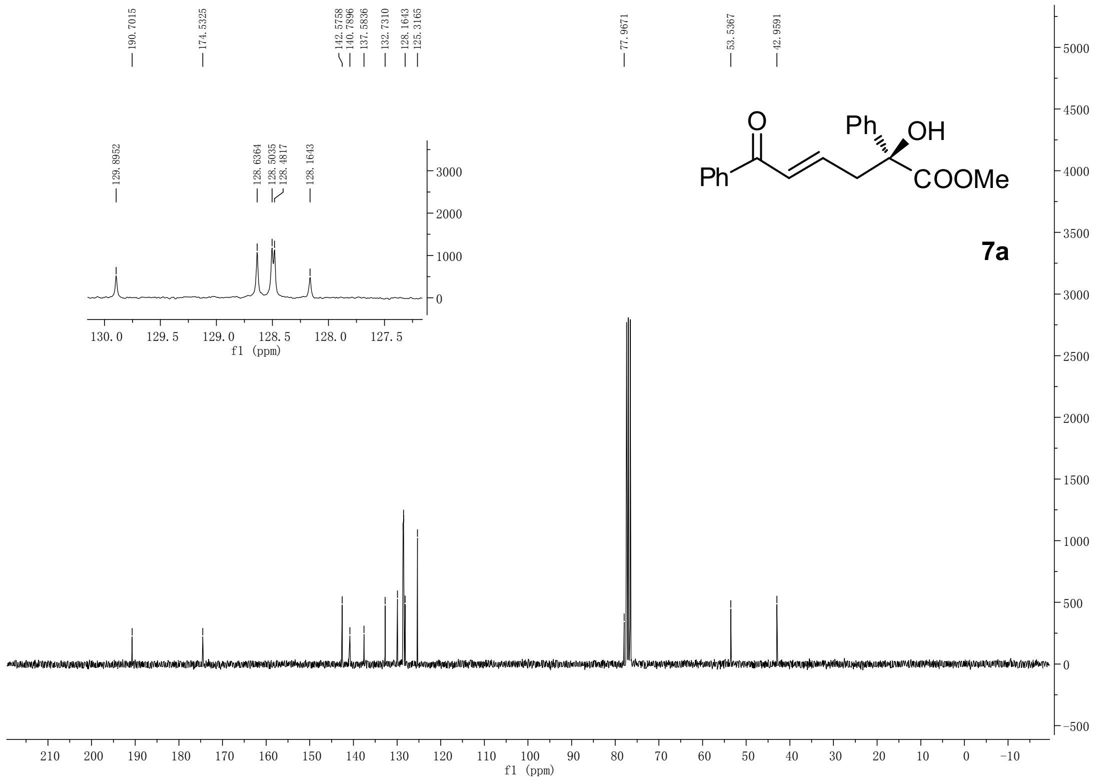

Figure S93 ^{13}C NMR spectrum of **7a.**

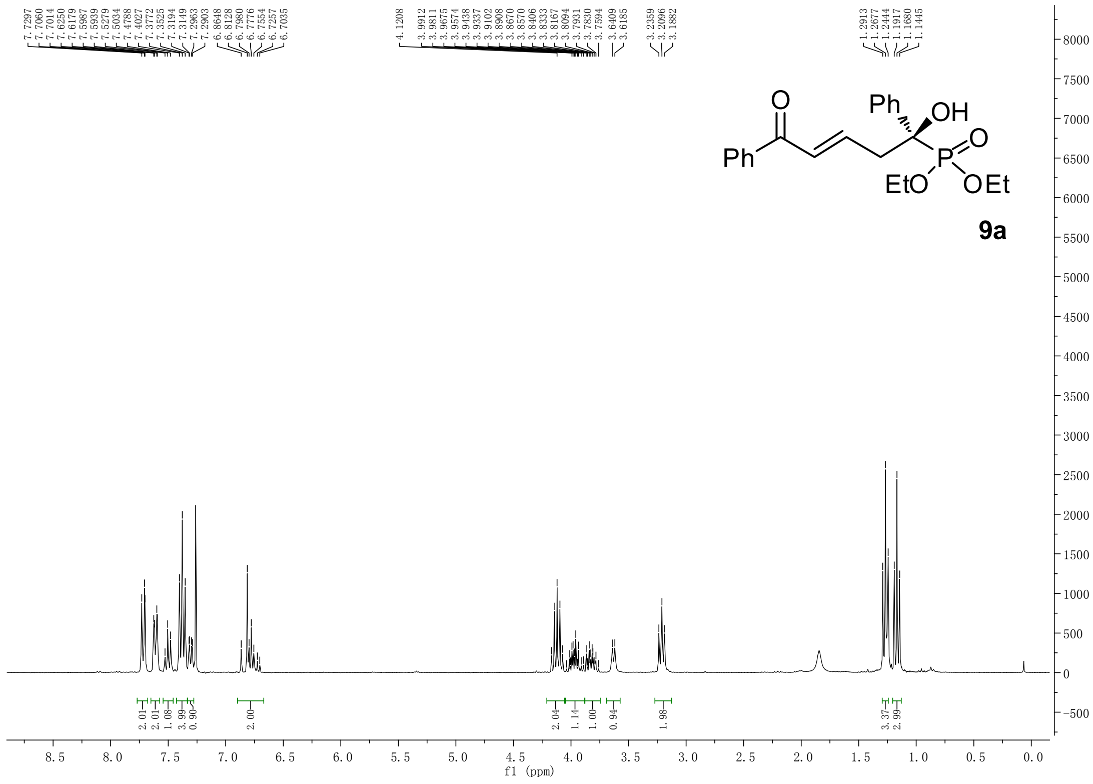

Figure S94 ^{1}H NMR spectrum of **9a.**

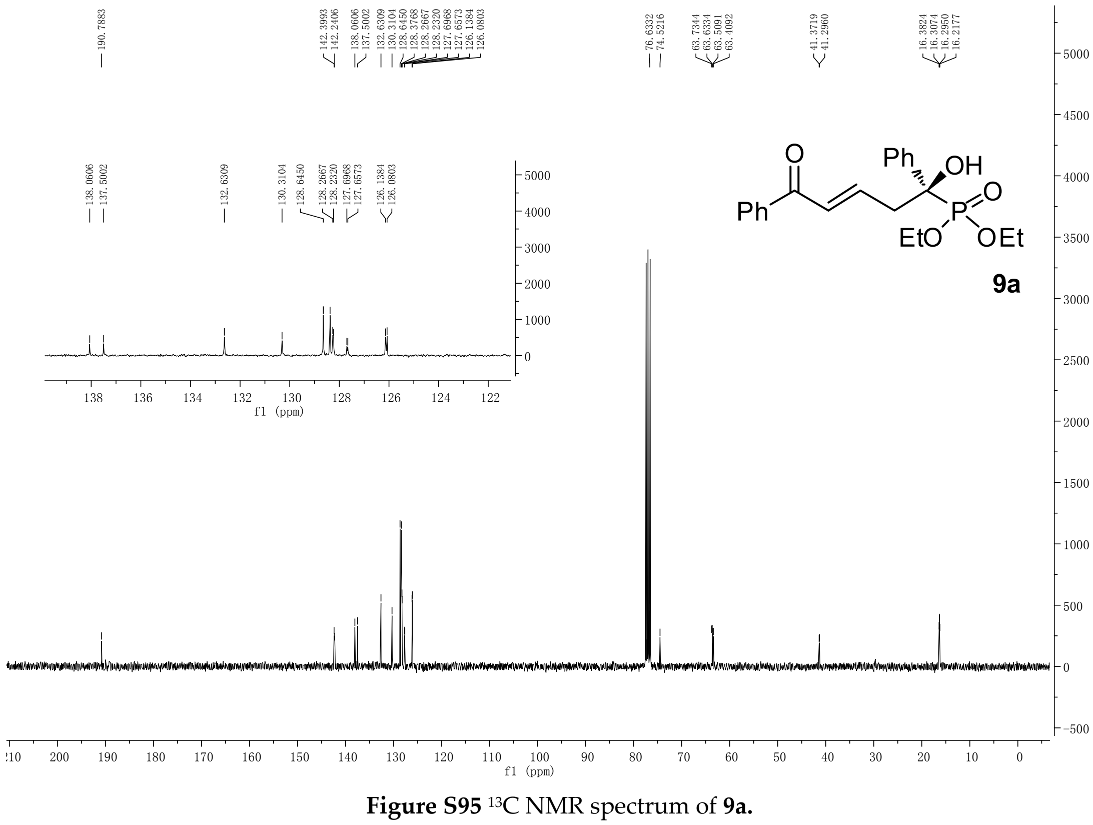

Figure S95 ^{13}C NMR spectrum of **9a.**
